# Supplementary material for: Prototyping for public health in a local context: a streamlined evaluation of a community-based weight management programme (Momenta), Northumberland, UK
Source: BMJ Open. 2019 Oct 31;9(10):e029718. doi: 10.1136/bmjopen-2019-029718 (PMC6830698; doi:10.1136/bmjopen-2019-029718)
Supplement: Supplementary data [file bmjopen-2019-029718supp001.pdf]

SUPPLEMENTARY FILE 1. Momenta session content

Week	Key topic		Key topic
**Getting started session**	Motivation for weight loss<br>Weight loss goals<br>Differences from other weight management programmes<br>Monitoring		
**Week 1**	**Snacking**<br>Fatty, sugary snacks<br>Calories from snacking<br>Healthy snack choices<br>Healthy eating patterns	**Week 7**	**Fats**<br>Reducing fat<br>Different types of fat<br>Lower-fat cooking techniques<br>Hidden fats
**Week 2**	**Heart rate**<br>Cardiovascular exercise and health<br>Cardiovascular exercise and weight management<br>Recommended amounts of CV activity<br>Heart rate and exercise	**Week 8**	**Internal triggers**<br>Introduction to internal triggers<br>Identifying internal triggers<br>Managing internal triggers
**Week 3**	**Food as fuel**<br>A balanced diet and health<br>Planning meals<br>Fibre	**Week 9**	**Active lifestyles**<br>Physical activity and health<br>Physical activity and weight management<br>Different types of physical activity
**Week 4**	**External triggers**<br>Introduction to external triggers<br>External triggers and over-eating<br>Managing triggers	**Week 10**	**Meals**<br>Positive meal environment<br>Shopping<br>Food labels
**Week 5**	**Strength**<br>Resistance activity and health<br>Resistance activity and weight management	**Week 11**	**Sugars**<br>Sugary foods and drinks and weight loss<br>Alcohol<br>Added sugars<br>Sugary drinks and appetite regulation
**Week 6**	**Breakfast**<br>Eating breakfast<br>Retraining appetite<br>Barriers to eating breakfast<br>Developing new breakfast habits<br>Beyond marketing	**Week 12**	**Eating out**<br>Challenges when eating away from home<br>Management and coping strategies<br>Moving forwards
