# Supplementary material for: Prototyping for public health in a local context: a streamlined evaluation of a community-based weight management programme (Momenta), Northumberland, UK
Source: BMJ Open. 2019 Oct 31;9(10):e029718. doi: 10.1136/bmjopen-2019-029718 (PMC6830698; doi:10.1136/bmjopen-2019-029718)
Supplement: Supplementary data [file bmjopen-2019-029718supp002.pdf]

SUPPLEMENTARY FILE 2.

Healthcare professionals' semi-structured interview guide

Semi Structured Interview Set the interviewee at ease; explain purpose of the interview; offer a better understanding of what the referral process requires to aid tier 2 weight management to be delivered in Northumberland; explanation about how the interview will be recorded; reaffirmation of consent; and how the information will be analysed and stored; rules of confidentiality / anonymity etc.

Questions:

1. Thinking about raising the weight issue, tell me about your experience of discussing weight with patients.

   Prompts

   - *How does it feel to raise weight as an issue?*
   - *Are patients open to discussing weight problems?*
   - *Do you find a difference between genders when discussing weight?*
   - *What helps you, such as the NHS Health Check Programme, to raise the issue of weight?*
   - *What else would help to raise the issue or weight in appointments?*

2. Greater retention is often achieved when patients are ready to change, tell me how you work with / assess patient's readiness to change.

   Prompts

   - *Have you had training around the cycle of change?*
   - *Do you use any specific tools or resources to assess the patient?*
   - *What would help you to assess the patient's readiness to change?*

3. Thinking the information and resources available to you during the referral, do you feel you had enough information and resources to encourage patient take up of the programme?

   Prompts

   - *Did you have enough background information?*
   - *Were the referral forms suitable / capture all the information required?*
   - *Were the patient leaflets / resources suitable?*
   - *Were there questions or issues raised that couldn't be answered?*
   - *Was the process easy to use?*
   - *What else could help you to make referrals to weight management programmes?*

4. Thinking about after you referred the patient, what happened next? (excluded after pilot)

   Prompts

   - *Did you get feedback from the weight management programme on the progress of your patient?*
   - *Did your patients achieve weight loss?*
   - *Did your patient come back and talk about their experience?*

5. What things are most likely to prevent you from making the referral a weight management programme, either commercial or Public Health funded?

   Prompts

   - *Are there barriers that you perceive, such as cost to the patient?*
   - *Are you concerned with raising the weight issue?*
   - *Is it a time factor if the patient has an appointment for anything other than a weight issue?*
   - *What would help you to overcome the barriers that prevent you from making the referral?*

6. Is there anything else that you would like to tell me about your expectations and experiences of the weight management programme?

Focus Group Topic Guide

Set group at ease; explain purpose of the focus group; offer a better understanding of what works for people in terms of tier 2 weight management and what doesn't, aiding development of an effective programme for Northumberland residents; explanation about how the focus group will be recorded; reaffirmation of consent; and how the information will be analysed and stored; rules of confidentiality / anonymity etc.

1. Tell me a bit about what sort of weight management activity you have taken part in, in the past.

   Prompts

   - *What influence have others had on your weight management?*
   - *Do you have any particular likes/dislikes of physical activity/managing weight/nutrition*
   - *Has there been anything else that has influenced your management of weight?*

2. So thinking about the weight management programme you have undertaken, how did you find out about it?

   Prompts

   - *Who / what motivated you to attend?*
   - *What made you decide that this is the right time to look at managing your weight?*
   - *Did the time of year make a difference?*

3. Thinking about your experience of when you were referred to the weight management programme, how did you find the process?

Prompts

- *What type of health professional referred you? (GP / Practice Nurse)*
- *Did you specifically attend Primary Care to discuss your weight?*
- *How was weight raised?*
- *What did the referrer explain to you about the programme? Did you get enough information?*
- *How long was it from your referral from Primary Care to the first assessment in the weight management programme; was this what you expected? Were you still motivated?*

4. How did you feel about being referred?

- *Prompts*
- *How confident did you feel about taking part in the programme?*
- *Was there anything that you were particularly looking forward to?*
- *Was there anything that you were worried about?*

5. What did you hope to achieve by taking part in the weight management programme?

Prompts

- *What were your expectations when you start attending the scheme?*
- *Have there been changes to your health that you expected happen as a result of participation?*
- *How quickly did you expect to see these changes? And did this happen?*

6. Thinking about after you were referred, what happened next?

Prompts

- *How long after referral did it take to be contacted by the Active Northumberland?*
- *What information did you receive prior to the initial consultation?*
- *How comfortable did you feel coming to the initial consultation?*

7. What influenced you most to attend the weight management programme?

Prompts

- *What did you expect from the staff?*
- *How important to you were changes in health or weight?*
- *Why were the influences raised important?*

8. What things were most likely to prevent you from attending the programme?

Prompts

- *Tell me about any worries you might have had about health issues.*
- *Tell me about any other things, such as other commitments, that might have stopped you from attending*
- *Did any of these issues arise? How did you overcome these issues?*

9. Now that you have completed the programme, tell me how did you felt about undertaking the weight management programme?

Prompts

- o *Did you achieve the health / weight outcomes you expected?*
- o *Why do you think it worked or not for you?*
- o *Do you feel you now have the tools to continue to make positive lifestyle choices?*
- o *Is there something that will prevent you to continue to make positive lifestyle choices?*

10. Is there anything else that you would like to tell me about your expectations and experiences of the weight management programme?
