# Supplementary material for: Prototyping for public health in a local context: a streamlined evaluation of a community-based weight management programme (Momenta), Northumberland, UK
Source: BMJ Open. 2019 Oct 31;9(10):e029718. doi: 10.1136/bmjopen-2019-029718 (PMC6830698; doi:10.1136/bmjopen-2019-029718)
Supplement: Author's manuscript [file bmjopen-2019-029718.draft_revisions.pdf]

BMJ Open

BMJ Open is committed to open peer review. As part of this commitment we make the peer review history of every article we publish publicly available.

When an article is published we post the peer reviewers' comments and the authors' responses online. We also post the versions of the paper that were used during peer review. These are the versions that the peer review comments apply to.

The versions of the paper that follow are the versions that were submitted during the peer review process. They are not the versions of record or the final published versions. They should not be cited or distributed as the published version of this manuscript.

BMJ Open is an open access journal and the full, final, typeset and author-corrected version of record of the manuscript is available on our site with no access controls, subscription charges or pay-per-view fees (http://bmjopen.bmj.com).

If you have any questions on BMJ Open's open peer review process please email info.bmjopen@bmj.com

Prototyping for public health in a local context: a streamlined implementation and effectiveness evaluation of a community-based weight management programme (Momenta), Northumberland, UK

Journal:	*BMJ Open*
Manuscript ID	bmjopen-2019-029718
Article Type:	Research
Date Submitted by the Author:	07-Feb-2019

Complete List of Authors:	Dodd-Reynolds, Caroline; Durham University, Department of Sport and Exercise Sciences; Durham University, Wolfson Research Institute for Health and Wellbeing Physical Activity Special Interest Group<br>Nevens, Lisa; Northumbria Healthcare NHS Foundation Trust Community Services Business Unit<br>Oliver, Emily; Durham University, Wolfson Research Institute for Health and Wellbeing Physical Activity Special Interest Group; Durham University, Deaprtment of Sport and Exercise Sciences,<br>Finch, Tracy; Northumbria University, Nursing, Midwifery and Health<br>Lake, Amelia; Department of Science, School of Science, Engineering and Design, Teesside University<br>Hanson, Coral; Edinburgh Napier University, School of Health and Social Care; Durham University, Wolfson Research Institute for Health and Wellbeing Physical Activity Special Interest Group

Keywords:	public health evaluation, prototyping, implementation, community weight management, NUTRITION & DIETETICS, exercise referral

SCHOLARONE™ Manuscripts

Prototyping for public health in a local context: a streamlined implementation and effectiveness evaluation of a community-based weight management programme (Momenta), Northumberland, UK

Caroline J Dodd-Reynolds[1,2] (corresponding author*), Lisa Nevens[3], Emily J Oliver[1,2], Tracy Finch[4], Amelia A Lake[56] Coral L Hanson[7]

[1]Department of Sport and Exercise Sciences, Durham University, Durham, DH1 3HN, UK*.

Email: caroline.dodd-reynolds@durham.ac.uk Tel: +44(0)191 3342000

[2]Wolfson Research Institute for Health and Wellbeing Physical Activity Special Interest Group, Durham University, Durham, UK.

[3]Northumbria Healthcare NHS Foundation Trust, Northumberland, UK.

[4]Department of Nursing, Midwifery and Health, Northumbria University, Newcastle upon Tyne, UK

[5]Department of Science, School of Science, Engineering and Design, Teesside University, Teesside, UK

[6]Fuse – UKCRC Centre for Translational Research in Public Health, UK

[7]School of Health and Social Care, Edinburgh Napier University, Edinburgh, UK

Word count: 4214

Keywords: Public health evaluation, protoyping, implementation, community weight management, diet, exercise referral

For peer review only - http://bmjopen.bmj.com/site/about/guidelines.xhtml

ABSTRACT

Objectives Stakeholder co-production in design of public health programmes may reduce the 'implementation gap' but is time-consuming and costly. Prototyping, iterative refining relevant to delivery context, offers a potential solution. This evaluation explored protoyping in the implementation and effectiveness of a referral-based, 12-week weight-management programme, 'Momenta', in Northumberland, UK.

Design Anonymised service evaluation data examined physiological and psychological outcomes at 12 and 52 weeks. Qualitative interviews with referring healthcare professionals and focus groups with service users explored implementation and prototyping.

Setting Two leisure centres in northeast England.

Participants Overweight and obese individuals (n=182) referred by healthcare professionals. Referring professionals (n=5) participated in individual interviews and service users (n=13) in focus groups.

Interventions Three 12-week interventions: Momenta (n=59), Momenta plus Fitness membership (Momenta-Fitness) (n=58), and Fitness membership only (n=65).

Primary and secondary outcome measures Primary outcome: weight loss. Secondary outcomes: uptake, adherence, mental wellbeing, anxiety, depression, and implementation and prototyping effectiveness.

Results 12-week weight loss [median kg, (interquartile range)] was observed for Momenta -2.9 (-5.0 to -2.0) and Momenta-Fitness -2.9 (-5.1 to -1.6) p< 0.001, but not Fitness-only. 52-week follow-up suggested persistence of weight loss for Momenta-Fitness. Uptake and 12-week adherence were higher for Momenta (84.7%, 45.8%) and Momenta-Fitness (93.1%,

For peer review only - http://bmjopen.bmj.com/site/about/guidelines.xhtml

60.3%) versus Fitness-only (75.4%, 24.6%). 12-week mental wellbeing, anxiety and depression improved in Momenta and Momenta-Fitness, remaining at 52 weeks ($p < 0.05$). Prototyping did not detrimentally impact on participants' experiences and enabled important refinements such as broadening inclusion criteria. Implementation gaps were revealed around the referral process and practitioner knowledge.

Conclusions Momenta was effective for weight loss, particularly combined with fitness membership. Prototyping aided implementation and appropriate in evaluations providing 1. A strong theoretical and empirical underpinning of the intervention; 2. use of co-production methods to allow iterative refinement during implementation.

For peer review only

For peer review only - http://bmjopen.bmj.com/site/about/guidelines.xhtml

ARTICLE SUMMARY

Strengths and limitations of this study

- This study advances understanding about whether prototyping is a cost-effective and time-efficient approach to design and implementation of public health programmes.
- This mixed methods evaluation provides insight into the implementation and effectiveness for an 'off-the-shelf' weight management programme, in a local context.
- Embedding stakeholders' views in the entire evaluation process allowed for ongoing, iterative refinement.
- A limitation to the quantitative component is the small sample size.
- Qualitative interviews and focus groups can only provide information about what participants recall about their experiences, meaning that there is a potential for recall bias.

For peer review only

INTRODUCTION

Failure to implement effective public health interventions when programmes are scaled up or transferred across contexts is widely reported.[1] Proposed approaches attempting to address this implementation gap include; effectiveness-implementation hybrid designs,[2] linking action to theory and models based on theory,[3] and application of the replicating effective programmes framework.[4] Common to all is advocacy of a developmental process reflecting on existing knowledge about the target population and planned programme prior to delivery. Engagement of service users is encouraged. Although this increases the likelihood of services meeting all stakeholder's needs, it is costly in both time and financial resources. Resulting well-designed services will be tailored to a problem that may have changed during the time spent developing the intervention. Additionally, public access is delayed. Resource-pressured public health services must therefore consider pragmatic alternatives to service design and implementation. In this paper, we explore a novel evaluation approach to these implementation challenges, focusing on a problem high on the public health agenda: obesity and overweight.

Targeting elevated weight status is a public health priority, obesity being a recognised risk factor for many physical and psychological health outcomes.[5-9] In England for example, obesity and overweight are associated with 30,000 deaths and an estimated National Health Service cost of £6.1 billion per annum.[10] Globally, countries with higher income inequalities tend to have higher rates of obesity.[11] Excess weight is also associated with widening social and economic deprivation,[12] with calls to improve the effectiveness of behaviour change interventions for low-income groups.[13] There is a clear need for effective public health programmes that can be refined according to local need. This evaluation focuses on

For peer review only - http://bmjopen.bmj.com/site/about/guidelines.xhtml

Northumberland, in northeast England. Northumberland is one of the lowest ranked counties in England by Gross Value Added per capita (£16,140).[14] Unemployment is higher (5.5% versus 4.8%) than the England average[15] and Northumberland public health spend per person is £53, compared to a £59 national average.[16] 63.8% of adults are classified as having excess weight, higher than the national average of 61.3%.[17]

The need for innovation within public health has been postulated, shifting away from the traditional linear pre-conceived and evidence-based model.[18] One alternative is prototyping where projects test innovations iteratively, with ongoing refinement considering the interplay between a programme and its delivery context.[19] A small number of studies to date, for example in drug prevention[20] and web-based support of long-term weight loss[21] have demonstrated efficiencies when including elements of prototyping within programme development. Such an approach seems particularly well-suited to weight management, where there are many examples of 'good' practice, or effectiveness, but no clear consensus on 'best' practice at service-delivery level. There is also limited understanding of how tailoring programmes or interventions to local contexts may impact on effectiveness. This evaluation has particular value therefore in testing a prototyping approach for a weight management programme delivered at local authority level. Specifically, we take an 'off-the-shelf' programme, 'Momenta'[22] and evaluate its implementation and effectiveness in a challenging context. Emergent findings will facilitate understanding not only of whether the programme is adaptable, demonstrating promise for 'scaling up', but more importantly whether the prototyping approach can be a resource-effective way of informing and refining public health delivery.

For peer review only - http://bmjopen.bmj.com/site/about/guidelines.xhtml

METHODS

The prototyping process: local context and evaluation design

A local authority health needs assessment identified a gap in provision for a lifestyle-based weight management referral programme within Northumberland. Overweight and obese adults were at the time eligible for referral to the Northumberland exercise referral scheme (ERS). Although the ERS doubled as a weight management intervention, previous evaluation demonstrated modest weight loss[23] and a body mass index (BMI) >30 kg/m^2 was negatively associated with adherence.[24] Thus 'Momenta' was commissioned for local adaptation and delivery. Momenta is an evidence-based, outcome-driven behavioural programme designed to be delivered by fitness professionals in a leisure environment.[22] Developed by the MEND childhood weight management programme[25] designers, this 12-week programme aims to facilitate weight loss by engaging participants in 12 key behaviours broadly encompassing psychology, diet and physical activity. It was offered free to service users.

The local Leisure Trust was commissioned to deliver a pilot Momenta programme. Stakeholder meetings were held with Leisure Trust managers, delivery staff and Momenta programme developers. Members of the evaluation team (CDR, EO) provided guidance on evaluation design and light touch advice about tools to explore effectiveness. The evaluation was thus co-produced to ensure a robust framework, whilst meeting strategic local needs. For example, commissioners were concerned about meeting recruitment targets for an existing tier three weight management service for pre-bariatric patients and Momenta was initially commissioned for overweight patients only (BMI 25.0-29.9 kg/m^2), although this was later amended. Furthermore, commissioners were keen to consider accessibility of provision and

For peer review only - http://bmjopen.bmj.com/site/about/guidelines.xhtml

wished to explore the effect of offering free gym, swimming and fitness class membership. The evaluation was designed to accommodate this.

The pilot programme was delivered at two leisure sites situated within the 20% and 50% most deprived neighbourhoods in the country. Six General Practice (GP) surgeries, identified as the best referrers to the existing ERS, were asked to refer suitable patients to Momenta. The programme manager and the public health improvement manager (LN) attended practice meetings to articulate referral criteria and disseminate advertising materials. Attendance varied from two to all practice staff, meaning that in some surgeries knowledge of the programme was reliant on dissemination by those who attended.

A mixed methods evaluation was agreed between the evaluation team and commissioners. Quantitative and qualitative components were conducted concurrently and had equal status.[26]

Quantitative evaluation component

Programme providers allocated service users into one of three comparison groups:
a) Combined Momenta plus fitness membership (Momenta-Fitness);
b) Momenta;
c) Fitness membership (Fitness-only).
Referrals by healthcare professionals (HCPs) were made via a standardised form to the appropriate leisure site. Due to maximum recommended Momenta group size, referrals were split into delivery cohorts of 15, with groups rolling through March 2015 to April 2016.

For peer review only - http://bmjopen.bmj.com/site/about/guidelines.xhtml

Outcome measures included anthropometric measurements to determine weight change. Secondary well-being outcome measures were of specific interest to commissioners. Prior to programme commencement the Leisure Trust, in conjunction with the Momenta programme designer and members of the evaluation team, held a training day for delivery staff. Although staff were qualified to deliver Momenta, extra bespoke training (including role-play scenarios and problem-solving discussions) was delivered by the clinical psychologist who designed Momenta. The evaluation team (CDR, CH) trained delivery staff in international standard anthropometric techniques[27] and familiarised them with other evaluation measures.

Age, gender and postcode (for index of multiple deprivation, IMD) were recorded by referring HCPs on the referral form. Employment status, level of education, cohort wave and programme group were recorded by leisure staff, who also measured height, weight, and waist circumference at baseline and programme end. Measures were taken in at least duplicate, using standardised tools. Body mass index was calculated and classified according to WHO guidelines.[28] The Warwick-Edinburgh Mental Well-being Scale,[29] and the Hospital Anxiety and Depression Scale[30] were administered at each time-point. Attendance at Momenta and leisure centre usage was monitored via swipe-card tracking. 52 weeks after commencing the programme, participants were invited to attend a follow-up session, where leisure staff repeated physiological and psychological measures. Programme providers collected and collated quantitative data and provided an anonymised dataset to the evaluation team for analysis.

For peer review only - http://bmjopen.bmj.com/site/about/guidelines.xhtml

Qualitative evaluation component

Implementation effectiveness for the referral process was explored through semi-structured interviews with referring HCPs (undertaken at referring surgeries) and focus groups with service users (in leisure centres). All were conducted by LN during March-July 2015, as part of her Public Health Masters degree (which contained qualitative methods training), mentored by TF, an experienced qualitative researcher. Questions are included in supplementary file 1. Data were audio-recorded. Results are reported using the Consolidated criteria for Reporting Qualitative research guidelines[31]

Practice managers from all six referring surgeries were sent an invitation for staff to take part. Individual correspondence was sent to those agreeing. Interviews aimed to explore HCPs' referral experiences; raising weight issues; assessing readiness to change; marketing and referral materials; and the referral process. Interview questions were pilot tested with public health colleagues to assess timing and ensure validity. One question (*Thinking about after you referred the patient, what happened next?*) was omitted after piloting as it was realised HCPs would not have had patient feedback at that point. Interviews lasted on average 26 minutes and were transcribed verbatim. Data were analysed following each interview, with developing themes considered to determine whether questions required refinement. Initial themes generated from the first two interviews did not change and thus questions remained constant, although prompts were added.

During the initial assessment session for the first wave of referrals, all (n = 39) were invited to participate in a series of focus groups at programme-end to explore experiences. Emphasis was placed on the referral process, initial expectations and experiences of participation; how

For peer review only - http://bmjopen.bmj.com/site/about/guidelines.xhtml

weight issues were raised by HCPs; time from referral to initial assessment; and facilitators and barriers to taking part. Focus groups lasted between 26 and 44 minutes.

Patient and public involvement

Commissioners, deliverers and service users were involved in the iterative evaluation.

Data analyses

The anonymised quantitative dataset was analysed using PSAW Statistics V.22. Descriptive statistics were calculated for age, gender, IMD, employment status, initial BMI, leisure site, level of education, and uptake and adherence. Distribution and normality of measures (weight, BMI, waist circumference, psychological wellbeing and attendance) were assessed using Shapiro Wilk tests and median and interquartile range (IQR) scores calculated for each group at baseline and 12 weeks (attendance, 12 weeks only). Kruskal-Wallis H tests were used to determine between group differences at baseline and 12 weeks for viable data. Wilcoxon-signed rank tests examined differences between baseline and 12-week scores. Data available at 52 weeks (n = 37) were analysed separately.

Qualitative data were audio-recorded and transcribed by LN using a thematic process.[32] Data were organised according to concepts, key themes and developing categories. Data coding was discussed with TF, allowing comparison of data interpretation and subsequent coding refinement. Evolving key themes were refined through the analysis process and subsequent cross-sectional thematic labelling of data, thus generating deeper understanding. Where

For peer review only - http://bmjopen.bmj.com/site/about/guidelines.xhtml

possible, key phrases or expressions identified from interviews and focus groups were retained within coding and thematic labelling. A public health colleague helped to verify interpretations of the data and appropriateness of codes applied. Once initial interviews were coded this framework was applied to remaining data. Notes taken during focus groups helped to contextualise when developing themes and included information about dynamics within groups, such as influence, disagreement, humour and peer exposure.

RESULTS

Between December 2014 and March 2016, the programme received 182 referrals and was delivered in four cohorts across leisure sites. Due to initial low levels of recruitment, the first cohort did not start until March 2015. Referrals were mainly female (83%) and 30.6% lived in the 20% most deprived areas (table 1).

For peer review only

Table 1. Demographic characteristics of referrals (n=165)

	Median	IQR
**Age (years)**	53	24
**Gender (n=167)**	**n**	**%**
Male	29	17.4%
Female	138	82.6%
**Initial BMI category (kg/m^2, n=150)**		
25.0-29.9	40	26.7%
30.0-34.9	73	48.6%
35.0-39.9	27	18.0%
≥40.0	10	6.7%
**Leisure site (n=170)**		
Leisure site 1 (IMD quintile 2)	80	44.7%
Leisure site 2 (IMD quintile 3)	99	55.3%
**Index of multiple deprivation (n=170)**		
20% most deprived	52	30.6%
21-40%	41	24.1%
41-60%	20	11.8%
61-80%	20	11.8%
20% least deprived	37	21.6%
**Employment status (n=123)**		
Employed full time	37	30.1%
Employed part time	24	19.5%
Retired	51	41.5%
Claiming incapacity benefit	5	4.1%
Claiming job seekers allowance	6	4.9%
**Level of education (n=127)**		
Primary	15	11.8%
Secondary (O level/GCSE)	35	27.6%
Secondary (A level)	26	20.5%
Further education (HND)	25	19.7%
Bachelors or equivalent	21	16.5%
Masters or equivalent	5	3.9%

Programme Effectiveness

Of all referrals, 153 (84%) attended the baseline measurement session and 78 (51% of those who started) attended the 12-week measurement session. Uptake and adherence varied by programme group (table 2).

For peer review only

For peer review only - http://bmjopen.bmj.com/site/about/guidelines.xhtml

Table 2. Programme uptake, adherence and attendance.

**Uptake and adherence**	**Momenta plus fitness**		**Momenta only**		**Fitness only**	
Number referred		58		59		65
Uptake* (n, %)		54 (93.1%)		50 (84.7%)		49 (75.4%)
Uptake Adherence** (n, %)		35 (64.8%)		27 (54.0%)		16 (32.7%)
Overall adherence*** (n, %)		58 (60.3%)		59 (45.8%)		65 (24.6%)
**Momenta session attendance**	**Momenta plus fitness**		**Momenta only**		**Fitness only**	
	**n**	**Median (IQR)**	**n**	**Median (IQR)**		
Uptake	54	9.0 (7.3)	50	9.0 (8.0)		
Dropouts	19	3.0 (3.0)	23	3.0 (5.0)		N/A
Adherers	35	10.0 (2.0)	27	11.0 (1.3)		
**Exercise session attendance**	**Momenta plus fitness**		**Momenta only**		**Fitness only**	
	**n**	**Median (IQR)**	**n**	**Median (IQR)**	**n**	**Median (IQR)**
Uptake	54	7.0 (16.3)	50	0.0 (4.5)	49	0.0 (1.5)
Dropouts	19	0.0 (1.0)	23	0.0 (0.0)	33	0.0 (0.0)
Adherers	35	10.0 (14.0)	26	0.0 (5.0)	16	4.5 (18.0)

Uptake* *participant attended baseline assessment;* ***Uptake adherence**** *% of participants who attended the baseline assessment who also attended the 12-week assessment;* ***Overall adherence***** *% of all those referred who attended both baseline and 12-week assessment*

Physiological and psychological data were not normally distributed. No significant differences were found between programme groups either at baseline or at 12 weeks, for any measures. Significant within-group differences between baseline and 12 weeks were evident for weight, BMI and waist circumference for Momenta-Fitness, and Momenta (Table 3). Follow-up analysis at 52-weeks (available sub-sample) showed changes were maintained for Momenta-Fitness (n =18) only.

For peer review only

For peer review only - http://bmjopen.bmj.com/site/about/guidelines.xhtml

Table 3. Weight, BMI and waist circumference change.

End of programme results	Median (IQR) Baseline	Median (IQR) 12 weeks	z	p	Median (IQR) Change
**Weight (kg)**					
Momenta plus fitness (n=35)	88.9 (80.5 - 100.0)	88.0 (77.2 - 95.8)	-4.531	<0.001	-2.9 (-5.1 - -1.6)
Momenta only (n=26)	87.8 (74.5 - 77.0)	83.3 (74.5 - 92.5)	-4.344	<0.001	-2.9 (-5.0 - -2.0)
Fitness only (n=15)	76.2 (71.6 - 86.9)	76.6 (70.4 - 84.6)	-0.879	0.379	0.0 (-3.2 - 1.0)
**BMI (kg/m²)**					
Momenta plus fitness (n=35)	32.0 (30.3 - 35.7)	31.3 (29.2 – 35.3)	-4-494	<0.001	-1.1 (-1.9 - -0.6)
Momenta only (n=26)	32.0 (30.0 - 34.5)	31.3 (28.6 – 33.6)	-4.356	<0.001	-1.2 (-1.6 - -0.8)
Fitness only (n=14)	29.2 (27.3 - 33.0)	29.7 (27.0 – 33.3)	-0.454	0.650	0.1 (-1.2 - +0.4)
**Waist circumference (cm)**					
Momenta plus fitness (n=35)	106.0 (98.0 - 115.0)	99.0 (93.0 - 110.0)	-4.996	<0.001	-7.0 (-9.5 - -5.0)
Momenta only (n=25)	108.0 (99.5 - 114.5)	101.0 (93.8 - 111.5)	-4.166	<0.001	-5.0 (-7.3 - -2.5)
Fitness only (n=11)	90.0 (87.0 – 95.0)	91.0 (90.0 - 96.0)	0.358	0.650	1.0 (-3.0 - 3.0)
**52-week follow-up**	**Median (IQR) Baseline**	**Median (IQR) 52 weeks**	**z**	**p**	**Median (IQR) Change**
**Weight (kg)**					
Momenta plus fitness (n=18)	95.2 (87.1 - 101.4)	91.4 (82.7 - 95.9)	-3.006	<0.001	-4.8 (-6.2 - -1.5)
Momenta only (n=16)	84.7 (72.3 - 95.2)	82.7 (73.2 - 94.6)	-1.533	0.120	-0.7 (-7.6 - 0.8)
**BMI (kg/m²)**					
Momenta plus fitness (n=18)	32.0 (30.49 - 35.1)	30.8 (28.7 - 34.0)	-3.157	<0.05	-1.7 (-2.0 - -0.6)
Momenta only (n=16)	31.7 (29.3 – 33.9)	31.1 (26.7 – 33.6)	-1.603	0.109	-0.3 (-2.3 - 0.3)
**Waist circumference (cm)**					
Momenta plus fitness (n=18)	109.0 (101.0 - 114.8)	100.5 (94.8 - 107.3)	-3.221	<0.001	-6.0 (-13.3 - -1.75)
Momenta only (n=16)	106.0 (94.5 - 115.8)	103.5 (98.5 - 113.3)	-0.780	0.938	-2.5 (-9.0 - -10.0)

** Fitness only n=3 therefore no 52-week calculations*

Significant improvement in mental wellbeing, and reductions in depression and anxiety, were evident between baseline and 12 weeks for Momenta-Fitness, and Momenta (Table 4). The magnitude of change was relatively small but functionally and clinically meaningful. For example, the median value for anxiety for Momenta dropped from a moderate symptomology to a not symptomatic classification. 52-week sub-sample analysis showed that significant improvements for wellbeing and depression were maintained for Momenta-Fitness (n=18), and wellbeing and anxiety for Momenta (n=16).

For peer review only - http://bmjopen.bmj.com/site/about/guidelines.xhtml

Table 4. Wellbeing, anxiety and depression measures change.

End of programme results	Median (IQR) Baseline	Median (IQR) 12 weeks	z	p	Median (IQR) Change
**Mental wellbeing scale**					
Momenta plus fitness (n=29)	46.0 (40.0 - 51.5)	53.0 (40.0 - 51.5)	3.810	<0.001	5.0 (1.5 - 12.0)
Momenta only (n=23)	49.0 (39.0 - 58.0)	55.0 (51.0 - 63.0)	2.818	<0.05	6.0 (-1.0 - 10.0)
Fitness only (n=13)	47.0 (40.5 - 59.5)	46.0 (42.0 - 63.5)	0.157	0.875	0.0 (-4.0 - 5.0)
**Anxiety scale**					
Momenta plus fitness (n=28)	5.5 (4.0 - 9.8)	4.5 (2.0 - 7.0)	-3.027	<0.001	-1.0 (-3.0 - 1.0)
Momenta only (n=23)	8.0 (6.0 - 10.0)	4.0 (2.5 - 9.0)	-2.329	<0.05	-1.0 (-3.0 - 0.0)
Fitness only (n=13)	8.0 (3.5 - 10.0)	6.0 (4.0 - 9.0)	-0.499	0.618	-1.0 (-2.0 - 2.0)
**Depression scale**					
Momenta plus fitness (n=28)	5.5 (3.3 - 8.0)	2.0 (1.0 - 6.0)	-3.214	<0.05	-2.5 (-4.8 - -0.3)
Momenta only (n=23)	5.0 (3.0 - 7.5)	3.0 (1.0 - 5.0)	-3.049	<0.05	-1.0 (-4.5 - 1.0)
Fitness only (n=13)	4.0 (2.0 - 8.5)	2.0 (2.0 - 7.0)	-1.226	0.220	-2.0 (-4.5 - 0.0)

52-week follow-up	Median (IQR) Baseline	Median (IQR) 52 weeks	z	p	Median (IQR) Change
**Mental wellbeing scale**					
Momenta plus fitness (n=15)	44.0 (39.0 - 52.0)	55.0 (48.0 - 59.0)	2.984	<0.05	5.0 (3.0 - 15.0)
Momenta only (n=13)	58.0 (47.5 - 59.0)	56.0 (54.0 - 63.5)	2.282	<0.05	4.0 (0.5 - 6.5)
**Anxiety scale**					
Momenta plus fitness (n=15)	6.0 (2.0 - 10.0)	2.0 (1.0 - 7.0)	-1.785	0.074	-3.0 (-6.0 - 0.0)
Momenta only (n=15)	7.0 (4.0 - 9.0)	5.0 (1.0 - 8.0)	-1.990	<0.05	-3.0 (-4.0 - 0.0)
**Depression scale**					
Momenta plus fitness (n=15)	7.0 (3.3 - 11.3)	3.5 (1.0 - 6.0)	-2.908	<0.05	-3.5 (-6.3 - -0.8)
Momenta only (n=15)	4.0 (1.0 - 6.0)	3.0 (1.0 - 4.0)	-0.762	0.446	0.0 (-2.0 - 1.0)

** Fitness only n=3 therefore no 52-week calculations*

Overall, the results suggested those who participated in the two groups incorporating Momenta, had enhanced physical and psychological health indicators from baseline, whereas those who had only free fitness membership did not. There is some evidence that the combination of Momenta and fitness membership produces the best outcomes at 52 weeks.

For peer review only

For peer review only - http://bmjopen.bmj.com/site/about/guidelines.xhtml

Implementation effectiveness: reflections from referring healthcare professionals

Five face-to-face semi-structured interviews took place with HCPs across five referring surgeries: two GPs, two Practice Nurses and one Health Care Assistant. HCPs perceived that four key themes influenced the effectiveness of programme implementation: (i) difficulties raising weight with patients, (ii) how gender affected patient engagement, (iii) availability of information and resources, and (iv) additional barriers constraining programme promotion.

Raising the issue of weight with patients:

Concerns about raising weight may have contributed to slow recruitment, with nurses and healthcare assistants expressing unease, *'not really up to me… well I talk about it if they want to…. Better if they [patients] bring it up.'* (Interview 2, Healthcare Assistant). GPs seemed more comfortable raising weight with patients, but with the caveat that this is easier in the context of a longer-term GP/patient relationship.

> *'the people I see I've known for a very long time… it's the rapport you have…if I'd never met anyone before and they came in for a sore throat I'm not going to say you're fat…If there was someone I'd known for a long time and it seemed relevant…I'd mention it.'* (Interview 5, GP).

Gender and engagement in the referral process:

Gender was highlighted as influencing the referral process, women being more likely than men to seek support for weight. This may help explain the low rate of referral for males (17%):

For peer review only - http://bmjopen.bmj.com/site/about/guidelines.xhtml

'More women talk about it…men don't really talk about weight…I do mention weight to men if I'm doing a well man [sic] but it doesn't come up really…it's a woman thing…' (Interview 3, Practice Nurse).

Availability of information and resources:

Several interviewees highlighted training needs around programme information and resources, (e.g., additional programme information would help to engage patients). For example, the GPs both discussed the longstanding ERS and stated they needed to become more familiar with Momenta, as they had with the ERS:

'when we get opportunities to do things in the practice we normally discuss it, let everyone know where appropriate forms and information is and it's in your head…that didn't happen with this and I don't know why that was.' (Interview 5, GP).

All HCPs interviewed felt the referral leaflet (provided by programme providers) was important in the process, either as a tool to promote the intervention or to convey information to patients:

'The leaflet was good, bright…explained the programme and patients like taking a leaflet away.' (Interview 3, Practice Nurse)

For peer review only

Additional Barriers to Engagement:

Several sub-themes highlighted additional barriers to the referral process. The most prominent sub-themes were around initial BMI referral criteria (25.0-29.9 kg/m^2) and delayed programme start. Both implementation factors were beyond the control of the referrers, but consequently amended through iterative refinement during the prototyping process following early data analysis. Both were reported by practice nurses as exacerbating each other:

> '*we were referring but then it didn't start so people were not sure what was happening [pause]…Think it was more people were needed to start…but you know if the BMI was higher then there would have been more*.' (Interview 3, Practice Nurse).

In one case, a decision was taken to relax the referral criteria, *'…31.5 [kg/m^2]…was just outside so I just referred him.*' (Interview 4, GP).

Programme location was perceived by HCPs to overcome an existing barrier to the tier three weight management programme, as Momenta was '*round the corner for people,*' as opposed to '*a bit far away at the hospital.'* Cost barriers were also discussed, both with reference to the patient, *'in this sort of area…cost…, if you've got to pay it's a barrier.'* (Interview 4, GP), and to expected targets from Clinical Commissioning Groups (CCG),

> *'we are constantly told by the CCG that we must keep down on numbers and that if there are costs attached to this referral that would definitely impact… and that would be for all practices.'* (Interview 5, GP)

For peer review only - http://bmjopen.bmj.com/site/about/guidelines.xhtml

Implementation effectiveness: reflections from participants

Three focus groups allowed programme participant voices to be heard: three females and one male from Momenta (focus group 1), three males and three females from Momenta-Fitness (focus group 2) and three females (one of whom emailed her views separately) from Fitness-only. Across the groups, 12 participants reported having lost weight and one reported weight gain. Three themes developed: (i) outcomes of the programme, (ii) facilitators and barriers to engagement, and (iii) raising the issues of weight with HCPs.

For

Outcomes of the programme:

Focus group findings aligned closely with quantitative outcomes in terms of the physical and psychological benefits of participation: *'[I've] lost a good bit of weight. It's been very positive for me… I'm feeling a lot more active…'* (Momenta-Fitness, Participant 5). Participants reported a sense of weight loss achievement, increased physical activity levels, and positive mood states. In addition, elements of the Momenta programme were perceived as facilitating engagement, including the *'group feeling… I looked forward to it,'* (Momenta-Fitness, Participant 4), the *'information that we got every week… so very well planned.'* (Momenta-Fitness, Participant 3) and the ongoing support e.g., *'she 'phoned me the other day to see if I was coming,'* (Momenta-Fitness, Participant 4). Momenta participants reflected back on, and identified and discussed lifestyle factors that related to their initial weight gain (e.g., *'I did the usual thing… I started eating toffees,'* Momenta-Fitness, Participant 5*),* demonstrating both self-awareness and an openness to discussing the topic.

For peer review only - http://bmjopen.bmj.com/site/about/guidelines.xhtml

Facilitators and barriers to engagement:

One participant reported being initially excluded but later allowed to take part, and others raised concerns that the initial BMI threshold for referral was too low, '*was a little bit high, BMI…managed to get it down... [and then] the doctor put us forward,'* (Momenta, Participant 2). Data also indicated the importance of subsidised access, particularly important in the context of a deprived region such as this, e.g., '*I also joined Weight Watchers for short period of time but found the classes too expensive,*' (Fitness-only, Participant 3, emailed response).

Raising the issue of weight with HCPs:

Some data did suggest implementation was problematic, however, this focused exclusively on the referral process. Participants overwhelmingly felt that they had opened the conversation about weight, as opposed to discussions being initiated by HCPs (e.g., '*my glucose levels were quite high but nobody ever said that I was overweight*,' Momenta-Fitness, Participant 4*).* In addition, participants perceived limitations in HCPs' knowledge of intervention components ('*she [nurse] didn't know anything about it*,' Fitness-only, Participant 1), something with potential to impact on likelihood of referral, and participants' expectations of programme success.

DISCUSSION

We explored 'prototyping', as a cost-effective and time-efficient approach to public health evaluation, via an 'off-the-shelf' weight management programme implemented in a local context of mixed and high deprivation. Participation in Momenta and Momenta-Fitness

For peer review only - http://bmjopen.bmj.com/site/about/guidelines.xhtml

resulted in 12-week weight loss for those who completed the programme. Free fitness membership without the weight-management programme was poorly engaged with and did not result in weight change. After a year, weight reductions equivalent to 5% were maintained for Momenta-Fitness, greater than seen for some commercial weight-loss programmes,[33] although a limited sample was available for follow-up analyses. Providing free access to fitness facilities alongside the behaviour change programme was potentially a factor, allowing for continuous and self-driven behaviour change[34] and sustaining optimal changes in adiposity over 12 months.[35] Swipe card monitoring during the initial 12-week period indicated that fitness sessions were accessed an average 10 occasions for this group, whereas no access was apparent for Momenta, despite Momenta sessions being held in leisure centres. This may be important for community providers making decisions about delivery location. Both Momenta groups reported improved wellbeing, and reduced anxiety and depression at 12-weeks suggesting that the behavioural intervention drives this effect. This is consistent with previous work reporting co-varying changes in weight loss, depression, and quality of life in weight management services.[36] It is unclear whether the primary mechanism was weight loss, or the wider social benefits of participation. Both were valued in the qualitative data. Maintenance of significant improvements in wellbeing for these groups at 52 weeks is important given previously evidenced associations between poor mental health, and obesity and overweight status.[37]

Experiences of both referrers and referrals highlighted that HCPs needed to be better-informed and more confident raising weight-related conversations. Whilst patient-led action is desirable, staff reluctance to raise weight issues may mean that opportunities for engagement of less knowledgeable or motivated patients will be missed. The problematic positioning of GPs within obesity care has been highlighted previously,[38] with a range of

strategies to change HCPs' behaviour resulting in little or no change to patients' weight. A practical training need is highlighted for those working at the patient-practitioner interface, however communication with patients about weight may well be hindered by the 'stigma' attached to obesity.[39] This has wider implications for patient outcomes and requires further exploration through the implementation process. Additionally, HPCs need better understanding of referral-based public health programmes offered. Despite efforts of programme and public health managers, awareness was reportedly low for some referring professionals. We suggest consideration of resource-efficient ways to signpost both HPCs and patients themselves as part of the implementation process.

This programme was delivered across a social gradient in a region with low health indices and areas of high deprivation. Some issues in relation to inequalities and service access for future community-based weight management programmes were highlighted. Only 17% of referrals to Momenta were males. Gender bias in weight management referral has been reported elsewhere,[40-41] and interviews showed that practitioners struggled to raise the topic of weight with male patients. Alternative referral strategies have been employed in other settings in an attempt to overcome this.[42] Marketing in other community spaces, or targeted postal referrals could be explored in future implementation. The initial decision to restrict referral to overweight-only substantially impacted on referral rates, with HCPs and referrals indicating they felt limited until this restriction was reversed. Had this continued, worsening health inequalities may have been an unintended consequence, something to be actively avoided within public health programmes [43]. The roles of, and interactions between, those operating in the 'system' (i.e. the context within which the intervention operates) must be considered at the point of implementation to minimise any impact from unintended consequences.[44] In practical terms, this may be through continued dialogue with

For peer review only - http://bmjopen.bmj.com/site/about/guidelines.xhtml

commissioners, referring professionals and referrals themselves, something which prototyping evaluation allows.

Given that no systematic problems emerged with participants' experiences of the programme itself, our findings lend support to a streamlined approach to involvement of all stakeholders in programme implementation. Furthermore, the prototyping evaluation format allowed for changes following programme commencement, suggesting that this route offers opportunities for off-the-shelf programmes to be pragmatically moulded to local context, in real-time. However, emergence of some negative experiences of referral suggests that prototyping can be problematic without networks or channels for ensuring key outcomes are widely communicated to relevant actors. Overall, the evaluation demonstrated that a balance is needed to allow quick and efficient adaptation of off-the-shelf programmes, but with focused professional user engagement in the early stages of development. Some confidence in the approach was derived from the strong theoretical grounding of the programme; we would consider it unwise to adopt such a streamlined approach otherwise. The prototyping approach had particular utility given that project resources were limited and meant that issues were identified and acted upon rapidly. While the programme may have progressed similarly without this, prototyping provided a greater structure for, and confidence in, on-going refinements. This was achieved via the support provided by academics, public health practitioners and providers. Fundamentally, adopting a prototyping approach enabled the delivery of a new service to an in-need population, alongside the generation of initial evidence of local effectiveness. A minimum of 1 kg weight-loss at 3 months, and 0.7 kg at 12-months have been suggested as thresholds to influence decisions over commissioning of weight-loss services.[33] Momenta met and indeed exceeded these and shows particular promise when implemented in conjunction with free fitness provision.

For peer review only - http://bmjopen.bmj.com/site/about/guidelines.xhtml

Demonstrating effectiveness is of limited use, however, unless a successful programme in one area may be adapted and implemented to suit a different context, for example through sharing local-level knowledge, interactions and behaviours of actors within different parts of that system.[45] The process for scaling-up of effective health interventions to broader policy and practice takes years[46] and certainly within the obesity literature, has been dominated by initiatives that consider effectiveness but not implementation across specific settings.[47-48] Whilst recognising the small sample size in this evaluation, the prototyping approach shows promise in successfully testing innovations iteratively. Furthermore, ongoing refinement considering the interplay between a programme and its delivery context could be built into larger public health interventions.

CONCLUSION

This evaluation extends the literature by exploring prototyping for a complex problem, community weight-management, in a challenging setting, demonstrating streamlined implementation of an 'off-the-shelf' weight management programme. We demonstrate good outcome effectiveness for 'Momenta', particularly in conjunction with a free fitness offer. This resource-effective approach is highly relevant in the context of health inequalities and public health sector funding constraints. We recommend prototyping in public health evaluation providing: 1. The original programme has a strong theoretical and empirical basis, and 2. All stakeholders shape implementation, with evaluation sought during early delivery phases to iteratively refine the process.

For peer review only - http://bmjopen.bmj.com/site/about/guidelines.xhtml

Acknowledgements: Many thanks to Jordan Bell, Nicole Rowley and Ross Davison, who collected and collated raw quantitative data.

Author Contributions: CDR, CH and EO contributed to design of the quantitative evaluation, data analysis and preparation the final document. LN contributed to design and analyses of the qualitative evaluation component. TF contributed to qualitative design and analysis and preparation of the final document. AL contributed to design of the quantitative evaluation and preparation of the final document.

Funding: The quantitative evaluation component was supported by funding from the Wolfson Research Institute for Health and Wellbeing, Durham University. Amelia Lake is a member of Fuse, the Centre for Translational Research in Public Health (www.fuse.ac.uk). Fuse is a UK Clinical Research Collaboration (UKCRC) Public Health Research Centre of Excellence. Funding for Fuse from the British Heart Foundation, Cancer Research UK, Economic and Social Research Council, Medical Research Council, the National Institute for Health Research, under the auspices of the UKCRC, is gratefully acknowledged. The views expressed in this paper do not necessarily represent those of the funders or UKCRC. The funders had no role in study design, data collection and analysis, decision to publish, or preparation of the manuscript.

Competing interests: CH is a former employee of the Leisure Trust and was instrumental in initiating the evaluation. She was subsequently employed as a research assistant at Durham University, however was not involved in any data collection or entry, only accessing an anonymised database submitted to the University. LN was a Public Health Improvement

For peer review only - http://bmjopen.bmj.com/site/about/guidelines.xhtml

Manager within the Public Health Team and had responsibility for commissioning the Momenta programme. The qualitative evaluation component was submitted in partial fulfilment of her Masters in Public Health at Newcastle University.

Participant consent: Consent was obtained for face-to-face interviews and focus groups. Service users were informed in writing of the nature of the quantitative service evaluation and how to withdraw from it. The presented data are anonymised with risk of identification low.

Ethics approval: Ethical advice was sought from the local Research Manager of North of England Commissioning Support, and this project was classed as a service evaluation in line with National Research Ethics Service guidance.

Data sharing statement: No additional data are available

For peer review only

For peer review only - http://bmjopen.bmj.com/site/about/guidelines.xhtml

REFERENCES

1. Haines A, Kuruvilla S, Borchert M. Bridging the implementation gap between knowledge and action for health. (Theme Papers). *Bull World Health Organ* 2004;82:724.

2. Curran GM, Bauer M, Mittman B, *et al*. Effectiveness-implementation Hybrid Designs: Combining Elements of Clinical Effectiveness and Implementation Research to Enhance Public Health Impact. *Med Care* 2012;50:217-26.

3. Sales A, Smith J, Curran G, *et al*. Models, Strategies, and Tools: Theory in Implementing Evidence-Based Findings into Health Care Practice. *J Gen Int Med* 2006;21:S43-S9.

4. Kilbourne AM, Neumann MS, Pincus HA, *et al*. Implementing evidence-based interventions in health care: application of the replicating effective programs framework. *Implement Sci* 2007;2:42.

5. Meisinger C, Döring A, Thorand B, *et al*. Body fat distribution and risk of type 2 diabetes in the general population: are there differences between men and women? The MONICA/KORA Augsburg Cohort Study. *Am J Clin Nutr* 2006;84:483-9.

6. Bhaskaran K, Douglas I, Forbes H, *et al*. Body-mass index and risk of 22 specific cancers: a population-based cohort study of 5·24 million UK adults. *Lancet* 2014;384:755-65.

For peer review only - http://bmjopen.bmj.com/site/about/guidelines.xhtml

7. Zhang C, Rexrode KM, Van Dam RM, *et al*. Abdominal obesity and the risk of all-cause, cardiovascular, and cancer mortality: sixteen years of follow-up in US women. *Circ* 2008;117:1658-67.

8. Kolotkin RL, Andersen JR. A systematic review of reviews: exploring the relationship between obesity, weight loss and health-related quality of life. *Clin Obes* 2017;7:273-89.

9. Simon GE, Von Korff M, Saunders K, *et al*. Association between obesity and psychiatric disorders in the us adult population. *Arch Gen Psychiatry* 2006;63(7):824-30.

10. Public Health England. *Health matters: obesity and the food environment environment.* UK: Public Health England, 2017. https://www.gov.uk/government/publications/health-matters-obesity-and-the-food-environment/health-matters-obesity-and-the-food-environment--2. (Accessed 10 Aug 2018)

11. World Health Organisation. *Global status report on noncommunicable diseases 2014*. Geneva: World Health Organisation, 2014. https://apps.who.int/iris/bitstream/handle/10665/148114/9789241564854_eng.pdf;jsessionid=A043D0E63AACA67752CC3294A1EBD7A3?sequence=1. (Accessed 04 Feb 2019)

12. Bambra C, Hillier F, Cairns J, *et al*. How effective are interventions at reducing socioeconomic inequalities in obesity among children and adults? Two systematic reviews. *Public Health Res* 2015;3.

For peer review only - http://bmjopen.bmj.com/site/about/guidelines.xhtml

13. Bull ER, Dombrowski SU, McCleary N, *et al*. Are interventions for low-income groups effective in changing healthy eating, physical activity and smoking behaviours? A systematic review and meta-analysis. *BMJ Open*. 2014;4:e006046.

14. Office for National Statistics. *Dataset: Regional gross value added (balanced) by combined authority in the UK*. UK: Office for National Statistics, 2017. https://www.ons.gov.uk/economy/grossvalueaddedgva/datasets/regionalgrossvalueaddedbalancedbycombinedauthorityintheuk. (Accessed 10 Aug 2018)

15. Public Health England. *Work, worklessness and health: local infographic tool*. UK: Public Health England, 2018. https://www.gov.uk/government/publications/work-worklessness-and-health-local-infographic-tool. (Accessed 10 Aug 2018)

16. Northumberland County Council. *Public Health Service Statement 2017/18.* Northumberland: Northumberland County Council, 2017. https://northumberland.gov.uk/NorthumberlandCountyCouncil/media/About-the-Council/Performance/Service%20Statements%202016/2017-Public-Health-Service-Statement-2017-18.pdf. (Accessed 10 Aug 2018)

17. Public Health England. *Local Authority Health Profiles*. UK: Public Health England, 2019. https://fingertips.phe.org.uk/profile/health-profiles/data#page/0/gid/1938132701/pat/6/par/E12000001/ati/102/are/E06000057/iid/10101/age/169/sex/4. (Accessed 01 Feb 2019)

18. Lister C, Payne H, Hanson CL, *et al*. The Public Health Innovation Model: Merging Private Sector Processes with Public Health Strengths. *Front Public Health* 2017;5:192.

For peer review only - http://bmjopen.bmj.com/site/about/guidelines.xhtml

19. Parry GJ, Carson-Stevens A, Luff DF, *et al*. Recommendations for evaluation of health care improvement initiatives. *Acad Pediatr* 2013 Nov-Dec;13(6 Suppl):S23-30.

20. Hawkins J, Madden K, Fletcher A, *et al*. Development of a framework for the co-production and prototyping of public health interventions. *BMC Public Health* 2017;17:689.

21. Stevens VJ, Funk KL, Brantley PJ, *et al*. Design and implementation of an interactive website to support long-term maintenance of weight loss. *J Med Internet Res* 2008;10(1):e1.

22. Discover Momenta Ltd. *Momenta.* UK: Discover Momenta Ltd, 2013: http://discovermomenta.com/. (Accessed 10 Aug 2018)

23. Hanson CL. *Advancing understanding of effective exercise on referral: a mixed methods evaluation of the Northumberland scheme*. PhD Thesis, Durham University, 2017. http://etheses.dur.ac.uk/12162/. (Accessed 10 Aug 2018)

24. Hanson CL, Allin LJ, Ellis JG, *et al*. An evaluation of the efficacy of the exercise on referral scheme in Northumberland, UK: association with physical activity and predictors of engagement. A naturalistic observation study. *BMJ Open* 2013;3:e002849

25. Sacher PM, Kolotourou M, Chadwick PM, *et al*. Randomized Controlled Trial of the MEND Program: A Family-based Community Intervention for Childhood Obesity. *Obes* 2012;18:S62-S8.

For peer review only - http://bmjopen.bmj.com/site/about/guidelines.xhtml

26. Leech N, Onwuegbuzie A. A typology of mixed methods research designs. *Qual Quant* 2009;43:265-75.

27. Marfell-Jones M, Olds T, Stewart A, *et al*. *International Standards for Anthropometric Assessment (2007)*. Potchefstroom, South Africa: International Society for the Advancement of Kinathropometry, 2007.

28. World Health Organisation. *BMI Classification 2006.* Geneva: World Health Organisation, 2006. http://apps.who.int/bmi/index.jsp?introPage=intro_3.html. (Accessed 10 Aug 2018)

29. Clarke A, Friede T, Putz R, *et al*. Warwick-Edinburgh Mental Well-being Scale (WEMWBS): Validated for teenage school students in England and Scotland. A mixed methods assessment. *BMC Public Health* 2011;11:487.

30. Zigmond AS, Snaith RP. The Hospital Anxiety and Depression Scale. *Acta Psychiatr Scand* 1983;67:361-70.

31. Tong A, Sainsbury P, Craig J. Consolidated criteria for reporting qualitative research (COREQ): a 32-item checklist for interviews and focus groups. *Int J Qual. Health Care* 2007;19:349–57

32. Braun V, Clarke V. Using thematic analysis in psychology. *Qual Res Psychol* 2006;3:77-101.

For peer review only - http://bmjopen.bmj.com/site/about/guidelines.xhtml

33. Madigan CD, Daley AJ, Lewis AL, *et al*. Which weight-loss programmes are as effective as Weight Watchers®? *Brit J Gen Pract* 2014;64(620):e128-e136.

34. Mastellos N, Gunn LH, Felix LM, *et al*. Transtheoretical model stages of change for dietary and physical exercise modification in weight loss management for overweight and obese adults. *Cochrane Database Syst Rev* 2014;2:CD008066

35. Garrow JS, Summerbell CD. Meta-analysis: effect of exercise, with or without dieting, on the body composition of overweight subjects. *Eur J Clin Nutr* 1995;49:1-10.

36. Wright F, Boyle S, Baxter K, *et al*. Understanding the relationship between weight loss, emotional well-being and health-related quality of life in patients attending a specialist obesity weight management service. *J Health Psychol* 2013;18:574-86.

37. Somerville R, McKenzie K, Eslami S, *et al*. Poor mental health in severely obese patients is not explained by the presence of comorbidities. *Clin Obes* 2014;5:12-21.

38. Flodgren G, Goncalves BDC, Summerbell CD. Interventions to change the behaviour of health professionals and the organisation of care to promote weight reduction in children and adults with overweight or obesity. *Cochrane Database of Syst Rev* 2017;11:CD000984.

39. Phelan SM, Burgess DJ, Yeazel MW, *et al*. Impact of weight bias and stigma on quality of care and outcomes for patients with obesity. *Obes Rev* 2015;16:319-26.

For peer review only

For peer review only - http://bmjopen.bmj.com/site/about/guidelines.xhtml

40. Logue J, Allardice G, Gillies M, *et al*. Outcomes of a specialist weight management programme in the UK National Health Service: prospective study of 1838 patients. *BMJ Open* 2014;4:e003747.

41. Lebrun LA, Chowdhury J, Sripipatana A, *et al*. Overweight/obesity and weight-related treatment among patients in U.S. federally supported health centers. *Obes Res Clin Pract* 2013;7:e377-e90.

42. Ahern AL, Aveyard P, Boyland EJ, *et al*. Inequalities in the uptake of weight management interventions in a pragmatic trial: an observational study in primary care. *Br J Gen Pract* 2016;66(645):e258-63.

43. Adams J, Mytton O, White M, *et al*. Why Are Some Population Interventions for Diet and Obesity More Equitable and Effective Than Others? The Role of Individual Agency. *PLoS Med* 2016;13(4).

44. Medical Research Council. *Developing and evaluating complex interventions: new guidance.* UK: Medical Research Council, 2006. https://mrc.ukri.org/documents/pdf/developing-and-evaluating-complex-interventions/. (Accessed 10 Aug 2018)

45. Curtis S, Oven K, Wistow J, *et al*. Adaptation to extreme weather events in complex health and social care systems: The example of older people's services in England. *Environ Plann C: Politics and Space* 2018;36:67-91.

For peer review only

For peer review only - http://bmjopen.bmj.com/site/about/guidelines.xhtml

46. Milat AJ, King L, Newson R, *et al*. Increasing the scale and adoption of population health interventions: experiences and perspectives of policy makers, practitioners, and researchers. *Health Res Policy and Systems* 2014;12:18.

47. Jebb S. *Dusting off Foresight's obesity report.* Foresight Blog UK: Government Office for Science, 2017. https://foresightprojects.blog.gov.uk/2017/10/04/dusting-off-foresights-obesity-report/. (Accessed 01 Feb 2019)

48. Wolfenden L, Goldman S, Stacey FG *et al*. Strategies to improve the implementation of workplace-based policies or practices targeting tobacco, alcohol, diet, physical activity and obesity. *Cochrane Database of Syst Rev* 2018;11:CD012439.

For peer review only

For peer review only - http://bmjopen.bmj.com/site/about/guidelines.xhtml

SUPPLEMENTARY FILE 1.

Healthcare professionals' semi-structured interview guide

Semi Structured Interview Set the interviewee at ease; explain purpose of the interview; offer a better understanding of what the referral process requires to aid tier 2 weight management to be delivered in Northumberland; explanation about how the interview will be recorded; reaffirmation of consent; and how the information will be analysed and stored; rules of confidentiality / anonymity etc.

Questions:

1. Thinking about raising the weight issue, tell me about your experience of discussing weight with patients.

   Prompts

   - *How does it feel to raise weight as an issue?*
   - *Are patients open to discussing weight problems?*
   - *Do you find a difference between genders when discussing weight?*
   - *What helps you, such as the NHS Health Check Programme, to raise the issue of weight?*
   - *What else would help to raise the issue or weight in appointments?*

2. Greater retention is often achieved when patients are ready to change, tell me how you work with / assess patient's readiness to change.

   Prompts

   - *Have you had training around the cycle of change?*
   - *Do you use any specific tools or resources to assess the patient?*
   - *What would help you to assess the patient's readiness to change?*

For peer review only

3. Thinking the information and resources available to you during the referral, do you feel you had enough information and resources to encourage patient take up of the programme?

Prompts

- *Did you have enough background information?*
- *Were the referral forms suitable / capture all the information required?*
- *Were the patient leaflets / resources suitable?*
- *Were there questions or issues raised that couldn't be answered?*
- *Was the process easy to use?*
- *What else could help you to make referrals to weight management programmes?*

4. Thinking about after you referred the patient, what happened next? (excluded after pilot)

Prompts

- *Did you get feedback from the weight management programme on the progress of your patient?*
- *Did your patients achieve weight loss?*
- *Did your patient come back and talk about their experience?*

5. What things are most likely to prevent you from making the referral a weight management programme, either commercial or Public Health funded?

Prompts

- *Are there barriers that you perceive, such as cost to the patient?*
- *Are you concerned with raising the weight issue?*
- *Is it a time factor if the patient has an appointment for anything other than a weight issue?*
- *What would help you to overcome the barriers that prevent you from making the referral?*

For peer review only

6. Is there anything else that you would like to tell me about your expectations and experiences of the weight management programme?

Focus Group Topic Guide

Set group at ease; explain purpose of the focus group; offer a better understanding of what works for people in terms of tier 2 weight management and what doesn't, aiding development of an effective programme for Northumberland residents; explanation about how the focus group will be recorded; reaffirmation of consent; and how the information will be analysed and stored; rules of confidentiality / anonymity etc.

1. Tell me a bit about what sort of weight management activity you have taken part in, in the past.

   Prompts

   - *What influence have others had on your weight management?*
   - *Do you have any particular likes/dislikes of physical activity/managing weight/nutrition*
   - *Has there been anything else that has influenced your management of weight?*

2. So thinking about the weight management programme you have undertaken, how did you find out about it?

   Prompts

   - *Who / what motivated you to attend?*
   - *What made you decide that this is the right time to look at managing your weight?*
   - *Did the time of year make a difference?*

For peer review only

3. Thinking about your experience of when you were referred to the weight management programme, how did you find the process?

Prompts

- *What type of health professional referred you? (GP / Practice Nurse)*
- *Did you specifically attend Primary Care to discuss your weight?*
- *How was weight raised?*
- *What did the referrer explain to you about the programme? Did you get enough information?*
- *How long was it from your referral from Primary Care to the first assessment in the weight management programme; was this what you expected? Were you still motivated?*

4. How did you feel about being referred?

- *Prompts*
- *How confident did you feel about taking part in the programme?*
- *Was there anything that you were particularly looking forward to?*
- *Was there anything that you were worried about?*

5. What did you hope to achieve by taking part in the weight management programme?

Prompts

- *What were your expectations when you start attending the scheme?*
- *Have there been changes to your health that you expected happen as a result of participation?*
- *How quickly did you expect to see these changes? And did this happen?*

6. Thinking about after you were referred, what happened next?

Prompts

For peer review only

- *How long after referral did it take to be contacted by the Active Northumberland?*
- *What information did you receive prior to the initial consultation?*
- *How comfortable did you feel coming to the initial consultation?*

7. What influenced you most to attend the weight management programme?

Prompts

- *What did you expect from the staff?*
- *How important to you were changes in health or weight?*
- *Why were the influences raised important?*

8. What things were most likely to prevent you from attending the programme?

Prompts

- *Tell me about any worries you might have had about health issues.*
- *Tell me about any other things, such as other commitments, that might have stopped you from attending*
- *Did any of these issues arise? How did you overcome these issues?*

9. Now that you have completed the programme, tell me how did you felt about undertaking the weight management programme?

Prompts

- *Did you achieve the health / weight outcomes you expected?*
- *Why do you think it worked or not for you?*
- *Do you feel you now have the tools to continue to make positive lifestyle choices?*
- *Is there something that will prevent you to continue to make positive lifestyle choices?*

For peer review only

10. Is there anything else that you would like to tell me about your expectations and experiences of the weight management programme?

For peer review only

COREQ (COnsolidated criteria for REporting Qualitative research) Checklist

A checklist of items that should be included in reports of qualitative research. You must report the page number in your manuscript where you consider each of the items listed in this checklist. If you have not included this information, either revise your manuscript accordingly before submitting or note N/A.

Topic	Item No.	Guide Questions/Description	Reported on Page No.
**Domain 1: Research team and reflexivity**			
Personal characteristics			
Interviewer/facilitator	1	Which author/s conducted the interview or focus group?	
Credentials	2	What were the researcher's credentials? E.g. PhD, MD	
Occupation	3	What was their occupation at the time of the study?	
Gender	4	Was the researcher male or female?	
Experience and training	5	What experience or training did the researcher have?	
Relationship with participants			
Relationship established	6	Was a relationship established prior to study commencement?	
Participant knowledge of the interviewer	7	What did the participants know about the researcher? e.g. personal goals, reasons for doing the research	
Interviewer characteristics	8	What characteristics were reported about the inter viewer/facilitator? e.g. Bias, assumptions, reasons and interests in the research topic	
**Domain 2: Study design**			
Theoretical framework			
Methodological orientation and Theory	9	What methodological orientation was stated to underpin the study? e.g. grounded theory, discourse analysis, ethnography, phenomenology, content analysis	
Participant selection			
Sampling	10	How were participants selected? e.g. purposive, convenience, consecutive, snowball	
Method of approach	11	How were participants approached? e.g. face-to-face, telephone, mail, email	
Sample size	12	How many participants were in the study?	
Non-participation	13	How many people refused to participate or dropped out? Reasons?	
Setting			
Setting of data collection	14	Where was the data collected? e.g. home, clinic, workplace	
Presence of non-participants	15	Was anyone else present besides the participants and researchers?	
Description of sample	16	What are the important characteristics of the sample? e.g. demographic data, date	
Data collection			
Interview guide	17	Were questions, prompts, guides provided by the authors? Was it pilot tested?	
Repeat interviews	18	Were repeat inter views carried out? If yes, how many?	
Audio/visual recording	19	Did the research use audio or visual recording to collect the data?	
Field notes	20	Were field notes made during and/or after the inter view or focus group?	
Duration	21	What was the duration of the inter views or focus group?	
Data saturation	22	Was data saturation discussed?	
Transcripts returned	23	Were transcripts returned to participants for comment and/or	

For peer review only

Topic	Item No.	Guide Questions/Description	Reported on Page No.
		correction?	
**Domain 3: analysis and findings**			
Data analysis			
Number of data coders	24	How many data coders coded the data?	
Description of the coding tree	25	Did authors provide a description of the coding tree?	
Derivation of themes	26	Were themes identified in advance or derived from the data?	
Software	27	What software, if applicable, was used to manage the data?	
Participant checking	28	Did participants provide feedback on the findings?	
Reporting			
Quotations presented	29	Were participant quotations presented to illustrate the themes/findings? Was each quotation identified? e.g. participant number	
Data and findings consistent	30	Was there consistency between the data presented and the findings?	
Clarity of major themes	31	Were major themes clearly presented in the findings?	
Clarity of minor themes	32	Is there a description of diverse cases or discussion of minor themes?	

Developed from: Tong A, Sainsbury P, Craig J. Consolidated criteria for reporting qualitative research (COREQ): a 32-item checklist for interviews and focus groups. *International Journal for Quality in Health Care*. 2007. Volume 19, Number 6: pp. 349 – 357

Once you have completed this checklist, please save a copy and upload it as part of your submission. DO NOT include this checklist as part of the main manuscript document. It must be uploaded as a separate file.

For peer review only

BMJ Open

Prototyping for public health in a local context: a streamlined evaluation of a community-based weight management programme (Momenta), Northumberland, UK

Journal:	*BMJ Open*
Manuscript ID	bmjopen-2019-029718.R1
Article Type:	Original research
Date Submitted by the Author:	23-May-2019
Complete List of Authors:	Dodd-Reynolds, Caroline; Durham University, Department of Sport and Exercise Sciences; Durham University, Wolfson Research Institute for Health and Wellbeing Physical Activity Special Interest Group<br>Nevens, Lisa; Northumbria Healthcare NHS Foundation Trust Community Services Business Unit<br>Oliver, Emily; Durham University, Wolfson Research Institute for Health and Wellbeing Physical Activity Special Interest Group; Durham University, Deaprtment of Sport and Exercise Sciences,<br>Finch, Tracy; Northumbria University, Nursing, Midwifery and Health<br>Lake, Amelia; Department of Science, School of Science, Engineering and Design, Teesside University<br>Hanson, Coral; Edinburgh Napier University, School of Health and Social Care; Durham University, Wolfson Research Institute for Health and Wellbeing Physical Activity Special Interest Group
**Primary Subject Heading**:	Public health
Secondary Subject Heading:	Nutrition and metabolism
Keywords:	public health evaluation, prototyping, implementation, community weight management, NUTRITION & DIETETICS, exercise referral

SCHOLARONE™
Manuscripts

Prototyping for public health in a local context: a streamlined evaluation of a community-based weight management programme (Momenta), Northumberland, UK

Caroline J Dodd-Reynolds[1,2] (corresponding author*), Lisa Nevens[3], Emily J Oliver[1,2], Tracy Finch[4], Amelia A Lake[56] Coral L Hanson[7]

[1]Department of Sport and Exercise Sciences, Durham University, Durham, DH1 3HN, UK*.
Email: caroline.dodd-reynolds@durham.ac.uk Tel: +44(0)191 3342000
[2]Wolfson Research Institute for Health and Wellbeing Physical Activity Special Interest Group, Durham University, Durham, UK.
[3]Northumbria Healthcare NHS Foundation Trust, Northumberland, UK.
[4]Department of Nursing, Midwifery and Health, Northumbria University, Newcastle upon Tyne, UK
[5]Department of Science, School of Science, Engineering and Design, Teesside University, Teesside, UK
[6]Fuse – UKCRC Centre for Translational Research in Public Health, UK
[7]School of Health and Social Care, Edinburgh Napier University, Edinburgh, UK

Word count: 4214

Keywords: Public health evaluation, protoyping, implementation, community weight management, diet, exercise referral

For peer review only

For peer review only - http://bmjopen.bmj.com/site/about/guidelines.xhtml

ABSTRACT

Objectives Stakeholder co-production in design of public health programmes may reduce the 'implementation gap' but can be time-consuming and costly. Prototyping, iterative refining relevant to delivery context, offers a potential solution. This evaluation explored implementation and lessons learned for a 12-week referral-based weight-management programme, 'Momenta', along with feasibility of an iterative prototyping evaluation framework.

Design Mixed methods evaluation: analysis of anonymised service data provided for physiological and psychological outcomes (12 and 52 weeks), qualitative exploration of implementation with referrers and service users.

Setting Two leisure centres in Northumberland, northeast England.

Participants Individuals (n=182) with BMI>24.9 kg/m^2, referred by healthcare professionals. Individual interviews with referring professionals (n=5) and focus groups with service users (n=13).

Interventions Three 12-week programme iterations: Momenta (n=59), Momenta-Fitness membership (Momenta-Fitness) (n=58), and Fitness membership only (n=65).

Primary and secondary outcome measures Weight loss, BMI, waist circumference, uptake, adherence, mental wellbeing, anxiety, depression. Qualitative themes developed through stakeholder-engagement.

Results 12-week weight loss [median kg, (interquartile range)] was observed for Momenta -2.9 (-5.0 to -2.0) and Momenta-Fitness -2.9 (-5.1 to -1.6) $p < 0.001$, but not Fitness-only. Preliminary 52-week follow-up suggested weight loss maintenance for Momenta-Fitness.

For peer review only - http://bmjopen.bmj.com/site/about/guidelines.xhtml

Uptake and 12-week retention were higher for Momenta (84.7%, 45.8%) and Momenta-Fitness (93.1%, 60.3%) versus Fitness-only (75.4%, 24.6%). 12-week mental wellbeing, anxiety and depression improved in Momenta and Momenta-Fitness, remaining at 52 weeks ($p < 0.05$). Service users reported positive experiences of Momenta. Implementation gaps were revealed around the referral process itself and practitioner knowledge. Prototyping enabled important iterative refinements such as broadening inclusion criteria.

Conclusions Momenta has potential for weight loss, particularly when offered with a fitness membership. Identification of issues with referral process enabled real-time iterative refinement to address some of these, whilst lessons learned may be of value for local implementation of 'off-the-shelf' weight management packages more generally.

For peer review only

ARTICLE SUMMARY

Strengths and limitations of this study

- This study advances understanding about whether prototyping is a time-efficient and cost-effective approach to design and implementation of public health programmes.
- This mixed methods evaluation provides insight into the implementation of an 'off-the-shelf' weight management programme, in a local context.
- Embedding stakeholders' views throughout the entire evaluation process allowed for ongoing, iterative refinement.
- A limitation to the quantitative component is the small sample size and findings should be interpreted with caution.
- Qualitative interviews and focus groups can only provide information about what participants recall about their experiences, meaning that there is a potential for recall bias.

For peer review only

INTRODUCTION

Failure to implement effective public health interventions when programmes are scaled up or transferred across contexts is widely reported.[1] Proposed approaches attempting to address this implementation gap include; effectiveness-implementation hybrid designs,[2] linking action to theory and models based on theory,[3] and application of the replicating effective programmes framework.[4] Common to all is advocacy of a developmental process reflecting on existing knowledge about the target population and planned programme prior to service delivery. Furthermore, engagement of service users is encouraged at all stages of intervention and evaluation design in MRC guidance.[5] Although this increases the likelihood of services meeting all stakeholder's needs, concerns about the practical, personal, and professional costs of co-production have been raised.[6]. Resulting well-designed services will be tailored to a problem that may have changed during the time spent developing the intervention. Additionally, public access may be delayed. Resource-pressured public health services must therefore consider pragmatic alternatives to service design and implementation. In this paper, we explore a novel evaluation approach to these implementation challenges, focusing on a problem high on the public health agenda: obesity and overweight.

Targeting elevated weight status is a public health priority, obesity being a recognised risk factor for many physical and psychological health outcomes.[7-11] In England for example, obesity and overweight are associated with 30,000 deaths and an estimated National Health Service cost of £6.1 billion per annum.[12] Globally, countries with higher income inequalities tend to have higher rates of obesity.[13] Excess weight is also associated with widening social and economic deprivation,[14] with calls to improve the effectiveness of behaviour change interventions for low-income groups.[15] There is a clear need for effective public health

For peer review only - http://bmjopen.bmj.com/site/about/guidelines.xhtml

programmes that can be refined according to local need. This evaluation focuses on Northumberland, in northeast England. Northumberland is one of the lowest ranked counties in England by Gross Value Added per capita (£16,140).[16] Unemployment is higher (5.5% versus 4.8%) than the England average[17] and Northumberland public health spend per person is £53, compared to a £59 national average.[18] 63.8% of adults are classified as having excess weight, higher than the national average of 61.3%.[19]

The need for innovation within public health has been postulated, shifting away from the traditional linear pre-conceived and evidence-based model.[20] Parry and colleagues[21] highlight a need to explore how a programme works, but also the context and requirements for any adaptations. One such approach is prototyping[22] where projects test innovations iteratively, with ongoing refinement considering the interplay between a programme and its delivery context. Evaluation and public health teams are able to communicate at all stages of the programme, with evaluation recommendations incorporated via a rapid-cycle basis[21]. A small number of studies to date, for example in drug prevention[22] and web-based support of long-term weight loss[23] have demonstrated efficiencies (including time, adaptation to context and cost) when including elements of prototyping within programme development. Such an approach seems particularly well-suited to weight management, where there are many examples of 'good' practice, or effectiveness, but no clear consensus on 'best' practice at service-delivery level. There is also limited understanding of how 'scaling up' and adapting of programmes or interventions to local contexts may impact on effectiveness. This evaluation has particular value therefore in testing a prototyping approach for a weight management programme, delivered and adapted 'in real-time', at local authority level.
The aim was to explore implementation of an 'off-the-shelf' weight management programme, Momenta[24], in a challenging context. Specific objectives were to identify preliminary

For peer review only - http://bmjopen.bmj.com/site/about/guidelines.xhtml

programme effectiveness, explore local implementation, and consider feasibility of the iterative prototyping evaluation framework.

METHODS

The prototyping process: local context and evaluation design

A local authority health needs assessment identified a gap in provision for a lifestyle-based weight management referral programme within Northumberland. Adults with overweight or obesity were at the time eligible for referral to the Northumberland exercise referral scheme (ERS), however previous evaluation demonstrated modest weight loss[25] and body mass index (BMI) >30 kg/m^2 was negatively associated with adherence.[26] Thus 'Momenta' was commissioned for local adaptation and delivery. Momenta is an outcome-driven programme incorporating evidence-based behaviour change techniques, that is designed to be delivered by fitness professionals in a leisure environment.[24] Developed by the MEND childhood weight management programme[27] designers, this 12-week programme aims to facilitate weight loss by engaging participants in 12 key behaviours broadly encompassing psychology, diet and physical activity. Briefly, Momenta sessions explored topics using interactive and experiential learning techniques including brainstorming, group activities and discussion, quizzes and games. At the end of each session, participants set goals focusing on one of the 12 key behaviours. At the beginning of each session, the group discussed the previous weeks' goals by exchanging stories and brainstorming challenges. All interventions were free to service users.

For peer review only - http://bmjopen.bmj.com/site/about/guidelines.xhtml

The local Leisure Trust was commissioned to deliver a pilot Momenta programme. Commissioners and providers had ideas about alternative delivery options and due to an established academic relationship, asked the study team for advice about robust evaluation that would allow for feedback in real time and at the end of the pilot. Stakeholder meetings were held with Public Health staff (n=2), Leisure Trust managers (n=3), delivery staff (n=2) and Momenta programme developers (n=2). As part of the prototyping process, members of the evaluation team (CDR, EO) provided guidance on evaluation design and light touch advice about tools to explore preliminary effectiveness. The evaluation was thus co-produced to ensure a robust framework, whilst meeting strategic local needs. For example, commissioners were concerned about meeting recruitment targets for an existing specialist weight management service used mainly for pre-bariatric patients and Momenta was initially commissioned for patients with BMI 25.0-29.9 kg/m^2, although this was later amended. Furthermore, commissioners were keen to consider accessibility of provision and wished to explore offering free gym, swimming and fitness class membership. The evaluation was designed to accommodate this.

The programme was delivered at two leisure sites situated within the 20% and 50% most deprived neighbourhoods in the country. Six General Practice (GP) surgeries, identified as the best referrers to the existing ERS, were asked to refer suitable patients to Momenta. The programme manager and the public health improvement manager (LN) attended practice meetings to articulate referral criteria and disseminate advertising materials. Attendance varied from two to all practice staff, meaning that in some surgeries knowledge of the programme was reliant on dissemination by those who attended.

For peer review only - http://bmjopen.bmj.com/site/about/guidelines.xhtml

A mixed methods evaluation was agreed between the evaluation team and commissioners. Quantitative and qualitative components were conducted concurrently and had equal status.[28] Prototyping allowed for iterative changes to be made to the implementation and delivery of the programme in real time. We reflect upon these in the results and discussion.

Quantitative evaluation component

Referrals by healthcare professionals (HCPs) were via a standardised form to the appropriate leisure site. Programme providers allocated service users into one of three comparison groups:

a) Combined Momenta plus fitness membership (Momenta-Fitness);

b) Momenta;

c) Fitness membership (Fitness only).

Participants were allocated into groups in order of receipt (the first referral form received was allocated to Momenta-Fitness, the second form to Momenta, the third form to fitness only etc.). The provider then contacted participants by telephone to arrange attendance. If a participant was unable to attend the allocated group, (e.g. due to inconvenient session times) provider allocated them to a different group after discussion. Due to maximum recommended Momenta group size, referrals were split into delivery cohorts of 15, with groups rolling through March 2015 to April 2016.

Outcome measures included anthropometric measurements to determine weight change. Secondary well-being outcome measures were of specific interest to commissioners. Prior to programme commencement the Leisure Trust, in conjunction with the Momenta programme designer and members of the evaluation team, held a training day for delivery staff. Although

For peer review only - http://bmjopen.bmj.com/site/about/guidelines.xhtml

staff were qualified to deliver Momenta, extra bespoke training (including role-play scenarios and problem-solving discussions) was delivered by the clinical psychologist who designed Momenta. The evaluation team (CDR, CH) trained delivery staff in international standard anthropometric techniques[29] and familiarised them with other evaluation measures.

Age, gender and postcode (for index of multiple deprivation, IMD) were recorded by referring HCPs on the referral form. Employment status, level of education, cohort wave and programme group were recorded by leisure staff, who also measured weight and stature (without shoes or bulky clothing) and waist circumference at baseline and programme end. Measures were taken in at least duplicate, using standardised tools in accordance with international standards[29] using SECA 761 scales, a Leicester portable stadiometer and anthropometry tape. Body mass index was calculated and classified according to WHO guidelines.[30] The Warwick-Edinburgh Mental Well-being Scale,[31] and the Hospital Anxiety and Depression Scale[32] were administered at each time-point. Attendance at Momenta and leisure centre usage was monitored via swipe-card tracking. 52 weeks after commencing the programme, participants were invited to attend a follow-up session, where leisure staff repeated physiological and psychological measures. Programme providers collected and collated quantitative data and provided an anonymised dataset to the evaluation team for analysis.

Qualitative evaluation component

Implementation effectiveness for the referral process was explored through semi-structured interviews with referring HCPs (undertaken at referring surgeries) and focus groups with service users (in leisure centres). All were conducted by LN during March-July 2015, as part

For peer review only - http://bmjopen.bmj.com/site/about/guidelines.xhtml

of her Public Health Masters degree (which contained qualitative methods training), mentored by TF, an experienced qualitative researcher. Questions are included in supplementary file 1. Data were audio-recorded. Results are reported using the Consolidated criteria for Reporting Qualitative research guidelines[33]

Practice managers from all six referring surgeries were sent an invitation for staff to take part (n = 84), (General Practitioner = 53, Practice Nurse = 18, Health Care Assistant = 13). Individual correspondence was sent to those agreeing. Interviews aimed to explore HCPs' referral experiences; raising weight issues; assessing readiness to change; marketing and referral materials; and the referral process. Interview questions were pilot tested with public health colleagues to assess timing and ensure validity. One question (*Thinking about after you referred the patient, what happened next?*) was omitted after piloting as it was realised HCPs would not have had patient feedback at that point. Interviews lasted on average 26 minutes and were transcribed verbatim. Data were analysed following each interview, with developing themes considered to determine whether questions required refinement. Initial themes generated from the first two interviews did not change and thus questions remained constant, although prompts were added.

During the initial assessment session for the first wave of referrals, all (n = 39) were invited to participate in a series of focus groups at programme-end to explore experiences. Emphasis was placed on the referral process, initial expectations and experiences of participation; how weight issues were raised by HCPs; time from referral to initial assessment; and facilitators and barriers to taking part. Focus groups lasted between 26 and 44 minutes.

Patient and public involvement

For peer review only - http://bmjopen.bmj.com/site/about/guidelines.xhtml

Data from deliverers and service users, along with direct input from commissioners, fed into the iterative evaluation.

Data analyses

The anonymised quantitative dataset was analysed using PSAW Statistics V.22. Descriptive statistics were calculated for age, gender, IMD, employment status, initial BMI, leisure site, level of education, and uptake and adherence. Distribution and normality of measures (weight, BMI, waist circumference, psychological wellbeing and attendance) were assessed using Shapiro Wilk tests and median and interquartile range (IQR) scores calculated for each group at baseline and 12 weeks (attendance, 12 weeks only). Using complete cases, Kruskal-Wallis H tests were used to determine between-group differences at baseline and at 12 weeks and Wilcoxon-signed rank tests examined repeated measures differences between baseline and 12-week scores. Complete cases available at 52 weeks (n = 37) were considered similarly, but via separate analyses due to limited available data across the comparison groups.

Qualitative data were audio-recorded and transcribed by LN using a thematic process.[34] Data were organised according to concepts, key themes and developing categories. Data coding was discussed with TF, allowing comparison of data interpretation and subsequent coding refinement. Evolving key themes were refined through the analysis process and subsequent cross-sectional thematic labelling of data, thus generating deeper understanding. Where possible, key phrases or expressions identified from interviews and focus groups were retained within coding and thematic labelling. A public health colleague helped to verify

For peer review only - http://bmjopen.bmj.com/site/about/guidelines.xhtml

interpretations of the data and appropriateness of codes applied. Once initial interviews were coded this framework was applied to remaining data. Notes taken during focus groups helped to contextualise when developing themes and included information about dynamics within groups, such as influence, disagreement, humour and peer exposure.

RESULTS

Between December 2014 and March 2016, the programme received 182 referrals and was delivered in four cohorts across leisure sites. Due to initial low levels of recruitment, the first cohort did not start until March 2015. Referrals were mainly female (83%) and 30.6% lived in the 20% most deprived areas (table 1).

For peer review only

For peer review only - http://bmjopen.bmj.com/site/about/guidelines.xhtml

Table 1. Demographic characteristics of referrals (n=165)

	Median	IQR
**Age (years)**	53	24
**Gender (n=167)**	**n**	**%**
Male	29	17.4%
Female	138	82.6%
**Initial BMI category (kg/m^2, n=150)**		
25.0-29.9	40	26.7%
30.0-34.9	73	48.6%
35.0-39.9	27	18.0%
≥40.0	10	6.7%
**Leisure site (n=170)**		
Leisure site 1 (IMD quintile 2)	80	44.7%
Leisure site 2 (IMD quintile 3)	99	55.3%
**Index of multiple deprivation (n=170)**		
20% most deprived	52	30.6%
21-40%	41	24.1%
41-60%	20	11.8%
61-80%	20	11.8%
20% least deprived	37	21.6%
**Employment status (n=123)**		
Employed full time	37	30.1%
Employed part time	24	19.5%
Retired	51	41.5%
Claiming incapacity benefit	5	4.1%
Claiming job seekers allowance	6	4.9%
**Level of education (n=127)**		
Primary	15	11.8%
Secondary (O level/GCSE)	35	27.6%
Secondary (A level)	26	20.5%
Further education (HND)	25	19.7%
Bachelors or equivalent	21	16.5%
Masters or equivalent	5	3.9%

Preliminary programme effectiveness

Of all referrals, 153 (84%) attended the baseline measurement session and 78 (51% of those who started) attended the 12-week measurement session. Uptake and adherence varied by programme group (table 2).

For peer review only - http://bmjopen.bmj.com/site/about/guidelines.xhtml
For peer review only

Table 2. Programme uptake, adherence and attendance.

Uptake and adherence	Momenta-Fitness		Momenta only		Fitness only	
Number referred		58		59		65
Uptake* (n, %)		54 (93.1%)		50 (84.7%)		49 (75.4%)
Uptake retention** (n, %)		35 (64.8%)		27 (54.0%)		16 (32.7%)
Uptake adherence^ (n, %)		34 (63.0%)		26 (52.0%)		8 (50.0%)
Overall retention*** (n, %)		35 (60.3%)		27 (45.8%)		16 (24.6%)
Overall adherence^^ (n, %)		34 (58.6%)		26 (44.1%)		8 (12.3%)
**Momenta session attendance**	**Momenta-Fitness**		**Momenta only**		**Fitness only**	
	**n**	**Median (IQR)**	**n**	**Median (IQR)**		
Uptake	54	9.0 (7.3)	50	9.0 (8.0)		
Dropouts	19	3.0 (3.0)	23	3.0 (5.0)		N/A
Completers^^^	35	10.0 (2.0)	27	11.0 (1.3)		
**Exercise session attendance**	**Momenta-Fitness**		**Momenta only**		**Fitness only**	
	**n**	**Median (IQR)**	**n**	**Median (IQR)**	**n**	**Median (IQR)**
Uptake	54	7.0 (16.3)	50	0.0 (4.5)	49	0.0 (1.5)
Dropouts	19	0.0 (1.0)	23	0.0 (0.0)	33	0.0 (0.0)
Completers^^^	35	10.0 (14.0)	26	0.0 (5.0)	16	4.5 (18.0)

Uptake* *participant attended baseline assessment;* ***Uptake retention***** *% of participants who attended the baseline assessment who also attended the 12-week assessment;* ***Uptake adherence**^ *% of participants who attended the baseline assessment who also attended ≥ 8 Momenta sessions (Momenta-Fitness and Momenta only) or gym sessions (fitness only);* ***Overall retention****** *% of all those referred who attended both baseline and 12-week assessment;* ***Overall adherence**^^ *% of all those referred who attended ≥ eight Momenta sessions (Momenta-Fitness and Momenta only) or exercise sessions (fitness only); Completers^^^ those who completed the 12-week assessment*

Physiological and psychological data were not normally distributed. Quantitative findings are presented as exploratory, due to the small sample size. No significant differences were found between programme groups either at baseline or at 12 weeks, for any measures. Significant within-group differences between baseline and 12 weeks were evident for weight, BMI and waist circumference for Momenta-Fitness, and Momenta (Table 3). Follow-up analysis at 52-weeks (available sub-sample) showed changes were maintained for Momenta-Fitness (*n* =18) only.

For peer review only - http://bmjopen.bmj.com/site/about/guidelines.xhtml

Table 3. Weight, BMI and waist circumference change.

End of programme results	Median (IQR) Baseline	Median (IQR) 12 weeks	z	p	Median (IQR) Change
**Weight (kg)**					
Momenta-Fitness (n=35)	88.9 (80.5 - 100.0)	88.0 (77.2 - 95.8)	-4.531	<0.001	-2.9 (-5.1 - -1.6)
Momenta only (n=26)	87.8 (74.5 - 77.0)	83.3 (74.5 - 92.5)	-4.344	<0.001	-2.9 (-5.0 - -2.0)
Fitness only (n=15)	76.2 (71.6 - 86.9)	76.6 (70.4 - 84.6)	-0.879	0.379	0.0 (-3.2 - 1.0)
**BMI (kg/m²)**					
Momenta-Fitness (n=35)	32.0 (30.3 - 35.7)	31.3 (29.2 – 35.3)	-4-494	<0.001	-1.1 (-1.9 - -0.6)
Momenta only (n=26)	32.0 (30.0 - 34.5)	31.3 (28.6 – 33.6)	-4.356	<0.001	-1.2 (-1.6 - -0.8)
Fitness only (n=14)	29.2 (27.3 - 33.0)	29.7 (27.0 – 33.3)	-0.454	0.650	0.1 (-1.2 - +0.4)
**Waist circumference (cm)**					
Momenta-fitness (n=35)	106.0 (98.0 - 115.0)	99.0 (93.0 - 110.0)	-4.996	<0.001	-7.0 (-9.5 - -5.0)
Momenta only (n=25)	108.0 (99.5 - 114.5)	101.0 (93.8 - 111.5)	-4.166	<0.001	-5.0 (-7.3 - -2.5)
Fitness only (n=11)	90.0 (87.0 – 95.0)	91.0 (90.0 - 96.0)	0.358	0.650	1.0 (-3.0 - 3.0)
**52-week follow-up**	**Median (IQR) Baseline**	**Median (IQR) 52 weeks**	**z**	**p**	**Median (IQR) Change**
**Weight (kg)**					
Momenta-Fitness (n=18)	95.2 (87.1 - 101.4)	91.4 (82.7 - 95.9)	-3.006	<0.001	-4.8 (-6.2 - -1.5)
Momenta only (n=16)	84.7 (72.3 - 95.2)	82.7 (73.2 - 94.6)	-1.533	0.120	-0.7 (-7.6 - 0.8)
*Fitness only (n=3)	73.4 (69.5 - 80.2)	70.3 (66.0 - 87.0)			0.9 (-7.4 – 6.9)
**BMI (kg/m²)**					
Momenta-Fitness (n=18)	32.0 (30.49 - 35.1)	30.8 (28.7 - 34.0)	-3.157	<0.05	-1.7 (-2.0 - -0.6)
Momenta only (n=16)	31.7 (29.3 – 33.9)	31.1 (26.7 – 33.6)	-1.603	0.109	-0.3 (-2.3 - 0.3)
*Fitness only (n=3)	27.6 (27.5 - 30.5)	27.8 (24.8 – 33.2)			0.3 (24.8 - 33.2)
**Waist circumference (cm)**					
Momenta-Fitness (n=18)	109.0 (101.0 - 114.8)	100.5 (94.8 - 107.3)	-3.221	<0.001	-6.0 (-13.3 - -1.75)
Momenta only (n=16)	106.0 (94.5 - 115.8)	103.5 (98.5 - 113.3)	-0.780	0.938	-2.5 (-9.0 - -10.0)
*Fitness only (n=3)	89.0 (87.0 – 95.0)	90.0 (90.0 – 101.0)			3.0 (90.0 – 101.0)

** Fitness only n=3 therefore median and range reported and no statistical test completed.*

Significant improvement in mental wellbeing, and reductions in depression and anxiety, were evident between baseline and 12 weeks for Momenta-Fitness, and Momenta (Table 4). The magnitude of change was relatively small but functionally and clinically meaningful. For example, the median value for anxiety for Momenta dropped from a moderate symptomology to a not symptomatic classification. 52-week sub-sample analysis showed that significant improvements for wellbeing and depression were maintained for Momenta-Fitness (n=18), and wellbeing and anxiety for Momenta (n=16).

For peer review only

For peer review only - http://bmjopen.bmj.com/site/about/guidelines.xhtml

Table 4. Wellbeing, anxiety and depression measures change.

End of programme results	Median (IQR) Baseline	Median (IQR) 12 weeks	z	p	Median (IQR) Change
**Mental wellbeing scale**					
Momenta-Fitness (n=29)	46.0 (40.0 - 51.5)	53.0 (40.0 - 51.5)	3.810	<0.001	5.0 (1.5 - 12.0)
Momenta only (n=23)	49.0 (39.0 - 58.0)	55.0 (51.0 - 63.0)	2.818	<0.05	6.0 (-1.0 - 10.0)
Fitness only (n=13)	47.0 (40.5 - 59.5)	46.0 (42.0 - 63.5)	0.157	0.875	0.0 (-4.0 - 5.0)
**Anxiety scale**					
Momenta-Fitness (n=28)	5.5 (4.0 - 9.8)	4.5 (2.0 - 7.0)	-3.027	<0.001	-1.0 (-3.0 - 1.0)
Momenta only (n=23)	8.0 (6.0 - 10.0)	4.0 (2.5 - 9.0)	-2.329	<0.05	-1.0 (-3.0 - 0.0)
Fitness only (n=13)	8.0 (3.5 - 10.0)	6.0 (4.0 - 9.0)	-0.499	0.618	-1.0 (-2.0 - 2.0)
**Depression scale**					
Momenta-Fitness (n=28)	5.5 (3.3 - 8.0)	2.0 (1.0 - 6.0)	-3.214	<0.05	-2.5 (-4.8 - -0.3)
Momenta only (n=23)	5.0 (3.0 - 7.5)	3.0 (1.0 - 5.0)	-3.049	<0.05	-1.0 (-4.5 - 1.0)
Fitness only (n=13)	4.0 (2.0 - 8.5)	2.0 (2.0 - 7.0)	-1.226	0.220	-2.0 (-4.5 - 0.0)

52-week follow-up	Median (IQR) Baseline	Median (IQR) 52 weeks	z	p	Median (IQR) Change
**Mental wellbeing scale**					
Momenta-Fitness (n=15)	44.0 (39.0 - 52.0)	55.0 (48.0 - 59.0)	2.984	<0.05	5.0 (3.0 - 15.0)
Momenta only (n=13)	58.0 (47.5 - 59.0)	56.0 (54.0 - 63.5)	2.282	<0.05	4.0 (0.5 - 6.5)
*Fitness only (n=3)	47.0 (34.0 – 64.0)	58.0 (45.0 – 60.0)			-2.0 (-6.0 – 26.0)
**Anxiety scale**					
Momenta-Fitness (n=15)	6.0 (2.0 - 10.0)	2.0 (1.0 - 7.0)	-1.785	0.074	-3.0 (-6.0 - 0.0)
Momenta only (n=15)	7.0 (4.0 - 9.0)	5.0 (1.0 - 8.0)	-1.990	<0.05	-3.0 (-4.0 - 0.0)
*Fitness only (n=3)	9.0 (5.0 – 10.0)	2.0 (1.0 – 8.0)			-3.0 (-8.00 - -2.0)
**Depression scale**					
Momenta-Fitness (n=15)	7.0 (3.3 - 11.3)	3.5 (1.0 - 6.0)	-2.908	<0.05	-3.5 (-6.3 - -0.8)
Momenta only (n=15)	4.0 (1.0 - 6.0)	3.0 (1.0 - 4.0)	-0.762	0.446	0.0 (-2.0 - 1.0)
*Fitness only (n=3)	3.0 (0.0 – 8.0)	2.0 (1.0 – 8.0)			1.0 (-8.0 – 5.0)

** Fitness only n=3 therefore median and range reported and no statistical test completed.*

Overall, the results suggested those who participated in the two groups incorporating Momenta, had enhanced physical and psychological health indicators from baseline, whereas those who had only free fitness membership did not. There is some evidence, for a small follow-up sample, that the combination of Momenta and fitness membership produces the best outcomes at 52 weeks.

For peer review only - http://bmjopen.bmj.com/site/about/guidelines.xhtml

Implementation effectiveness: reflections from referring healthcare professionals

Five face-to-face semi-structured interviews took place with HCPs across five referring surgeries: two GPs, two Practice Nurses and one Health Care Assistant. HCPs perceived that four key themes influenced the effectiveness of programme implementation: (i) difficulties raising weight with patients, (ii) how gender affected patient engagement, (iii) availability of information and resources, and (iv) additional barriers constraining programme promotion.

Raising the issue of weight with patients:

Concerns about raising weight may have contributed to slow recruitment, with nurses and healthcare assistants expressing unease, *'not really up to me… well I talk about it if they want to…. Better if they [patients] bring it up.'* (Interview 2, Healthcare Assistant). GPs seemed more comfortable raising weight with patients, but with the caveat that this is easier in the context of a longer-term GP/patient relationship.

> *'the people I see I've known for a very long time… it's the rapport you have…if I'd never met anyone before and they came in for a sore throat I'm not going to say you're fat…If there was someone I'd known for a long time and it seemed relevant…I'd mention it.'* (Interview 5, GP).

Gender and engagement in the referral process:

Gender was highlighted as influencing the referral process, women being more likely than men to seek support for weight. This may help explain the low rate of referral for males (17%):

For peer review only

'More women talk about it…men don't really talk about weight…I do mention weight to men if I'm doing a well man [sic] but it doesn't come up really…it's a woman thing…' (Interview 3, Practice Nurse).

Availability of information and resources:

Several interviewees highlighted training needs around programme information and resources, (e.g., additional programme information would help to engage patients). For example, the GPs both discussed the longstanding ERS and stated they needed to become more familiar with Momenta, as they had with the ERS:

'when we get opportunities to do things in the practice we normally discuss it, let everyone know where appropriate forms and information is and it's in your head…that didn't happen with this and I don't know why that was.' (Interview 5, GP).

All HCPs interviewed felt the referral leaflet (provided by programme providers) was important in the process, either as a tool to promote the intervention or to convey information to patients:

'The leaflet was good, bright…explained the programme and patients like taking a leaflet away.' (Interview 3, Practice Nurse)

Additional Barriers to Engagement:

For peer review only - http://bmjopen.bmj.com/site/about/guidelines.xhtml

Several sub-themes highlighted additional barriers to the referral process. The most prominent sub-themes were around initial BMI referral criteria (25.0-29.9 kg/m^2) and delayed programme start. Both implementation factors were beyond the control of the referrers, but consequently amended through iterative refinement during the prototyping process following early data analysis. Both were reported by practice nurses as exacerbating each other:

> '*we were referring but then it didn't start so people were not sure what was happening [pause]…Think it was more people were needed to start…but you know if the BMI was higher then there would have been more*.' (Interview 3, Practice Nurse).

In one case, a decision was taken to relax the referral criteria, *'…31.5 [kg/m^2]…was just outside so I just referred him.*' (Interview 4, GP).

Programme location was perceived by HCPs to overcome an existing barrier to the tier three weight management programme, as Momenta was '*round the corner for people,*' as opposed to '*a bit far away at the hospital.'* Cost barriers were also discussed, both with reference to the patient, *'in this sort of area…cost…, if you've got to pay it's a barrier.'* (Interview 4, GP), and to expected targets from Clinical Commissioning Groups (CCG),

> *'we are constantly told by the CCG that we must keep down on numbers and that if there are costs attached to this referral that would definitely impact… and that would be for all practices.'* (Interview 5, GP)

Implementation effectiveness: reflections from participants

Three focus groups allowed programme participant voices to be heard: three females and one male from Momenta (focus group 1), three males and three females from Momenta-Fitness

For peer review only - http://bmjopen.bmj.com/site/about/guidelines.xhtml

(focus group 2) and three females (one of whom emailed her views separately) from Fitness-only. Across the groups, 12 participants reported having lost weight and one reported weight gain. Three themes developed: (i) outcomes of the programme, (ii) facilitators and barriers to engagement, and (iii) raising the issues of weight with HCPs.

Outcomes of the programme:

Focus group findings aligned closely with quantitative outcomes in terms of the physical and psychological benefits of participation: *'[I've] lost a good bit of weight. It's been very positive for me… I'm feeling a lot more active…'* (Momenta-Fitness, Participant 5). Participants reported a sense of weight loss achievement, increased physical activity levels, and positive mood states. In addition, elements of the Momenta programme were perceived as facilitating engagement, including the *'group feeling… I looked forward to it,'* (Momenta-Fitness, Participant 4), the *'information that we got every week… so very well planned.'* (Momenta-Fitness, Participant 3) and the ongoing support e.g., *'she 'phoned me the other day to see if I was coming,'* (Momenta-Fitness, Participant 4). Momenta participants reflected back on, and identified and discussed lifestyle factors that related to their initial weight gain (e.g., *'I did the usual thing… I started eating toffees,'* Momenta-Fitness, Participant 5*),* demonstrating both self-awareness and an openness to discussing the topic.

Facilitators and barriers to engagement:

One participant reported being initially excluded but later allowed to take part, and others raised concerns that the initial BMI threshold for referral (25-29.9 kg/m^2) was too low, '*was a little bit high, BMI…managed to get it down... [and then] the doctor put us forward,'*

(Momenta, Participant 2). Data also indicated the importance of subsidised access, particularly important in the context of a deprived region such as this, e.g., '*I also joined Weight Watchers for short period of time but found the classes too expensive,*' (Fitness-only, Participant 3, emailed response).

Raising the issue of weight with HCPs:

Some data did suggest implementation was problematic, however, this focused exclusively on the referral process. Participants overwhelmingly felt that they had opened the conversation about weight, as opposed to discussions being initiated by HCPs (e.g., '*my glucose levels were quite high but nobody ever said that I was overweight*,' Momenta-Fitness, Participant 4*)*. In addition, participants perceived limitations in HCPs' knowledge of intervention components ('*she [nurse] didn't know anything about it*,' Fitness-only, Participant 1), something with potential to impact on likelihood of referral, and participants' expectations of programme success.

Iterative refinements throughout the evaluation process

Here we list a number of implementation adjustments which were made throughout the evaluation process, facilitated via the prototyping framework. Real-time advice from Commissioners was considered during early stages of implementation, regarding the nature of comparison offers (e.g. fitness access) and thus initial design and outcome measurements were adapted prior to referrals being made. To better-target recruitment and change the process of engagement at referral point, entry criteria were altered (BMI $\geq$30 kg/m^2) mid-way through programme delivery. On-site implementation of the service offer was adapted in

For peer review only - http://bmjopen.bmj.com/site/about/guidelines.xhtml

response to delivery staff feedback: increased resource was made available, for example additional staffing to support delivery for the first wave of referrals. Furthermore, staff were given additional time for Momenta session preparation and session delivery times were extended. Follow-up activities (i.e., text or telephone contact) were implemented by staff during the process, to encourage adherence.

DISCUSSION

We explored 'prototyping', as a cost-effective and time-efficient approach to public health evaluation, via an 'off-the-shelf' weight management programme implemented in a local context of mixed and high deprivation. Quantitative findings should be interpreted as exploratory, due to the relatively small number of complete cases, however lessons can be learned from these data both in terms of preliminary outcomes and engagement/dropout. Participation in Momenta and Momenta-Fitness resulted in 12-week weight loss for those who completed the programme. Free fitness membership without the weight-management programme was poorly engaged with and did not lead to weight change. A small sub-sample who attended follow-up demonstrated that after one year, weight reductions equivalent to ~4% could be maintained for Momenta-Fitness. We caution that this might be best interpreted as hypothesis-generating for future evaluations, given the small number of available cases, however we will consider potential mechanisms here. Providing free access to fitness facilities alongside the behaviour change programme may allow for continuous and self-driven behaviour change[35] and sustaining optimal changes in adiposity over 12 months in those who remained engaged.[36] Swipe card monitoring during the initial 12-week period indicated that fitness sessions were accessed an average 10 occasions for this group, whereas no access was apparent for Momenta, despite Momenta sessions being held in leisure centres.

For peer review only - http://bmjopen.bmj.com/site/about/guidelines.xhtml

This could be important for community providers making decisions about delivery location. Both Momenta groups reported improved wellbeing, and reduced anxiety and depression at 12-weeks suggesting that the behavioural intervention may drive this effect. This is consistent with previous work reporting co-varying changes in weight loss, depression, and quality of life in weight management services.[37] It is unclear whether the primary mechanism was weight loss, or the wider social benefits of participation. Both were valued in the qualitative data. Our preliminary evidence of maintained improvements in wellbeing for these groups at 52 weeks is particularly relevant given previously evidenced associations between poor mental health, and obesity and overweight status.[38]

Experiences of both referrers and referrals highlighted that HCPs needed to be better-informed and more confident raising weight-related conversations. Whilst patient-led action is desirable, staff reluctance to raise weight issues may mean that opportunities for engagement of less knowledgeable or motivated patients will be missed. The problematic positioning of GPs within obesity care has been highlighted previously,[39] with a range of strategies to change HCPs' behaviour resulting in little or no change to patients' weight. A practical training need is highlighted for those working at the patient-practitioner interface, however communication with patients about weight may well be hindered by the 'stigma' attached to obesity.[40] This has wider implications for patient outcomes and requires further exploration through the implementation process. Additionally, HPCs need better understanding of referral-based public health programmes offered. Despite efforts of programme and public health managers, awareness was reportedly low for some referring professionals. We suggest consideration of resource-efficient ways to signpost both HPCs and patients themselves as part of the implementation process.

For peer review only - http://bmjopen.bmj.com/site/about/guidelines.xhtml

This programme was delivered across a social gradient in a region with low health indices and areas of high deprivation. Some issues in relation to inequalities and service access for future community-based weight management programmes were highlighted. Only 17% of referrals to Momenta were males. Gender bias in weight management referral has been reported elsewhere,[41-42] and interviews showed that practitioners struggled to raise the topic of weight with male patients. Alternative referral strategies have been employed in other settings in an attempt to overcome this.[43] Marketing in other community spaces, or targeted postal referrals could be explored in future implementation. The initial decision to restrict referral to overweight-only substantially impacted on referral rates, with HCPs and referrals indicating they felt limited until this restriction was reversed. Had this continued, worsening health inequalities may have been an unintended consequence, something to be actively avoided within public health programmes [44.] The roles of, and interactions between, those operating in the 'system' (i.e. the context within which the intervention operates) must be considered at the point of implementation to minimise any impact from unintended consequences.[5] In practical terms, this may be through continued dialogue with commissioners, referring professionals and referrals themselves, something which prototyping evaluation allows.

Given that no systematic problems emerged with participants' experiences of the programme itself, our findings lend support to a streamlined approach to involvement of all stakeholders in programme implementation. We suggest that prototyping demonstrates opportunities for off-the-shelf programmes to be pragmatically moulded to local context, in real-time. Many of the iterative changes made were staff-driven. This demonstrates that real-time consideration of feedback from on-site delivery teams can be important to the implementation process. Some of the adjustments required commissioning action, as they had resource implications;

For peer review only - http://bmjopen.bmj.com/site/about/guidelines.xhtml

others needed advice from the evaluation team. Interestingly changes made throughout the process generally focused on both staff and participant experience.

Emergence of some negative experiences of referral suggests, however, that prototyping can be problematic without networks or channels for ensuring key outcomes are widely communicated to relevant stakeholders. Overall, the evaluation demonstrated that a balance is needed to allow quick and efficient adaptation of off-the-shelf programmes, but with focused professional user engagement in the early stages of development. The prototyping approach had particular utility given that project resources were limited and meant that issues were identified and acted upon rapidly. While the programme may have progressed similarly without this, prototyping provided a greater structure for, and confidence in, on-going refinements. This was achieved via the support provided by academics, public health practitioners and providers.   Fundamentally, adopting a prototyping approach enabled the delivery of a new service to an in-need population, alongside the generation of initial evidence of local effectiveness. A minimum of 1 kg weight-loss at 3 months, and 0.7 kg at 12-months have been suggested as thresholds to influence decisions over commissioning of weight-loss services.[45] Our preliminary data shows that Momenta met and indeed exceeded these and shows particular promise when implemented in conjunction with free fitness provision.

Demonstrating preliminary effectiveness is of limited use, however, unless a successful programme in one area may be adapted and implemented to suit a different context, for example through sharing local-level knowledge, interactions and behaviours of individuals within different parts of that system.[46] The process for scaling-up of effective health interventions to broader policy and practice takes years[47] and certainly within the obesity

For peer review only - http://bmjopen.bmj.com/site/about/guidelines.xhtml

literature, has been dominated by initiatives that consider effectiveness but not implementation across specific settings.[48-49] We recommend prototyping might be built into larger public health evaluations providing that the original programme has a sound theoretical basis, and iterative refinement is engaged with by all stakeholders from the outset.

CONCLUSION

We demonstrate good preliminary outcome effectiveness for 'Momenta', particularly in conjunction with a free fitness offer. The programme was experienced positively by those who attended. Issues with the referral process need to be explored further, however other refinements were feasible during delivery. This evaluation extends the literature by exploring prototyping for a complex problem, community weight-management, in a challenging setting, demonstrating streamlined implementation of an 'off-the-shelf' weight management programme. This resource-effective approach is highly relevant in the context of health inequalities and public health sector funding constraints.

Acknowledgements: Many thanks to Jordan Bell, Nicole Rowley and Ross Davison, who collected and collated raw quantitative data.

Author Contributions: CDR, CH and EO contributed to design of the quantitative evaluation, data analysis and preparation the final document. LN contributed to design and analyses of the qualitative evaluation component. TF contributed to qualitative design and analysis and preparation of the final document. AL contributed to design of the quantitative evaluation and preparation of the final document.

For peer review only - http://bmjopen.bmj.com/site/about/guidelines.xhtml

Funding: The quantitative evaluation component was supported by funding from the Wolfson Research Institute for Health and Wellbeing, Durham University. Amelia Lake is a member of Fuse, the Centre for Translational Research in Public Health (www.fuse.ac.uk). Fuse is a UK Clinical Research Collaboration (UKCRC) Public Health Research Centre of Excellence. Funding for Fuse from the British Heart Foundation, Cancer Research UK, Economic and Social Research Council, Medical Research Council, the National Institute for Health Research, under the auspices of the UKCRC, is gratefully acknowledged. The views expressed in this paper do not necessarily represent those of the funders or UKCRC. The funders had no role in study design, data collection and analysis, decision to publish, or preparation of the manuscript.

Competing interests: CH is a former employee of the Leisure Trust and was instrumental in initiating the evaluation. She was subsequently employed as a research assistant at Durham University, however was not involved in any data collection or entry, only accessing an anonymised database submitted to the University. LN was a Public Health Improvement Manager within the Public Health Team and had responsibility for commissioning the Momenta programme. The qualitative evaluation component was submitted in partial fulfilment of her Masters in Public Health at Newcastle University.

Participant consent: Consent was obtained for face-to-face interviews and focus groups. Service users were informed in writing of the nature of the quantitative service evaluation and how to withdraw from it. The presented data are anonymised with risk of identification low.

For peer review only

For peer review only - http://bmjopen.bmj.com/site/about/guidelines.xhtml

Ethics approval: Ethical advice was sought from the local Research Manager of North of England Commissioning Support, and this project was classed as a service evaluation in line with National Research Ethics Service guidance.

Data availability statement: No additional data are available because this was a service evaluation.

For peer review only

For peer review only - http://bmjopen.bmj.com/site/about/guidelines.xhtml

REFERENCES

1. Haines A, Kuruvilla S, Borchert M. Bridging the implementation gap between knowledge and action for health. (Theme Papers). *Bull World Health Organ* 2004;82:724.

2. Curran GM, Bauer M, Mittman B, *et al*. Effectiveness-implementation Hybrid Designs: Combining Elements of Clinical Effectiveness and Implementation Research to Enhance Public Health Impact. *Med Care* 2012;50:217-26.

3. Sales A, Smith J, Curran G, *et al*. Models, Strategies, and Tools: Theory in Implementing Evidence-Based Findings into Health Care Practice. *J Gen Int Med* 2006;21:S43-S9.

4. Kilbourne AM, Neumann MS, Pincus HA, *et al*. Implementing evidence-based interventions in health care: application of the replicating effective programs framework. *Implement Sci* 2007;2:42.

5. Medical Research Council. Developing and evaluating complex interventions: new guidance. UK: Medical Research Council, 2006. https://mrc.ukri.org/documents/pdf/developing-and-evaluating-complex-interventions/. (Accessed 10 Aug 2018)

6. Oliver K, Kothari A, Mays N. The dark side of coproduction: do the costs outweight the benefits for health research? *Health Research Policy and Systems* 2019;17:33.

For peer review only - http://bmjopen.bmj.com/site/about/guidelines.xhtml

7. Meisinger C, Döring A, Thorand B, *et al*. Body fat distribution and risk of type 2 diabetes in the general population: are there differences between men and women? The MONICA/KORA Augsburg Cohort Study. *Am J Clin Nutr* 2006;84:483-9.

8. Bhaskaran K, Douglas I, Forbes H, *et al*. Body-mass index and risk of 22 specific cancers: a population-based cohort study of 5·24 million UK adults. *Lancet* 2014;384:755-65.

9. Zhang C, Rexrode KM, Van Dam RM, *et al*. Abdominal obesity and the risk of all-cause, cardiovascular, and cancer mortality: sixteen years of follow-up in US women. *Circ* 2008;117:1658-67.

10. Kolotkin RL, Andersen JR. A systematic review of reviews: exploring the relationship between obesity, weight loss and health-related quality of life. *Clin Obes* 2017;7:273-89.

11. Simon GE, Von Korff M, Saunders K, *et al*. Association between obesity and psychiatric disorders in the us adult population. *Arch Gen Psychiatry* 2006;63(7):824-30.

12. Public Health England. *Health matters: obesity and the food environment environment.* UK: Public Health England, 2017. https://www.gov.uk/government/publications/health-matters-obesity-and-the-food-environment/health-matters-obesity-and-the-food-environment--2. (Accessed 10 Aug 2018)

For peer review only

13. World Health Organisation. *Global status report on noncommunicable diseases 2014.* Geneva: World Health Organisation, 2014. https://apps.who.int/iris/bitstream/handle/10665/148114/9789241564854_eng.pdf;jsessionid=A043D0E63AACA67752CC3294A1EBD7A3?sequence=1. (Accessed 04 Feb 2019)

14. Bambra C, Hillier F, Cairns J, *et al*. How effective are interventions at reducing socioeconomic inequalities in obesity among children and adults? Two systematic reviews. *Public Health Res* 2015;3.

15. Bull ER, Dombrowski SU, McCleary N, *et al*. Are interventions for low-income groups effective in changing healthy eating, physical activity and smoking behaviours? A systematic review and meta-analysis. *BMJ Open*. 2014;4:e006046.

16. Office for National Statistics. *Dataset: Regional gross value added (balanced) by combined authority in the UK.* UK: Office for National Statistics, 2017. https://www.ons.gov.uk/economy/grossvalueaddedgva/datasets/regionalgrossvalueaddedbalancedbycombinedauthorityintheuk. (Accessed 10 Aug 2018)

17. Public Health England. *Work, worklessness and health: local infographic tool*. UK: Public Health England, 2018. https://www.gov.uk/government/publications/work-worklessness-and-health-local-infographic-tool. (Accessed 10 Aug 2018)

18. Northumberland County Council. *Public Health Service Statement 2017/18.* Northumberland: Northumberland County Council, 2017. https://northumberland.gov.uk/NorthumberlandCountyCouncil/media/About-the-

For peer review only

Council/Performance/Service%20Statements%202016/2017-Public-Health-Service-Statement-2017-18.pdf. (Accessed 10 Aug 2018)

19. Public Health England. *Local Authority Health Profiles*. UK: Public Health England, 2019. https://fingertips.phe.org.uk/profile/health-profiles/data#page/0/gid/1938132701/pat/6/par/E12000001/ati/102/are/E06000057/iid/10101/age/169/sex/4. (Accessed 01 Feb 2019)

20. Lister C, Payne H, Hanson CL, *et al*. The Public Health Innovation Model: Merging Private Sector Processes with Public Health Strengths. *Front Public Health* 2017;5:192.

21. Parry GJ, Carson-Stevens A, Luff DF, *et al*. Recommendations for evaluation of health care improvement initiatives. *Acad Pediatr* 2013 Nov-Dec;13(6 Suppl):S23-30.

22. Hawkins J, Madden K, Fletcher A, *et al*. Development of a framework for the co-production and prototyping of public health interventions. *BMC Public Health* 2017;17:689.

23. Stevens VJ, Funk KL, Brantley PJ, *et al*. Design and implementation of an interactive website to support long-term maintenance of weight loss. *J Med Internet Res* 2008;10(1):e1.

24. Discover Momenta Ltd. *Momenta.* UK: Discover Momenta Ltd, 2013: http://discovermomenta.com/. (Accessed 10 Aug 2018)

For peer review only - http://bmjopen.bmj.com/site/about/guidelines.xhtml

25. Hanson CL. *Advancing understanding of effective exercise on referral: a mixed methods evaluation of the Northumberland scheme*. PhD Thesis, Durham University, 2017. http://etheses.dur.ac.uk/12162/. (Accessed 10 Aug 2018)

26. Hanson CL, Allin LJ, Ellis JG, *et al*. An evaluation of the efficacy of the exercise on referral scheme in Northumberland, UK: association with physical activity and predictors of engagement. A naturalistic observation study. *BMJ Open* 2013;3:e002849

27. Sacher PM, Kolotourou M, Chadwick PM, *et al*. Randomized Controlled Trial of the MEND Program: A Family-based Community Intervention for Childhood Obesity. *Obes* 2012;18:S62-S8.

28. Leech N, Onwuegbuzie A. A typology of mixed methods research designs. *Qual Quant* 2009;43:265-75.

29. Marfell-Jones M, Olds T, Stewart A, *et al*. *International Standards for Anthropometric Assessment (2007)*. Potchefstroom, South Africa: International Society for the Advancement of Kinathropometry, 2007.

30. World Health Organisation. *BMI Classification 2006.* Geneva: World Health Organisation, 2006. http://apps.who.int/bmi/index.jsp?introPage=intro_3.html. (Accessed 10 Aug 2018)

31. Clarke A, Friede T, Putz R, *et al*. Warwick-Edinburgh Mental Well-being Scale (WEMWBS): Validated for teenage school students in England and Scotland. A mixed methods assessment. *BMC Public Health* 2011;11:487.

For peer review only - http://bmjopen.bmj.com/site/about/guidelines.xhtml

32. Zigmond AS, Snaith RP. The Hospital Anxiety and Depression Scale. *Acta Psychiatr Scand* 1983;67:361-70.

33. Tong A, Sainsbury P, Craig J. Consolidated criteria for reporting qualitative research (COREQ): a 32-item checklist for interviews and focus groups. *Int J Qual. Health Care* 2007;19:349–57

34. Braun V, Clarke V. Using thematic analysis in psychology. *Qual Res Psychol* 2006;3:77-101.

35. Mastellos N, Gunn LH, Felix LM, *et al*. Transtheoretical model stages of change for dietary and physical exercise modification in weight loss management for overweight and obese adults. *Cochrane Database Syst Rev* 2014;2:CD008066

36. Garrow JS, Summerbell CD. Meta-analysis: effect of exercise, with or without dieting, on the body composition of overweight subjects. *Eur J Clin Nutr* 1995;49:1-10.

37. Wright F, Boyle S, Baxter K, *et al*. Understanding the relationship between weight loss, emotional well-being and health-related quality of life in patients attending a specialist obesity weight management service. *J Health Psychol* 2013;18:574-86.

38. Somerville R, McKenzie K, Eslami S, *et al*. Poor mental health in severely obese patients is not explained by the presence of comorbidities. *Clin Obes* 2014;5:12-21.

39. Flodgren G, Goncalves BDC, Summerbell CD. Interventions to change the behaviour of health professionals and the organisation of care to promote weight reduction in children

For peer review only

For peer review only - http://bmjopen.bmj.com/site/about/guidelines.xhtml

and adults with overweight or obesity. *Cochrane Database of Syst Rev* 2017;11:CD000984.

40. Phelan SM, Burgess DJ, Yeazel MW, *et al*. Impact of weight bias and stigma on quality of care and outcomes for patients with obesity. *Obes Rev* 2015;16:319-26.

41. Logue J, Allardice G, Gillies M, *et al*. Outcomes of a specialist weight management programme in the UK National Health Service: prospective study of 1838 patients. *BMJ Open* 2014;4:e003747.

42. Lebrun LA, Chowdhury J, Sripipatana A, *et al*. Overweight/obesity and weight-related treatment among patients in U.S. federally supported health centers. *Obes Res Clin Pract* 2013;7:e377-e90.

43. Ahern AL, Aveyard P, Boyland EJ, *et al*. Inequalities in the uptake of weight management interventions in a pragmatic trial: an observational study in primary care. *Br J Gen Pract* 2016;66(645):e258-63.

44. Adams J, Mytton O, White M, *et al*. Why Are Some Population Interventions for Diet and Obesity More Equitable and Effective Than Others? The Role of Individual Agency. *PLoS Med* 2016;13(4).

45. Madigan CD, Daley AJ, Lewis AL, et al. Which weight-loss programmes are as effective as Weight Watchers®? Brit J Gen Pract 2014;64(620):e128-e136.

For peer review only

46. Curtis S, Oven K, Wistow J, *et al*. Adaptation to extreme weather events in complex health and social care systems: The example of older people's services in England. *Environ Plann C: Politics and Space* 2018;36:67-91.

47. Milat AJ, King L, Newson R, *et al*. Increasing the scale and adoption of population health interventions: experiences and perspectives of policy makers, practitioners, and researchers. *Health Res Policy and Systems* 2014;12:18.

48. Jebb S. *Dusting off Foresight's obesity report.* Foresight Blog UK: Government Office for Science, 2017. https://foresightprojects.blog.gov.uk/2017/10/04/dusting-off-foresights-obesity-report/. (Accessed 01 Feb 2019)

49. Wolfenden L, Goldman S, Stacey FG *et al*. Strategies to improve the implementation of workplace-based policies or practices targeting tobacco, alcohol, diet, physical activity and obesity. *Cochrane Database of Syst Rev* 2018;11:CD012439.

For peer review only

For peer review only - http://bmjopen.bmj.com/site/about/guidelines.xhtml

SUPPLEMENTARY FILE 1.

Healthcare professionals' semi-structured interview guide

Semi Structured Interview Set the interviewee at ease; explain purpose of the interview; offer a better understanding of what the referral process requires to aid tier 2 weight management to be delivered in Northumberland; explanation about how the interview will be recorded; reaffirmation of consent; and how the information will be analysed and stored; rules of confidentiality / anonymity etc.

Questions:

1. Thinking about raising the weight issue, tell me about your experience of discussing weight with patients.

   Prompts

   - *How does it feel to raise weight as an issue?*
   - *Are patients open to discussing weight problems?*
   - *Do you find a difference between genders when discussing weight?*
   - *What helps you, such as the NHS Health Check Programme, to raise the issue of weight?*
   - *What else would help to raise the issue or weight in appointments?*

2. Greater retention is often achieved when patients are ready to change, tell me how you work with / assess patient's readiness to change.

   Prompts

   - *Have you had training around the cycle of change?*
   - *Do you use any specific tools or resources to assess the patient?*
   - *What would help you to assess the patient's readiness to change?*

For peer review only

3. Thinking the information and resources available to you during the referral, do you feel you had enough information and resources to encourage patient take up of the programme?

Prompts

- *Did you have enough background information?*
- *Were the referral forms suitable / capture all the information required?*
- *Were the patient leaflets / resources suitable?*
- *Were there questions or issues raised that couldn't be answered?*
- *Was the process easy to use?*
- *What else could help you to make referrals to weight management programmes?*

4. Thinking about after you referred the patient, what happened next? (excluded after pilot)

Prompts

- *Did you get feedback from the weight management programme on the progress of your patient?*
- *Did your patients achieve weight loss?*
- *Did your patient come back and talk about their experience?*

5. What things are most likely to prevent you from making the referral a weight management programme, either commercial or Public Health funded?

Prompts

- *Are there barriers that you perceive, such as cost to the patient?*
- *Are you concerned with raising the weight issue?*
- *Is it a time factor if the patient has an appointment for anything other than a weight issue?*
- *What would help you to overcome the barriers that prevent you from making the referral?*

For peer review only

6. Is there anything else that you would like to tell me about your expectations and experiences of the weight management programme?

Focus Group Topic Guide

Set group at ease; explain purpose of the focus group; offer a better understanding of what works for people in terms of tier 2 weight management and what doesn't, aiding development of an effective programme for Northumberland residents; explanation about how the focus group will be recorded; reaffirmation of consent; and how the information will be analysed and stored; rules of confidentiality / anonymity etc.

1. Tell me a bit about what sort of weight management activity you have taken part in, in the past.

   Prompts

   - *What influence have others had on your weight management?*
   - *Do you have any particular likes/dislikes of physical activity/managing weight/nutrition*
   - *Has there been anything else that has influenced your management of weight?*

2. So thinking about the weight management programme you have undertaken, how did you find out about it?

   Prompts

   - *Who / what motivated you to attend?*
   - *What made you decide that this is the right time to look at managing your weight?*
   - *Did the time of year make a difference?*

3. Thinking about your experience of when you were referred to the weight management programme, how did you find the process?

Prompts

- *What type of health professional referred you? (GP / Practice Nurse)*
- *Did you specifically attend Primary Care to discuss your weight?*
- *How was weight raised?*
- *What did the referrer explain to you about the programme? Did you get enough information?*
- *How long was it from your referral from Primary Care to the first assessment in the weight management programme; was this what you expected? Were you still motivated?*

4. How did you feel about being referred?

- *Prompts*
- *How confident did you feel about taking part in the programme?*
- *Was there anything that you were particularly looking forward to?*
- *Was there anything that you were worried about?*

5. What did you hope to achieve by taking part in the weight management programme?

Prompts

- *What were your expectations when you start attending the scheme?*
- *Have there been changes to your health that you expected happen as a result of participation?*
- *How quickly did you expect to see these changes? And did this happen?*

6. Thinking about after you were referred, what happened next?

Prompts

For peer review only

- *How long after referral did it take to be contacted by the Active Northumberland?*
- *What information did you receive prior to the initial consultation?*
- *How comfortable did you feel coming to the initial consultation?*

7. What influenced you most to attend the weight management programme?

Prompts

- *What did you expect from the staff?*
- *How important to you were changes in health or weight?*
- *Why were the influences raised important?*

8. What things were most likely to prevent you from attending the programme?

Prompts

- *Tell me about any worries you might have had about health issues.*
- *Tell me about any other things, such as other commitments, that might have stopped you from attending*
- *Did any of these issues arise? How did you overcome these issues?*

9. Now that you have completed the programme, tell me how did you felt about undertaking the weight management programme?

Prompts

  - *Did you achieve the health / weight outcomes you expected?*
  - *Why do you think it worked or not for you?*
  - *Do you feel you now have the tools to continue to make positive lifestyle choices?*
  - *Is there something that will prevent you to continue to make positive lifestyle choices?*

For peer review only

10. Is there anything else that you would like to tell me about your expectations and experiences of the weight management programme?

For peer review only

Prototyping for public health in a local context: a streamlined evaluation of a community-based weight management programme (Momenta), Northumberland, UK

Journal:	*BMJ Open*
Manuscript ID	bmjopen-2019-029718.R2
Article Type:	Original research
Date Submitted by the Author:	12-Aug-2019
Complete List of Authors:	Dodd-Reynolds, Caroline; Durham University, Department of Sport and Exercise Sciences; Durham University, Wolfson Research Institute for Health and Wellbeing Physical Activity Special Interest Group<br>Nevens, Lisa; Northumbria Healthcare NHS Foundation Trust Community Services Business Unit<br>Oliver, Emily; Durham University, Wolfson Research Institute for Health and Wellbeing Physical Activity Special Interest Group; Durham University, Deaprtment of Sport and Exercise Sciences,<br>Finch, Tracy; Northumbria University, Nursing, Midwifery and Health<br>Lake, Amelia; Department of Science, School of Science, Engineering and Design, Teesside University<br>Hanson, Coral; Edinburgh Napier University, School of Health and Social Care; Durham University, Wolfson Research Institute for Health and Wellbeing Physical Activity Special Interest Group
**Primary Subject Heading**:	Public health
Secondary Subject Heading:	Nutrition and metabolism
Keywords:	public health evaluation, prototyping, implementation, community weight management, NUTRITION & DIETETICS, exercise referral

SCHOLARONE™
Manuscripts

Prototyping for public health in a local context: a streamlined evaluation of a community-based weight management programme (Momenta), Northumberland, UK

Caroline J Dodd-Reynolds[1,2] (corresponding author*), Lisa Nevens[3], Emily J Oliver[1,2], Tracy Finch[4], Amelia A Lake[56] Coral L Hanson[7]

[1]Department of Sport and Exercise Sciences, Durham University, Durham, DH1 3HN, UK*.

Email: caroline.dodd-reynolds@durham.ac.uk Tel: +44(0)191 3342000

[2]Wolfson Research Institute for Health and Wellbeing Physical Activity Special Interest Group, Durham University, Durham, UK.

[3]Northumbria Healthcare NHS Foundation Trust, Northumberland, UK.

[4]Department of Nursing, Midwifery and Health, Northumbria University, Newcastle upon Tyne, UK

[5]Department of Science, School of Science, Engineering and Design, Teesside University, Teesside, UK

[6]Fuse – UKCRC Centre for Translational Research in Public Health, UK

[7]School of Health and Social Care, Edinburgh Napier University, Edinburgh, UK

Word count: 4214

Keywords: Public health evaluation, protoyping, implementation, community weight management, diet, exercise referral

For peer review only

ABSTRACT

Objectives Stakeholder co-production in design of public health programmes may reduce the 'implementation gap' but can be time-consuming and costly. Prototyping, iterative refining relevant to delivery context, offers a potential solution. This evaluation explored implementation and lessons learned for a 12-week referral-based weight-management programme, 'Momenta', along with feasibility of an iterative prototyping evaluation framework.

Design Mixed methods evaluation: qualitative exploration of implementation with referrers and service users; preliminary analysis of anonymised quantitative service data (12 and 52 weeks).

Setting Two leisure centres in Northumberland, northeast England.

Participants Individual interviews with referring professionals (n=5) and focus groups with service users (n=13). Individuals (n=182) referred by healthcare professionals (quantitative data).

Interventions Three 12-week programme iterations: Momenta (n=59), Momenta-Fitness membership (Momenta-Fitness (n=58), and Fitness membership only (n=65).

Primary and secondary outcome measures Primary outcome: Qualitative themes developed through stakeholder-engagement. Secondary outcomes included preliminary exploration of recruitment, uptake, retention, and changes in weight, BMI waist circumference and psychological wellbeing.

Results Service users reported positive experiences of Momenta. Implementation gaps were revealed around the referral process and practitioner knowledge. Prototyping enabled

For peer review only

iterative refinements such as broadening inclusion criteria. Uptake and 12-week retention were higher for Momenta (84.7%, 45.8%) and Momenta-Fitness (93.1%, 60.3%) versus Fitness-only (75.4%, 24.6%). Exploration of other preliminary outcomes suggested potential for within-group weight loss and increased psychological wellbeing for Momenta and Momenta-Fitness at programme end. 52-week follow-up data were limited (32%, 33%, and 6% of those who started Momenta, Momenta-Fitness and Fitness respectively) but suggested potential for weight loss maintenance in Momenta-Fitness.

Conclusions Identification of issues within the referral process enabled real-time iterative refinement, whilst lessons learned may be of value for local implementation of 'off-the-shelf' weight management packages more generally. Our preliminary data suggest that Momenta has potential for weight loss, particularly when offered with a fitness membership.

For peer review only - http://bmjopen.bmj.com/site/about/guidelines.xhtml

ARTICLE SUMMARY

Strengths and limitations of this study

- This study advances understanding about whether prototyping is a time-efficient and cost-effective approach to design and implementation of public health programmes.
- This mixed methods evaluation provides insight into the implementation of an 'off-the-shelf' weight management programme, in a local context.
- Embedding stakeholders' views throughout the entire evaluation process allowed for ongoing, iterative refinement.
- A limitation to the quantitative component is the small sample size and rate of missing data at 1 year; findings should thus be interpreted with caution.
- Qualitative interviews and focus groups can only provide information about what participants recall about their experiences, meaning that there is a potential for recall bias.

For peer review only - http://bmjopen.bmj.com/site/about/guidelines.xhtml

INTRODUCTION

Failure to implement effective public health interventions when programmes are scaled up or transferred across contexts is widely reported.[1] Proposed approaches attempting to address this implementation gap include; effectiveness-implementation hybrid designs,[2] linking action to theory and models based on theory,[3] and application of the replicating effective programmes framework.[4] Common to all is advocacy of a developmental process reflecting on existing knowledge about the target population and planned programme prior to service delivery. Furthermore, engagement of service users is encouraged at all stages of intervention and evaluation design in MRC guidance.[5] Although this increases the likelihood of services meeting all stakeholder needs, concerns about the practical, personal, and professional costs of co-production have been raised.[6] Resulting well-designed services will be tailored to a problem that may have changed during the time spent developing the intervention. Additionally, public access may be delayed. Resource-pressured public health services must therefore consider pragmatic alternatives to service design and implementation. In this paper, we explore a novel evaluation approach to these implementation challenges, focusing on a problem high on the public health agenda: obesity and overweight.

Targeting elevated weight status is a public health priority, obesity being a recognised risk factor for many negative physical and psychological health outcomes.[7-11] In England for example, obesity and overweight are associated with 30,000 deaths and an estimated National Health Service cost of £6.1 billion per annum.[12] Globally, countries with higher income inequalities tend to have higher rates of obesity.[13] Excess weight is also associated with widening social and economic deprivation,[14] with calls to improve the effectiveness of behaviour change interventions for low-income groups.[15] There is a clear need for effective

For peer review only - http://bmjopen.bmj.com/site/about/guidelines.xhtml

public health programmes that can be refined according to local need, especially in areas with substantial deprivation. This evaluation focuses on Northumberland, in northeast England. Northumberland is one of the lowest ranked counties in England by Gross Value Added per capita (£16,140).[16] Unemployment is higher (5.5% versus 4.8%) than the England average[17] and Northumberland public health spend per person is £53, compared to a £59 national average.[18] 63.8% of adults are classified as having excess weight, higher than the national average of 61.3%.[19]

The need for innovation within public health has been postulated, shifting away from the traditional linear pre-conceived and evidence-based model.[20] For example, Parry and colleagues[21] call for research to explore not only how a programme works, but also the context and requirements for any adaptations. One such approach is prototyping[22] where projects test innovations iteratively, with ongoing refinement considering the interplay between a programme and its delivery context. Evaluation and public health teams are able to communicate at all stages of the programme, with evaluation recommendations incorporated via a rapid-cycle basis.[21] A small number of studies to date, for example in drug prevention[22] and web-based support of long-term weight loss[23] have demonstrated efficiencies when including elements of prototyping within programme development (including time, adaptation to context and cost). Such an approach seems particularly well-suited to weight management, where there are many examples of 'good' practice, or effectiveness, but no clear consensus on 'best' practice at service-delivery level. There is also limited understanding of how 'scaling up' and adapting of programmes or interventions to local contexts may impact on effectiveness. This evaluation has particular value therefore in testing a prototyping approach for a weight management programme, delivered and adapted 'in real-time', at local authority level.

For peer review only - http://bmjopen.bmj.com/site/about/guidelines.xhtml

The aim was to explore implementation of an 'off-the-shelf' weight management programme, Momenta[24], in a challenging context. Specific objectives were to explore local implementation, consider feasibility of the iterative prototyping evaluation framework and explore preliminary outcome domains including recruitment, retention, weight change and wellbeing.

METHODS

The prototyping process: local context and evaluation design

A local authority health needs assessment identified a gap in provision for a lifestyle-based weight management referral programme within Northumberland. Adults with overweight or obesity were at the time eligible for referral to the Northumberland exercise referral scheme (ERS), however previous evaluation demonstrated modest weight loss[25] and body mass index (BMI) >30 kg/m^2 was negatively associated with adherence.[26] Thus 'Momenta' was commissioned for local adaptation and delivery. The Momenta programme incorporates evidence-based behaviour change techniques and is designed to be delivered by fitness professionals in a leisure environment.[24] Developed by the MEND childhood weight management programme[27] designers, this 12-week programme aims to facilitate weight loss by engaging participants in 12 key behaviours broadly encompassing psychology, diet and physical activity (supplementary file 1). Briefly, Momenta sessions explored topics using interactive and experiential learning techniques including brainstorming, group activities and discussion, quizzes and games. At the end of each session, participants set goals focusing on one of the 12 key behaviours. At the beginning of each session, the group discussed the

For peer review only

For peer review only - http://bmjopen.bmj.com/site/about/guidelines.xhtml

previous weeks' goals by exchanging stories and brainstorming challenges. All interventions were free to service users.

The local Leisure Trust was commissioned to deliver a pilot Momenta programme. Commissioners and providers had ideas about alternative delivery options and due to an established academic relationship, asked the study team for advice about robust evaluation that would allow for feedback in real time and at the end of the pilot. Stakeholder meetings were held with Public Health staff (n=2), Leisure Trust managers (n=3), delivery staff (n=2) and Momenta programme developers (n=2). As part of the prototyping process, members of the evaluation team (CDR, EO) provided guidance on evaluation design and light touch advice about tools to explore preliminary effectiveness. The evaluation was thus co-produced to ensure a robust framework, whilst meeting strategic local needs. For example, commissioners were concerned about meeting recruitment targets for an existing specialist weight management service used mainly for pre-bariatric patients and Momenta was initially commissioned for patients with BMI 25.0-29.9 kg/m^2, although this was later amended. Furthermore, commissioners were keen to consider accessibility of provision and wished to explore offering free gym, swimming and fitness class membership. The evaluation was designed to accommodate this.

The programme was ultimately delivered at two leisure sites situated within the 20% and 50% most deprived neighbourhoods in the country. Six General Practice (GP) surgeries, identified as the best referrers to the existing ERS, were asked to refer suitable patients to Momenta. The programme manager and the public health improvement manager (LN) attended practice meetings to articulate referral criteria and disseminate advertising materials.

For peer review only - http://bmjopen.bmj.com/site/about/guidelines.xhtml

Attendance varied from two to all practice staff, meaning that in some surgeries knowledge of the programme was reliant on dissemination by those who attended.

A mixed methods evaluation was agreed between the evaluation team and commissioners. Qualitative and quantitative components were conducted concurrently and had equal status.[28] Prototyping allowed for iterative changes to be made to the implementation and delivery of the programme in real time and we reflect upon these in the results and discussion.

Referrals by healthcare professionals (HCPs) were via a standardised form to the appropriate leisure site. Prior to programme commencement the Leisure Trust, in conjunction with the Momenta programme designer and members of the evaluation team, held a training day for delivery staff. Although staff were qualified to deliver Momenta, extra bespoke training (including role-play scenarios and problem-solving discussions) was delivered by the clinical psychologist who designed Momenta. The evaluation team (CDR, CH) trained delivery staff in international standard anthropometric techniques[29] and familiarised them with other evaluation measures.

Programme providers allocated service users into one of three comparison groups:
a) Combined Momenta plus fitness membership (Momenta-Fitness);
b) Momenta;
c) Fitness membership (Fitness only).
Participants were allocated into groups in order of receipt (the first referral form received was allocated to Momenta-Fitness, the second form to Momenta, the third form to fitness only etc.). The provider then contacted participants by telephone to arrange attendance. If a participant was unable to attend the allocated group, (e.g. due to inconvenient session times)

For peer review only - http://bmjopen.bmj.com/site/about/guidelines.xhtml

provider allocated them to a different group after discussion. Due to maximum recommended Momenta group size, referrals were split into delivery cohorts of 15, with groups rolling through March 2015 to April 2016.

Implementation effectiveness for the referral process was explored through semi-structured interviews with referring HCPs (undertaken at referring surgeries) and focus groups with service users (in leisure centres). All were conducted by LN during March-July 2015, as part of her Public Health Master's degree (which contained qualitative methods training), mentored by TF, an experienced qualitative researcher. LN was employed as a member of the Northumberland public health team at the time of the evaluation. Questions are included in supplementary file 2. Data were audio-recorded. Results are reported using the Consolidated criteria for Reporting Qualitative research guidelines.[30]

Practice managers from all six referring surgeries were sent an email invitation for staff to take part (n = 84), (General Practitioner = 53, Practice Nurse = 18, Health Care Assistant = 13). Individual correspondence was sent to those agreeing. LN informed participants about her employment status and that the study aimed to understand implementation issues. Interviews aimed to explore HCPs' referral experiences; raising weight issues; assessing readiness to change; marketing and referral materials; and the referral process. Interview questions were pilot tested with public health colleagues to assess timing and ensure validity. One question (*Thinking about after you referred the patient, what happened next?*) was omitted after piloting as it was realised HCPs would not have had patient feedback at that point. Interviews lasted on average 26 minutes and were transcribed verbatim. Data were analysed following each interview, with developing themes considered to determine whether

For peer review only - http://bmjopen.bmj.com/site/about/guidelines.xhtml

questions required refinement. Initial themes generated from the first two interviews did not change and thus questions remained constant, although prompts were added.

During the initial assessment session for the first wave of referrals, all (n = 39) were given a written invitation to participate in a series of focus groups at programme-end to explore experiences. Emphasis was placed on the referral process, initial expectations and experiences of participation; how weight issues were raised by HCPs; time from referral to initial assessment; and facilitators and barriers to taking part. Focus groups lasted between 26 and 44 minutes.

Preliminary outcome data were collected to provide an initial indication of programme success. These included anthropometric measurements to determine weight change. Well-being measures were of specific interest to commissioners. Sociodemographic information was also available as indicated.

Age, gender and postcode (for index of multiple deprivation, IMD) were recorded by referring HCPs on the referral form. Employment status, level of education, cohort wave and programme group were recorded by leisure staff, who also measured weight and stature (without shoes or bulky clothing) and waist circumference at baseline and programme end. Measures were taken in at least duplicate, using standardised tools in accordance with international standards[29] using SECA 761 scales, a Leicester portable stadiometer and anthropometry tape. Body mass index was calculated and classified according to WHO guidelines.[31] The Warwick-Edinburgh Mental Well-being Scale,[32] and the Hospital Anxiety and Depression Scale (HADS)[33] were administered at each time-point. Attendance at Momenta and leisure centre usage was monitored via swipe-card tracking. 52 weeks after

For peer review only - http://bmjopen.bmj.com/site/about/guidelines.xhtml

commencing the programme, service users were invited to attend a follow-up session, where leisure staff repeated physiological and psychological measures. Programme providers collected and collated quantitative data and provided an anonymised dataset to the evaluation team for analysis.

Patient and public involvement

Patients and the public were not involved in the choice of evaluation topic, assisting in the study design, advising on the project or in carrying out the evaluation.

Data analyses

Qualitative data were audio-recorded and transcribed by LN using a thematic process.[34] Data were organised according to concepts, key themes and developing categories. Data coding was discussed with TF, allowing comparison of data interpretation and subsequent coding refinement. Evolving key themes were refined through the analysis process and subsequent cross-sectional thematic labelling of data, thus generating deeper understanding. Where possible, key phrases or expressions identified from interviews and focus groups were retained within coding and thematic labelling. A public health colleague helped to verify interpretations of the data and appropriateness of codes applied. Once initial interviews were coded this framework was applied to remaining data. Notes taken during focus groups helped to contextualise when developing themes and included information about dynamics within groups, such as influence, disagreement, humour and peer exposure.

The anonymised quantitative dataset was analysed using PSAW Statistics V.22. Descriptive statistics were calculated for age, gender, IMD, employment status, initial BMI, leisure site,

For peer review only - http://bmjopen.bmj.com/site/about/guidelines.xhtml

level of education, and uptake and adherence. Distribution and normality of measures (weight, BMI, waist circumference, psychological wellbeing and attendance) were assessed using Shapiro Wilk tests and median and interquartile range (IQR) scores calculated for each group at baseline and 12 weeks (attendance, 12 weeks only). For information and general descriptive purposes, preliminary inferential analyses were undertaken. Using complete cases, Kruskal-Wallis H tests were used to explore between-group differences at baseline and at 12 weeks and Wilcoxon-signed rank tests explored repeated measures differences between baseline and 12-week scores. Complete cases available at 52 weeks (n = 37) were considered similarly, but via separate analyses due to limited available data across comparison groups.

RESULTS

Between December 2014 and March 2016, the programme received 182 referrals and was delivered in four cohorts across leisure sites. Due to initial low levels of recruitment, the first cohort did not start until March 2015. Referrals were mainly female (83%) and 30.6% lived in the 20% most deprived areas (table 1).

For peer review only - http://bmjopen.bmj.com/site/about/guidelines.xhtml

Table 1. Demographic characteristics of referrals who started the programme (n=153)

	Median	IQR
**Age (years)**	53	24
**Gender**	**n**	**%**
Male	25	16.3%
Female	120	78.4%
Not stated	8	5.2%
**Initial BMI category (kg/m^2)**		
25.0-29.9	40	26.1%
30.0-34.9	73	-47.7%
35.0-39.9	27	17.6%
≥40.0	10	6.5%
Not stated	3	2.0%
**Leisure site**		
Leisure site 1 (IMD quintile 2)	69	45.1%
Leisure site 2 (IMD quintile 3)	83	54.2%
Not stated	1	0.7%
**Index of multiple deprivation**		
20% most deprived	42	27.5%
21-40%	33	21.6%
41-60%	17	11.1%
61-80%	20	13.1%
20% least deprived	35	22.9%
Not stated	6	3.9%
**Employment status**		
Employed full time	36	23.5%
Employed part time	24	15.7%
Retired	51	33.3%
Claiming incapacity benefit	5	3.3%
Claiming job seekers allowance	6	3.9%
Not stated	14	9.2%
**Level of education**		
Primary	15	9.8%
Secondary (O level/GCSE)	35	22.9%
Secondary (A level)	26	17.0%
Further education (HND)	24	15.7%
Bachelors or equivalent	21	13.7%
Masters or equivalent	5	3.3%
Not stated	27	17.6%

Age, gender and postcode (IMD calculated by the programme provider) recorded from the referral form.

Employment and level of education self-reported by participants during the first session. The provider did not follow up missing data.

BMI and leisure site recorded by the provider. Missing data not available for analysis and presumed to be data entry errors.

For peer review only

Implementation effectiveness: reflections from referring healthcare professionals

Five face-to-face semi-structured interviews took place with HCPs across five referring surgeries: two GPs, two Practice Nurses and one Health Care Assistant. HCPs perceived that four key themes influenced the effectiveness of programme implementation: (i) difficulties raising weight with patients, (ii) how gender affected patient engagement, (iii) availability of information and resources, and (iv) additional barriers constraining programme promotion.

Raising the issue of weight with patients:

Concerns about raising weight may have contributed to slow recruitment, with nurses and healthcare assistants expressing unease, *'not really up to me… well I talk about it if they want to…. Better if they [patients] bring it up.'* (Interview 2, Healthcare Assistant). GPs seemed more comfortable raising weight with patients, but with the caveat that this is easier in the context of a longer-term GP/patient relationship.

> *'the people I see I've known for a very long time… it's the rapport you have…if I'd never met anyone before and they came in for a sore throat I'm not going to say you're fat…If there was someone I'd known for a long time and it seemed relevant…I'd mention it.'* (Interview 5, GP).

Gender and engagement in the referral process:

For peer review only - http://bmjopen.bmj.com/site/about/guidelines.xhtml

Gender was highlighted as influencing the referral process, women being more likely than men to seek support for weight. This may help explain the low rate of referral for males (17%):

> *'More women talk about it…men don't really talk about weight…I do mention weight to men if I'm doing a well man [sic] but it doesn't come up really…it's a woman thing…'* (Interview 3, Practice Nurse).

Availability of information and resources:

Several interviewees highlighted training needs around programme information and resources, (e.g., additional programme information would help to engage patients). For example, the GPs both discussed the longstanding ERS and stated they needed to become more familiar with Momenta, as they had with the ERS:

> *'when we get opportunities to do things in the practice we normally discuss it, let everyone know where appropriate forms and information is and it's in your head…that didn't happen with this and I don't know why that was.'* (Interview 5, GP).

All HCPs interviewed felt the referral leaflet (provided by programme providers) was important in the process, either as a tool to promote the intervention or to convey information to patients:

> *'The leaflet was good, bright…explained the programme and patients like taking a leaflet away.'* (Interview 3, Practice Nurse)

For peer review only - http://bmjopen.bmj.com/site/about/guidelines.xhtml

Additional Barriers to Engagement:

Several sub-themes highlighted additional barriers to the referral process. The most prominent sub-themes were around initial BMI referral criteria (25.0-29.9 kg/m^2) and delayed programme start. Both implementation factors were beyond the control of the referrers, but consequently amended through iterative refinement during the prototyping process following early data analysis. Both were reported by practice nurses as exacerbating each other:

> '*we were referring but then it didn't start so people were not sure what was happening [pause]…Think it was more people were needed to start…but you know if the BMI was higher then there would have been more*.' (Interview 3, Practice Nurse).

In one case, a decision was taken to relax the referral criteria, *'…31.5 [kg/m^2]…was just outside so I just referred him*.' (Interview 4, GP).

Programme location was perceived by HCPs to overcome an existing barrier to the tier three weight management programme, as Momenta was '*round the corner for people,*' as opposed to '*a bit far away at the hospital.'* Cost barriers were also discussed, both with reference to the patient, *'in this sort of area…cost…, if you've got to pay it's a barrier.'* (Interview 4, GP), and to expected targets from Clinical Commissioning Groups (CCG),

> *'we are constantly told by the CCG that we must keep down on numbers and that if there are costs attached to this referral that would definitely impact… and that would be for all practices.'* (Interview 5, GP)

For peer review only - http://bmjopen.bmj.com/site/about/guidelines.xhtml

Implementation effectiveness: reflections from participants

Three focus groups in the leisure centres allowed programme participant voices to be heard: three females and one male from Momenta (focus group 1), three males and three females from Momenta-Fitness (focus group 2) and three females (one of whom emailed her views separately) from Fitness-only. Across the groups, 12 participants reported having lost weight and one reported weight gain. Three themes developed: (i) outcomes of the programme, (ii) facilitators and barriers to engagement, and (iii) raising the issues of weight with HCPs.

Outcomes of the programme:

Focus group findings aligned closely with quantitative outcomes in terms of the physical and psychological benefits of participation: *'[I've] lost a good bit of weight. It's been very positive for me… I'm feeling a lot more active…'* (Momenta-Fitness, Participant 5). Participants reported a sense of weight loss achievement, increased physical activity levels, and positive mood states. In addition, elements of the Momenta programme were perceived as facilitating engagement, including the *'group feeling… I looked forward to it,'* (Momenta-Fitness, Participant 4), the *'information that we got every week… so very well planned.'* (Momenta-Fitness, Participant 3) and the ongoing support e.g., *'she 'phoned me the other day to see if I was coming,'* (Momenta-Fitness, Participant 4). Momenta participants reflected back on, and identified and discussed lifestyle factors that related to their initial weight gain (e.g., *'I did the usual thing… I started eating toffees,'* Momenta-Fitness, Participant 5*),* demonstrating both self-awareness and an openness to discussing the topic.

Facilitators and barriers to engagement:

For peer review only - http://bmjopen.bmj.com/site/about/guidelines.xhtml

One participant reported being initially excluded but later allowed to take part, and others raised concerns that the initial BMI threshold for referral (25-29.9 kg/m^2) was too low, '*was a little bit high, BMI…managed to get it down... [and then] the doctor put us forward,'* (Momenta, Participant 2). Data also indicated the importance of subsidised access, particularly important in the context of a deprived region such as this, e.g., '*I also joined Weight Watchers for short period of time but found the classes too expensive,*' (Fitness-only, Participant 3, emailed response).

Raising the issue of weight with HCPs:

Some data did suggest implementation was problematic, however, this focused exclusively on the referral process. Participants overwhelmingly felt that they had opened the conversation about weight, as opposed to discussions being initiated by HCPs (e.g., '*my glucose levels were quite high but nobody ever said that I was overweight*,' Momenta-Fitness, Participant 4*).* In addition, participants perceived limitations in HCPs' knowledge of intervention components ('*she [nurse] didn't know anything about it*,' Fitness-only, Participant 1), something with potential to impact on likelihood of referral, and participants' expectations of programme success.

Preliminary outcome domains

Of all referrals, 153 (84%) attended the baseline measurement session and 78 (51% of those who started) attended the 12-week measurement session. Uptake and adherence varied by programme group (table 2).

For peer review only - http://bmjopen.bmj.com/site/about/guidelines.xhtml

Table 2. Programme uptake, adherence and attendance.

Uptake (week 1), adherence and retention (both week 12)	Momenta-Fitness		Momenta only		Fitness only	
Number referred		58		59		65
Uptake* (n, %)		54 (93.1%)		50 (84.7%)		49 (75.4%)
Uptake retention** (n, %)		35 (64.8%)		27 (54.0%)		16 (32.7%)
Uptake adherence^ (n, %)		34 (63.0%)		26 (52.0%)		8 (50.0%)
Overall retention*** (n, %)		35 (60.3%)		27 (45.8%)		16 (24.6%)
Overall adherence^^ (n, %)		34 (58.6%)		26 (44.1%)		8 (12.3%)
**Momenta session attendance**	**Momenta-Fitness**		**Momenta only**		**Fitness only**	
	**n**	**Median (IQR)**	**n**	**Median (IQR)**		
Uptake	54	9.0 (7.3)	50	9.0 (8.0)		
Dropouts	19	3.0 (3.0)	23	3.0 (5.0)		N/A
Completers^^^	35	10.0 (2.0)	27	11.0 (1.3)		
**Exercise session attendance**	**Momenta-Fitness**		**Momenta only**		**Fitness only**	
	**n**	**Median (IQR)**	**n**	**Median (IQR)**	**n**	**Median (IQR)**
Uptake	54	7.0 (16.3)	50	0.0 (4.5)	49	0.0 (1.5)
Dropouts	19	0.0 (1.0)	23	0.0 (0.0)	33	0.0 (0.0)
Completers^^^	35	10.0 (14.0)	26	0.0 (5.0)	16	4.5 (18.0)

Uptake* *participant attended baseline assessment;* ***Uptake retention**** *% of participants who attended the 12-week assessment out of those who attended the baseline assessment;* ***Uptake adherence^*** *% of participants who attended the baseline assessment who also attended ≥ 8 Momenta sessions (Momenta-Fitness and Momenta only) or gym sessions (fitness only);* ***Overall retention**** *% of all those referred who attended both baseline and 12-week assessment;* ***Overall adherence^^*** *% of all those referred who attended ≥ eight Momenta sessions (Momenta-Fitness and Momenta only) or exercise sessions (fitness only); Completers^^^ those who completed the 12-week assessment*

Physiological and psychological data were not normally distributed. Quantitative data are presented as exploratory, due to the small sample size and are presented here for information and general description. No significant differences were found between programme groups either at baseline or at 12 weeks, for any measures. Despite the small sample size, significant within-group differences between baseline and 12 weeks were evident for weight, BMI and waist circumference for Momenta-Fitness, and Momenta (Table 3). Follow-up analysis at 52-weeks (available sub-sample) showed changes were maintained for Momenta-Fitness (n =18) only.

For peer review only - http://bmjopen.bmj.com/site/about/guidelines.xhtml

Table 3. Weight, BMI and waist circumference change.

End of programme results	Median (IQR) Baseline	Median (IQR) 12 weeks	z	p	Median (IQR) Change
**Weight (kg)**					
Momenta-Fitness (n=35)	88.9 (80.5 - 100.0)	88.0 (77.2 - 95.8)	-4.531	<0.001	-2.9 (-5.1 - -1.6)
Momenta only (n=26)	87.8 (74.5 - 77.0)	83.3 (74.5 - 92.5)	-4.344	<0.001	-2.9 (-5.0 - -2.0)
Fitness only (n=15)	76.2 (71.6 - 86.9)	76.6 (70.4 - 84.6)	-0.879	0.379	0.0 (-3.2 - 1.0)
**BMI (kg/m²)**					
Momenta-Fitness (n=35)	32.0 (30.3 - 35.7)	31.3 (29.2 – 35.3)	-4-494	<0.001	-1.1 (-1.9 - -0.6)
Momenta only (n=26)	32.0 (30.0 - 34.5)	31.3 (28.6 – 33.6)	-4.356	<0.001	-1.2 (-1.6 - -0.8)
Fitness only (n=14)	29.2 (27.3 - 33.0)	29.7 (27.0 – 33.3)	-0.454	0.650	0.1 (-1.2 - +0.4)
**Waist circumference (cm)**					
Momenta-fitness (n=35)	106.0 (98.0 - 115.0)	99.0 (93.0 - 110.0)	-4.996	<0.001	-7.0 (-9.5 - -5.0)
Momenta only (n=25)	108.0 (99.5 - 114.5)	101.0 (93.8 - 111.5)	-4.166	<0.001	-5.0 (-7.3 - -2.5)
Fitness only (n=11)	90.0 (87.0 – 95.0)	91.0 (90.0 - 96.0)	0.358	0.650	1.0 (-3.0 - 3.0)

52-week follow-up	Median (IQR) Baseline	Median (IQR) 52 weeks	z	p	Median (IQR) Change
**Weight (kg)**					
Momenta-Fitness (n=18)	95.2 (87.1 - 101.4)	91.4 (82.7 - 95.9)	-3.006	<0.001	-4.8 (-6.2 - -1.5)
Momenta only (n=16)	84.7 (72.3 - 95.2)	82.7 (73.2 - 94.6)	-1.533	0.120	-0.7 (-7.6 - 0.8)
*Fitness only (n=3)	73.4 (69.5 - 80.2)	70.3 (66.0 - 87.0)			0.9 (-7.4 – 6.9)
**BMI (kg/m²)**					
Momenta-Fitness (n=18)	32.0 (30.49 - 35.1)	30.8 (28.7 - 34.0)	-3.157	<0.05	-1.7 (-2.0 - -0.6)
Momenta only (n=16)	31.7 (29.3 – 33.9)	31.1 (26.7 – 33.6)	-1.603	0.109	-0.3 (-2.3 - 0.3)
*Fitness only (n=3)	27.6 (27.5 - 30.5)	27.8 (24.8 – 33.2)			0.3 (24.8 - 33.2)
**Waist circumference (cm)**					
Momenta-Fitness (n=18)	109.0 (101.0 - 114.8)	100.5 (94.8 - 107.3)	-3.221	<0.001	-6.0 (-13.3 - -1.75)
Momenta only (n=16)	106.0 (94.5 - 115.8)	103.5 (98.5 - 113.3)	-0.780	0.938	-2.5 (-9.0 - -10.0)
*Fitness only (n=3)	89.0 (87.0 – 95.0)	90.0 (90.0 – 101.0)			3.0 (90.0 – 101.0)

** Fitness only n=3 therefore median and range reported and no statistical test completed.*

Differences in mental wellbeing, depression and anxiety were not apparent between groups, however improvements in mental wellbeing, and reductions in depression and anxiety were evident between baseline and 12 weeks for Momenta-Fitness, and Momenta groups only (Table 4), although the magnitude of change was similar for all groups. The changes observed, though small, could be argued to be functionally and clinically meaningful, with a minimal important difference of 1.5 points previously identified for the HADS, for example[35]. 52-week sub-sample analysis showed that significant improvements for wellbeing

For peer review only

For peer review only - http://bmjopen.bmj.com/site/about/guidelines.xhtml

and depression were maintained for Momenta-Fitness (n=18), and wellbeing and anxiety for Momenta (n=16).

Table 4. Wellbeing, anxiety and depression measures change.

End of programme results	Median (IQR) Baseline	Median (IQR) 12 weeks	z	p	Median (IQR) Change
**Mental wellbeing scale**					
Momenta-Fitness (n=29)	46.0 (40.0 - 51.5)	53.0 (40.0 - 51.5)	3.810	<0.001	5.0 (1.5 - 12.0)
Momenta only (n=23)	49.0 (39.0 - 58.0)	55.0 (51.0 - 63.0)	2.818	<0.05	6.0 (-1.0 - 10.0)
Fitness only (n=13)	47.0 (40.5 - 59.5)	46.0 (42.0 - 63.5)	0.157	0.875	0.0 (-4.0 - 5.0)
**Anxiety scale**					
Momenta-Fitness (n=28)	5.5 (4.0 - 9.8)	4.5 (2.0 - 7.0)	-3.027	<0.001	-1.0 (-3.0 - 1.0)
Momenta only (n=23)	8.0 (6.0 - 10.0)	4.0 (2.5 - 9.0)	-2.329	<0.05	-1.0 (-3.0 - 0.0)
Fitness only (n=13)	8.0 (3.5 - 10.0)	6.0 (4.0 - 9.0)	-0.499	0.618	-1.0 (-2.0 - 2.0)
**Depression scale**					
Momenta-Fitness (n=28)	5.5 (3.3 - 8.0)	2.0 (1.0 - 6.0)	-3.214	<0.05	-2.5 (-4.8 - -0.3)
Momenta only (n=23)	5.0 (3.0 - 7.5)	3.0 (1.0 - 5.0)	-3.049	<0.05	-1.0 (-4.5 - 1.0)
Fitness only (n=13)	4.0 (2.0 - 8.5)	2.0 (2.0 - 7.0)	-1.226	0.220	-2.0 (-4.5 - 0.0)
**52-week follow-up**	**Median (IQR) Baseline**	**Median (IQR) 52 weeks**	**z**	**p**	**Median (IQR) Change**
**Mental wellbeing scale**					
Momenta-Fitness (n=15)	44.0 (39.0 - 52.0)	55.0 (48.0 - 59.0)	2.984	<0.05	5.0 (3.0 - 15.0)
Momenta only (n=13)	58.0 (47.5 - 59.0)	56.0 (54.0 - 63.5)	2.282	<0.05	4.0 (0.5 - 6.5)
*Fitness only (n=3)	47.0 (34.0 – 64.0)	58.0 (45.0 – 60.0)			-2.0 (-6.0 – 26.0)
**Anxiety scale**					
Momenta-Fitness (n=15)	6.0 (2.0 - 10.0)	2.0 (1.0 - 7.0)	-1.785	0.074	-3.0 (-6.0 - 0.0)
Momenta only (n=15)	7.0 (4.0 - 9.0)	5.0 (1.0 - 8.0)	-1.990	<0.05	-3.0 (-4.0 - 0.0)
*Fitness only (n=3)	9.0 (5.0 – 10.0)	2.0 (1.0 – 8.0)			-3.0 (-8.00 - -2.0)
**Depression scale**					
Momenta-Fitness (n=15)	7.0 (3.3 - 11.3)	3.5 (1.0 - 6.0)	-2.908	<0.05	-3.5 (-6.3 - -0.8)
Momenta only (n=15)	4.0 (1.0 - 6.0)	3.0 (1.0 - 4.0)	-0.762	0.446	0.0 (-2.0 - 1.0)
*Fitness only (n=3)	3.0 (0.0 – 8.0)	2.0 (1.0 – 8.0)			1.0 (-8.0 – 5.0)

** Fitness only n=3 therefore median and range reported and no statistical test completed.*

Overall, the results suggested those who participated in the two groups incorporating Momenta, had enhanced physical and psychological health indicators from baseline, whereas those who had only free fitness membership did not. From the small follow-up sample, there

For peer review only

For peer review only - http://bmjopen.bmj.com/site/about/guidelines.xhtml

is scope to suggest that the combination of Momenta and fitness membership may produce favourable outcomes at 52 weeks.

Iterative refinements throughout the evaluation process

Here we list a number of implementation adjustments which were made throughout the evaluation process, facilitated via the prototyping framework. Real-time advice from Commissioners was considered during early stages of implementation, regarding the nature of comparison offers (e.g. fitness access) and thus initial design and outcome measurements were adapted prior to referrals being made. To better-target recruitment and change the process of engagement at referral point, entry criteria were altered (BMI ≥30 kg/m^2) mid-way through programme delivery. On-site implementation of the service offer was adapted in response to delivery staff feedback: increased resource was made available, for example additional staffing to support delivery for the first wave of referrals. Furthermore, staff were given additional time for Momenta session preparation and session delivery times were extended. Follow-up activities (i.e., text or telephone contact) were implemented by staff during the process, to encourage adherence.

DISCUSSION

We explored 'prototyping', as a cost-effective and time-efficient approach to public health evaluation, via an 'off-the-shelf' weight management programme implemented in a local context of mixed and high deprivation.

For peer review only - http://bmjopen.bmj.com/site/about/guidelines.xhtml

Experiences of both referrers and referrals highlighted that HCPs needed to be better-informed and more confident raising weight-related conversations. Whilst patient-led action is desirable, staff reluctance to raise weight issues may mean that opportunities for engagement of less knowledgeable or motivated patients will be missed. The problematic positioning of GPs within obesity care has been highlighted previously,[36] with a range of strategies to change HCPs' behaviour resulting in little or no change to patients' weight. A practical training need is highlighted for those working at the patient-practitioner interface, however communication with patients about weight may well be hindered by the 'stigma' attached to obesity.[37] This has wider implications for patient outcomes and requires further exploration through the implementation process. Additionally, HPCs need better understanding of referral-based public health programmes offered. Despite efforts of programme and public health managers, awareness was reportedly low for some referring professionals. We suggest consideration of resource-efficient ways to signpost both HPCs and patients themselves as part of the implementation process.

This programme was delivered across a social gradient in a region with low health indices and areas of high deprivation. Some issues in relation to inequalities and service access for future community-based weight management programmes were highlighted. Only 17% of referrals to Momenta were males. Gender bias in weight management referral has been reported elsewhere,[38-39] and interviews showed that practitioners struggled to raise the topic of weight with male patients. Alternative referral strategies have been employed in other settings in an attempt to overcome this.[40] Marketing in other community spaces, or targeted postal referrals could be explored in future implementation. The initial decision to restrict referral to overweight-only substantially impacted on referral rates, with HCPs and referrals indicating they felt limited until this restriction was reversed. Had this continued, worsening

For peer review only - http://bmjopen.bmj.com/site/about/guidelines.xhtml

health inequalities may have been an unintended consequence, something to be actively avoided within public health programmes [41]. The roles of, and interactions between, those operating in the 'system' (i.e. the context within which the intervention operates) must be considered at the point of implementation to minimise any impact from unintended consequences.[5] In practical terms, this may be through continued dialogue with commissioners, referring professionals and referrals themselves, something which prototyping evaluation allows.

Quantitative data should be interpreted as exploratory, due to the relatively small number of complete cases, however lessons can be learned from these data both in terms of preliminary outcomes and engagement/dropout. Participation in Momenta and Momenta-Fitness resulted in 12-week weight loss for those who completed the programme. Free fitness membership without the weight-management programme was poorly engaged with and did not lead to weight change. A small sub-sample who attended follow-up demonstrated that after one year, weight reductions equivalent to ~4% could be maintained for Momenta-Fitness. We caution that while this might be best interpreted as hypothesis-generating for future evaluations, given these effects emerged despite an underpowered sample it is worth briefly considering potential mechanisms here. Providing free access to fitness facilities alongside the behaviour change programme may allow for continuous and self-driven behaviour change[42] and sustaining optimal changes in adiposity over 12 months in those who remained engaged.[43] Swipe card monitoring during the initial 12-week period indicated that fitness sessions were accessed an average 10 occasions for this group, whereas no access was apparent for Momenta, despite Momenta sessions being held in leisure centres. This could be important for community providers making decisions about delivery location. Both Momenta groups reported improved wellbeing, and reduced anxiety and depression at 12-weeks suggesting

For peer review only - http://bmjopen.bmj.com/site/about/guidelines.xhtml

that the behavioural intervention may drive this effect. This is consistent with previous work reporting co-varying changes in weight loss, depression, and quality of life in weight management services.[44] It is unclear whether the primary mechanism was weight loss, or the wider social benefits of participation. Both were valued in the qualitative data. Our preliminary evidence of maintained improvements in wellbeing for these groups at 52 weeks is particularly relevant given previously evidenced associations between poor mental health, and obesity and overweight status.[45] Long-term follow-up rates will need to be considered in future similar programmes and we suggest year-long follow-up (at least) is included as a key programme component from the outset. Consideration should be given to how providers can maintain contact with participants after programme end to increase likelihood of successful follow-up. Potential 'light touch' support after 12 weeks may be helpful and other means of obtaining follow-up data should be explored where service users disengage. Reasons for disengagement might also be usefully explored in future work.

Given that no systematic problems emerged with service-user's experiences of the programme itself, our findings lend support to a streamlined approach to involvement of all stakeholders in programme implementation. We suggest that prototyping demonstrates opportunities for off-the-shelf programmes to be pragmatically moulded to local context, in real-time. Many of the iterative changes made were staff-driven. This demonstrates that real-time consideration of feedback from on-site delivery teams can be important to the implementation process. Some of the adjustments required commissioning action, as they had resource implications; others needed advice from the evaluation team. Interestingly changes made throughout the process generally focused on both staff and participant experience.

view only

For peer review only - http://bmjopen.bmj.com/site/about/guidelines.xhtml

Emergence of some negative experiences of referral suggests, however, that prototyping can be problematic without networks or channels for ensuring key outcomes are widely communicated to relevant stakeholders. Overall, the evaluation demonstrated that a balance is needed to allow quick and efficient adaptation of off-the-shelf programmes, but with focused professional user engagement in the early stages of development. The prototyping approach had particular utility given that project resources were limited and meant that issues were identified and acted upon rapidly. While the programme may have progressed similarly without this, prototyping provided a greater structure for, and confidence in, on-going refinements. This was achieved via the support provided by academics, public health practitioners and providers. Fundamentally, adopting a prototyping approach enabled the delivery of a new service to an in-need population, alongside the generation of initial evidence of local effectiveness. A minimum of 1 kg weight-loss at 3 months, and 0.7 kg at 12-months have been suggested as thresholds to influence decisions over commissioning of weight-loss services.[46] Our preliminary data suggests that Momenta may have potential to meet or even exceed these thresholds, showing particular promise when implemented in conjunction with free fitness provision.

Demonstrating preliminary effectiveness is of limited use, however, unless a successful programme in one area may be adapted and implemented to suit a different context, for example through sharing local-level knowledge, interactions and behaviours of individuals within different parts of that system.[47] The process for scaling-up of effective health interventions to broader policy and practice takes years[48] and certainly within the obesity literature, has been dominated by initiatives that consider effectiveness but not implementation across specific settings.[49-50] We recommend prototyping might be built into

For peer review only - http://bmjopen.bmj.com/site/about/guidelines.xhtml

larger public health evaluations providing that the original programme has a sound theoretical basis, and iterative refinement is engaged with by all stakeholders from the outset.

CONCLUSION

The Momenta programme was experienced positively by those who attended. Issues with the referral process need to be explored further, however other refinements were feasible during delivery. Promising preliminary outcome data for 'Momenta', particularly in conjunction with a free fitness offer, implies potential for the scheme within future commissioning. This evaluation extends the literature by exploring prototyping for a complex problem, community weight-management, in a challenging setting, demonstrating streamlined implementation of an 'off-the-shelf' weight management programme. This resource-effective approach is highly relevant in the context of health inequalities and public health sector funding constraints.

For peer review only

For peer review only - http://bmjopen.bmj.com/site/about/guidelines.xhtml

Acknowledgements: Many thanks to Jordan Bell, Nicole Rowley and Ross Davison, who collected and collated raw quantitative data.

Author Contributions: CDR, CH and EO contributed to design of the quantitative evaluation, data analysis and preparation the final document. LN contributed to design and analyses of the qualitative evaluation component. TF contributed to qualitative design and analysis and preparation of the final document. AL contributed to design of the quantitative evaluation and preparation of the final document.

Funding: The quantitative evaluation component was supported by funding from the Wolfson Research Institute for Health and Wellbeing, Durham University. Amelia Lake is a member of Fuse, the Centre for Translational Research in Publicasters Health (www.fuse.ac.uk). Fuse is a UK Clinical Research Collaboration (UKCRC) Public Health Research Centre of Excellence. Funding for Fuse from the British Heart Foundation, Cancer Research UK, Economic and Social Research Council, Medical Research Council, the National Institute for Health Research, under the auspices of the UKCRC, is gratefully acknowledged. The views expressed in this paper do not necessarily represent those of the funders or UKCRC. The funders had no role in study design, data collection and analysis, decision to publish, or preparation of the manuscript.

Competing interests: CH is a former employee of the Leisure Trust and was instrumental in initiating the evaluation. She was subsequently employed as a research assistant at Durham University, however was not involved in any data collection or entry, only accessing an anonymised database submitted to the University. LN was a Public Health Improvement

For peer review only

Manager within the Public Health Team and had responsibility for commissioning the Momenta programme. The qualitative evaluation component was submitted in partial fulfilment of her Master's in Public Health at Newcastle University.

Participant consent: Consent was obtained for face-to-face interviews and focus groups. Service users were informed in writing of the nature of the quantitative service evaluation and how to withdraw from it. The presented data are anonymised with risk of identification low.

Ethics approval: Ethical advice was sought from the local Research Manager of North of England Commissioning Support, and this project was classed as a service evaluation in line with National Research Ethics Service guidance.

Data availability statement: No additional data are available because this was a service evaluation.

For peer review only

REFERENCES

1. Haines A, Kuruvilla S, Borchert M. Bridging the implementation gap between knowledge and action for health. (Theme Papers). *Bull World Health Organ* 2004;82:724.

2. Curran GM, Bauer M, Mittman B, *et al*. Effectiveness-implementation Hybrid Designs: Combining Elements of Clinical Effectiveness and Implementation Research to Enhance Public Health Impact. *Med Care* 2012;50:217-26.

3. Sales A, Smith J, Curran G, *et al*. Models, Strategies, and Tools: Theory in Implementing Evidence-Based Findings into Health Care Practice. *J Gen Int Med* 2006;21:S43-S9.

4. Kilbourne AM, Neumann MS, Pincus HA, *et al*. Implementing evidence-based interventions in health care: application of the replicating effective programs framework. *Implement Sci* 2007;2:42.

5. Medical Research Council. Developing and evaluating complex interventions: new guidance. UK: Medical Research Council, 2006. https://mrc.ukri.org/documents/pdf/developing-and-evaluating-complex-interventions/. (Accessed 10 Aug 2018)

6. Oliver K, Kothari A, Mays N. The dark side of coproduction: do the costs outweight the benefits for health research? *Health Research Policy and Systems* 2019;17:33.

For peer review only

7. Meisinger C, Döring A, Thorand B, *et al*. Body fat distribution and risk of type 2 diabetes in the general population: are there differences between men and women? The MONICA/KORA Augsburg Cohort Study. *Am J Clin Nutr* 2006;84:483-9.

8. Bhaskaran K, Douglas I, Forbes H, *et al*. Body-mass index and risk of 22 specific cancers: a population-based cohort study of 5·24 million UK adults. *Lancet* 2014;384:755-65.

9. Zhang C, Rexrode KM, Van Dam RM, *et al*. Abdominal obesity and the risk of all-cause, cardiovascular, and cancer mortality: sixteen years of follow-up in US women. *Circ* 2008;117:1658-67.

10. Kolotkin RL, Andersen JR. A systematic review of reviews: exploring the relationship between obesity, weight loss and health-related quality of life. *Clin Obes* 2017;7:273-89.

11. Simon GE, Von Korff M, Saunders K, *et al*. Association between obesity and psychiatric disorders in the us adult population. *Arch Gen Psychiatry* 2006;63(7):824-30.

12. Public Health England. *Health matters: obesity and the food environment environment.* UK: Public Health England, 2017. https://www.gov.uk/government/publications/health-matters-obesity-and-the-food-environment/health-matters-obesity-and-the-food-environment--2. (Accessed 10 Aug 2018)

For peer review only

For peer review only - http://bmjopen.bmj.com/site/about/guidelines.xhtml

13. World Health Organisation. *Global status report on noncommunicable diseases 2014.* Geneva: World Health Organisation, 2014. https://apps.who.int/iris/bitstream/handle/10665/148114/9789241564854_eng.pdf;jsessionid=A043D0E63AACA67752CC3294A1EBD7A3?sequence=1. (Accessed 04 Feb 2019)

14. Bambra C, Hillier F, Cairns J, *et al*. How effective are interventions at reducing socioeconomic inequalities in obesity among children and adults? Two systematic reviews. *Public Health Res* 2015;3.

15. Bull ER, Dombrowski SU, McCleary N, *et al*. Are interventions for low-income groups effective in changing healthy eating, physical activity and smoking behaviours? A systematic review and meta-analysis. *BMJ Open*. 2014;4:e006046.

16. Office for National Statistics. *Dataset: Regional gross value added (balanced) by combined authority in the UK.* UK: Office for National Statistics, 2017. https://www.ons.gov.uk/economy/grossvalueaddedgva/datasets/regionalgrossvalueaddedbalancedbycombinedauthorityintheuk. (Accessed 10 Aug 2018)

17. Public Health England. *Work, worklessness and health: local infographic tool*. UK: Public Health England, 2018. https://www.gov.uk/government/publications/work-worklessness-and-health-local-infographic-tool. (Accessed 10 Aug 2018)

18. Northumberland County Council. *Public Health Service Statement 2017/18.* Northumberland: Northumberland County Council, 2017. https://northumberland.gov.uk/NorthumberlandCountyCouncil/media/About-the-

For peer review only

For peer review only - http://bmjopen.bmj.com/site/about/guidelines.xhtml

Council/Performance/Service%20Statements%202016/2017-Public-Health-Service-Statement-2017-18.pdf. (Accessed 10 Aug 2018)

19. Public Health England. *Local Authority Health Profiles*. UK: Public Health England, 2019. https://fingertips.phe.org.uk/profile/health-profiles/data#page/0/gid/1938132701/pat/6/par/E12000001/ati/102/are/E06000057/iid/10101/age/169/sex/4. (Accessed 01 Feb 2019)

20. Lister C, Payne H, Hanson CL, *et al*. The Public Health Innovation Model: Merging Private Sector Processes with Public Health Strengths. *Front Public Health* 2017;5:192.

21. Parry GJ, Carson-Stevens A, Luff DF, *et al*. Recommendations for evaluation of health care improvement initiatives. *Acad Pediatr* 2013 Nov-Dec;13(6 Suppl):S23-30.

22. Hawkins J, Madden K, Fletcher A, *et al*. Development of a framework for the co-production and prototyping of public health interventions. *BMC Public Health* 2017;17:689.

23. Stevens VJ, Funk KL, Brantley PJ, *et al*. Design and implementation of an interactive website to support long-term maintenance of weight loss. *J Med Internet Res* 2008;10(1):e1.

24. Discover Momenta Ltd. *Momenta.* UK: Discover Momenta Ltd, 2013: http://discovermomenta.com/. (Accessed 10 Aug 2018)

For peer review only

25. Hanson CL. *Advancing understanding of effective exercise on referral: a mixed methods evaluation of the Northumberland scheme*. PhD Thesis, Durham University, 2017. http://etheses.dur.ac.uk/12162/. (Accessed 10 Aug 2018)

26. Hanson CL, Allin LJ, Ellis JG, *et al*. An evaluation of the efficacy of the exercise on referral scheme in Northumberland, UK: association with physical activity and predictors of engagement. A naturalistic observation study. *BMJ Open* 2013;3:e002849

27. Sacher PM, Kolotourou M, Chadwick PM, *et al*. Randomized Controlled Trial of the MEND Program: A Family-based Community Intervention for Childhood Obesity. *Obes* 2012;18:S62-S8.

28. Leech N, Onwuegbuzie A. A typology of mixed methods research designs. *Qual Quant* 2009;43:265-75.

29. Marfell-Jones M, Olds T, Stewart A, *et al*. *International Standards for Anthropometric Assessment (2007)*. Potchefstroom, South Africa: International Society for the Advancement of Kinathropometry, 2007.

30. Tong A, Sainsbury P, Craig J. Consolidated criteria for reporting qualitative research (COREQ): a 32-item checklist for interviews and focus groups. Int J Qual. Health Care 2007;19:349–57

For peer review only

31. World Health Organisation. *BMI Classification 2006.* Geneva: World Health Organisation, 2006. http://apps.who.int/bmi/index.jsp?introPage=intro_3.html. (Accessed 10 Aug 2018)

32. Clarke A, Friede T, Putz R, *et al*. Warwick-Edinburgh Mental Well-being Scale (WEMWBS): Validated for teenage school students in England and Scotland. A mixed methods assessment. *BMC Public Health* 2011;11:487.

33. Zigmond AS, Snaith RP. The Hospital Anxiety and Depression Scale. *Acta Psychiatr Scand* 1983;67:361-70.

34. Braun V, Clarke V. Using thematic analysis in psychology. *Qual Res Psychol* 2006;3:77-101.

35. Puhan MA, Frey M, Buchi S et al. The minimal important difference of the hospital anxiety and depression scale in patients with chronic obstructive pulmonary disease. Health and Quality of Life Outcomes 2008;6:46:10.1186/1477-7525-6-46

36. Flodgren G, Goncalves BDC, Summerbell CD. Interventions to change the behaviour of health professionals and the organisation of care to promote weight reduction in children and adults with overweight or obesity. *Cochrane Database of Syst Rev* 2017;11:CD000984.

37. Phelan SM, Burgess DJ, Yeazel MW, *et al*. Impact of weight bias and stigma on quality of care and outcomes for patients with obesity. *Obes Rev* 2015;16:319-26.

For peer review only

For peer review only - http://bmjopen.bmj.com/site/about/guidelines.xhtml

38. Logue J, Allardice G, Gillies M, *et al*. Outcomes of a specialist weight management programme in the UK National Health Service: prospective study of 1838 patients. *BMJ Open* 2014;4:e003747.

39. Lebrun LA, Chowdhury J, Sripipatana A, *et al*. Overweight/obesity and weight-related treatment among patients in U.S. federally supported health centers. *Obes Res Clin Pract* 2013;7:e377-e90.

40. Ahern AL, Aveyard P, Boyland EJ, *et al*. Inequalities in the uptake of weight management interventions in a pragmatic trial: an observational study in primary care. *Br J Gen Pract* 2016;66(645):e258-63.

41. Adams J, Mytton O, White M, *et al*. Why Are Some Population Interventions for Diet and Obesity More Equitable and Effective Than Others? The Role of Individual Agency. *PLoS Med* 2016;13(4).

42. Mastellos N, Gunn LH, Felix LM, *et al*. Transtheoretical model stages of change for dietary and physical exercise modification in weight loss management for overweight and obese adults. *Cochrane Database Syst Rev* 2014;2:CD008066

43. Garrow JS, Summerbell CD. Meta-analysis: effect of exercise, with or without dieting, on the body composition of overweight subjects. *Eur J Clin Nutr* 1995;49:1-10.

44. Wright F, Boyle S, Baxter K, *et al*. Understanding the relationship between weight loss, emotional well-being and health-related quality of life in patients attending a specialist obesity weight management service. *J Health Psychol* 2013;18:574-86.

For peer review only

For peer review only - http://bmjopen.bmj.com/site/about/guidelines.xhtml

45. Somerville R, McKenzie K, Eslami S, *et al*. Poor mental health in severely obese patients is not explained by the presence of comorbidities. *Clin Obes* 2014;5:12-21.

46. Madigan CD, Daley AJ, Lewis AL, et al. Which weight-loss programmes are as effective as Weight Watchers®? Brit J Gen Pract 2014;64(620):e128-e136.

47. Curtis S, Oven K, Wistow J, *et al*. Adaptation to extreme weather events in complex health and social care systems: The example of older people's services in England. *Environ Plann C: Politics and Space* 2018;36:67-91.

48. Milat AJ, King L, Newson R, *et al*. Increasing the scale and adoption of population health interventions: experiences and perspectives of policy makers, practitioners, and researchers. *Health Res Policy and Systems* 2014;12:18.

49. Jebb S. *Dusting off Foresight's obesity report.* Foresight Blog UK: Government Office for Science, 2017. https://foresightprojects.blog.gov.uk/2017/10/04/dusting-off-foresights-obesity-report/. (Accessed 01 Feb 2019)

50. Wolfenden L, Goldman S, Stacey FG *et al*. Strategies to improve the implementation of workplace-based policies or practices targeting tobacco, alcohol, diet, physical activity and obesity. *Cochrane Database of Syst Rev* 2018;11:CD012439.

For peer review only - http://bmjopen.bmj.com/site/about/guidelines.xhtml

SUPPLEMENTARY FILE 1. Momenta session content

Week	Key topic		Key topic
**Getting started session**	Motivation for weight loss<br>Weight loss goals<br>Differences from other weight management programmes<br>Monitoring		
**Week 1**	**Snacking**<br>Fatty, sugary snacks<br>Calories from snacking<br>Healthy snack choices<br>Healthy eating patterns	**Week 7**	**Fats**<br>Reducing fat<br>Different types of fat<br>Lower-fat cooking techniques<br>Hidden fats
**Week 2**	**Heart rate**<br>Cardiovascular exercise and health<br>Cardiovascular exercise and weight management<br>Recommended amounts of CV activity<br>Heart rate and exercise	**Week 8**	**Internal triggers**<br>Introduction to internal triggers<br>Identifying internal triggers<br>Managing internal triggers
**Week 3**	**Food as fuel**<br>A balanced diet and health<br>Planning meals<br>Fibre	**Week 9**	**Active lifestyles**<br>Physical activity and health<br>Physical activity and weight management<br>Different types of physical activity
**Week 4**	**External triggers**<br>Introduction to external triggers<br>External triggers and over-eating<br>Managing triggers	**Week 10**	**Meals**<br>Positive meal environment<br>Shopping<br>Food labels
**Week 5**	**Strength**<br>Resistance activity and health<br>Resistance activity and weight management	**Week 11**	**Sugars**<br>Sugary foods and drinks and weight loss<br>Alcohol<br>Added sugars<br>Sugary drinks and appetite regulation
**Week 6**	**Breakfast**<br>Eating breakfast<br>Retraining appetite<br>Barriers to eating breakfast<br>Developing new breakfast habits<br>Beyond marketing	**Week 12**	**Eating out**<br>Challenges when eating away from home<br>Management and coping strategies<br>Moving forwards

For peer review only

SUPPLEMENTARY FILE 2.

Healthcare professionals' semi-structured interview guide

Semi Structured Interview Set the interviewee at ease; explain purpose of the interview; offer a better understanding of what the referral process requires to aid tier 2 weight management to be delivered in Northumberland; explanation about how the interview will be recorded; reaffirmation of consent; and how the information will be analysed and stored; rules of confidentiality / anonymity etc.

Questions:

1. Thinking about raising the weight issue, tell me about your experience of discussing weight with patients.

   Prompts

   - *How does it feel to raise weight as an issue?*
   - *Are patients open to discussing weight problems?*
   - *Do you find a difference between genders when discussing weight?*
   - *What helps you, such as the NHS Health Check Programme, to raise the issue of weight?*
   - *What else would help to raise the issue or weight in appointments?*

2. Greater retention is often achieved when patients are ready to change, tell me how you work with / assess patient's readiness to change.

   Prompts

   - *Have you had training around the cycle of change?*
   - *Do you use any specific tools or resources to assess the patient?*
   - *What would help you to assess the patient's readiness to change?*

For peer review only

3. Thinking the information and resources available to you during the referral, do you feel you had enough information and resources to encourage patient take up of the programme?

Prompts

- *Did you have enough background information?*
- *Were the referral forms suitable / capture all the information required?*
- *Were the patient leaflets / resources suitable?*
- *Were there questions or issues raised that couldn't be answered?*
- *Was the process easy to use?*
- *What else could help you to make referrals to weight management programmes?*

4. Thinking about after you referred the patient, what happened next? (excluded after pilot)

Prompts

- *Did you get feedback from the weight management programme on the progress of your patient?*
- *Did your patients achieve weight loss?*
- *Did your patient come back and talk about their experience?*

5. What things are most likely to prevent you from making the referral a weight management programme, either commercial or Public Health funded?

Prompts

- *Are there barriers that you perceive, such as cost to the patient?*
- *Are you concerned with raising the weight issue?*
- *Is it a time factor if the patient has an appointment for anything other than a weight issue?*
- *What would help you to overcome the barriers that prevent you from making the referral?*

For peer review only

6. Is there anything else that you would like to tell me about your expectations and experiences of the weight management programme?

Focus Group Topic Guide

Set group at ease; explain purpose of the focus group; offer a better understanding of what works for people in terms of tier 2 weight management and what doesn't, aiding development of an effective programme for Northumberland residents; explanation about how the focus group will be recorded; reaffirmation of consent; and how the information will be analysed and stored; rules of confidentiality / anonymity etc.

1. Tell me a bit about what sort of weight management activity you have taken part in, in the past.

   Prompts

   - *What influence have others had on your weight management?*
   - *Do you have any particular likes/dislikes of physical activity/managing weight/nutrition*
   - *Has there been anything else that has influenced your management of weight?*

2. So thinking about the weight management programme you have undertaken, how did you find out about it?

   Prompts

   - *Who / what motivated you to attend?*
   - *What made you decide that this is the right time to look at managing your weight?*
   - *Did the time of year make a difference?*

For peer review only

3. Thinking about your experience of when you were referred to the weight management programme, how did you find the process?

Prompts

- *What type of health professional referred you? (GP / Practice Nurse)*
- *Did you specifically attend Primary Care to discuss your weight?*
- *How was weight raised?*
- *What did the referrer explain to you about the programme? Did you get enough information?*
- *How long was it from your referral from Primary Care to the first assessment in the weight management programme; was this what you expected? Were you still motivated?*

4. How did you feel about being referred?

- *Prompts*
- *How confident did you feel about taking part in the programme?*
- *Was there anything that you were particularly looking forward to?*
- *Was there anything that you were worried about?*

5. What did you hope to achieve by taking part in the weight management programme?

Prompts

- *What were your expectations when you start attending the scheme?*
- *Have there been changes to your health that you expected happen as a result of participation?*
- *How quickly did you expect to see these changes? And did this happen?*

6. Thinking about after you were referred, what happened next?

Prompts

For peer review only

- *How long after referral did it take to be contacted by the Active Northumberland?*
- *What information did you receive prior to the initial consultation?*
- *How comfortable did you feel coming to the initial consultation?*

7. What influenced you most to attend the weight management programme?

Prompts

- *What did you expect from the staff?*
- *How important to you were changes in health or weight?*
- *Why were the influences raised important?*

8. What things were most likely to prevent you from attending the programme?

Prompts

- *Tell me about any worries you might have had about health issues.*
- *Tell me about any other things, such as other commitments, that might have stopped you from attending*
- *Did any of these issues arise? How did you overcome these issues?*

9. Now that you have completed the programme, tell me how did you felt about undertaking the weight management programme?

Prompts

- *Did you achieve the health / weight outcomes you expected?*
- *Why do you think it worked or not for you?*
- *Do you feel you now have the tools to continue to make positive lifestyle choices?*
- *Is there something that will prevent you to continue to make positive lifestyle choices?*

For peer review only

10. Is there anything else that you would like to tell me about your expectations and experiences of the weight management programme?

For peer review only

COREQ GUIDELINES REPORTING CHECKLIST: Prototyping for public health in a local context: a streamlined evaluation of a community-based weight management programme (Momenta), Northumberland, UK

No	Item	Guide questions/description	Information	Reported in manuscript (Section, page no.)
	**Domain 1: Research team and reflexivity**			
	Personal Characteristics			
1	Interviewer/ facilitator:	Which author/s conducted the interview or focus group?	LN	Qualitative evaluation component, page 10
2	Credentials	What were the researcher's credentials?	LN: part of her Public Health Masters degree	Qualitative evaluation component, page 10
3	Occupation	What was their occupation at the time of the study?	LN: Masters student and employed as a member of the Northumberland public health team at the time of the evaluation.	Qualitative evaluation component, page 10
4	Gender	Was the researcher male or female?	Female (referred to as her)	Qualitative evaluation component, page 10
5	Experience and training	What experience or training did the researcher have?	LN: Masters in Public Health (which contained qualitative methods training), mentored by TF, an experienced qualitative researcher.	Qualitative evaluation component, page 10
	Relationship with participants			
6	Relationship established	Was a relationship established prior to study commencement?	No. Practice managers from all six referring surgeries were sent an invitation for staff to take part.	Qualitative evaluation component, page 10
7	Participant knowledge of the interviewer	What did the participants know about the researcher? *e.g. personal goals, reasons for doing the research*	LN informed HCP participants about her employment status and that the study aimed to understand implementation issues.<br><br>Programme participants were invited to participate in a series of focus groups at programme-end to explore experiences	Qualitative evaluation component, page 10-11
8	Interviewer characteristics	What characteristics were reported about the interviewer/facilitator? *e.g. Bias, assumptions, reasons and interests in the research topic*	Not discussed	N/A

For peer review only

	**Domain 1: Study design**			
	Theoretical framework			
9	Methodological orientation and Theory	What methodological orientation was stated to underpin the study? *e.g. grounded theory, discourse analysis, ethnography, phenomenology, content analysis*	Qualitative data were audio-recorded and transcribed by LN using a thematic process	Data analysis, page 12
	Participant selection			
10	Sampling	How were participants selected? *e.g. purposive, convenience, consecutive, snowball*	All HCPs involved were invited: Practice managers from all six referring surgeries were sent an invitation for staff to take part<br><br>Programme participants: for the first wave of referrals, all (n = 39) were invited to participate in a series of focus groups.	Qualitative evaluation component, page 10-11
11	Method of approach	How were participants approached? *e.g. face-to-face, telephone, mail, email*	HCPs: by email<br><br>Programme participants: written information handed out during the first session	Qualitative evaluation component, page 10-11
12	Sample size	How many participants were in the study?	5 HCPs<br>13 Intervention participants	Results, page 18<br>Results, page 18
13	Non-participation	How many people refused to participate or dropped out? Reasons?	HCPs: 84 invited, 5 participated.<br><br>Programme participants: 39 invited, 13 participated<br>Reasons for refusal not documented	Qualitative evaluation component, page 10-11<br>and<br>Results, page 15<br>Results, page 18
	Setting			
14	Setting of data collection	Where was the data collected? *e.g. home, clinic, workplace*	HCPs: in referring surgeries<br><br>Programme participants: in the leisure centres	Results, page 15<br><br>Results, page 18
15	Presence of non-participants	Was anyone else present besides the participants and researchers?	Not stated	N/A
16	Description of sample	What are the important characteristics of the	HCPs: role reported	Results, page 15<br><br>Results, page 18

For peer review only

		sample? *e.g. demographic data, date*	Programme participants: programme group and weight loss status reported<br><br>Date range for interviews reported	Qualitative evaluation component, page 10
	Data collection			
17	Interview guide	Were questions, prompts, guides provided by the authors? Was it pilot tested?	Semi-structured interview guide used. Pilot tested.<br><br>Guides provided as supplementary file	Qualitative evaluation component, page 10-11<br><br>Supplementary file 2
18	Repeat interviews	Were repeat interviews carried out? If yes, how many?	No not applicable to study design	N/A
19	Audio/visual recording	Did the research use audio or visual recording to collect the data?	Yes, the interviews were audio recorded	Data analysis, page 12
20	Field notes	Were field notes made during and/or after the interview or focus group?	Notes taken from focus groups helped to contextualise developing themes	Data analysis, page 12
21	Duration	What was the duration of the interviews or focus groups?	HCPs average length reported: 26 minutes<br><br>Programme participants: range reported: 26-44 minutes	Qualitative evaluation component, page 10<br><br>Qualitative evaluation component, page 11
22	Data saturation	Was data saturation discussed?	No	N/A
23	Transcripts returned	Were transcripts returned to participants for comment and/or correction?	No	N/A
	**Domain 3: analysis and findings**			
	Data analysis			
24	Number of data coders	How many data coders coded the data?	N=2 (LN and TF)	Data analysis, page 12
25	Description of the coding tree	Did authors provide a description of the coding tree?	Yes key themes described at beginning of HCP qualitative results section and at beginning of programme participant qualitative results section	Results, page 15<br><br>Results, page 18
26	Derivation of themes	Were themes identified in advance or derived from the data?	Identified from data	Data analysis, page 12

For peer review only

27	Software	What software, if applicable, was used to manage the data?	Not stated	N/A
28	Participant checking	Did participants provide feedback on the findings?	No	N/A
	Reporting			
29	Quotations presented	Were participant quotations presented to illustrate the themes / findings? Was each quotation identified? e.g. participant number	Yes, participants identified using a participant label	Results, page 15-19
30	Data and findings consistent	Was there consistency between the data presented and the findings?	Themes were illustrated by participant quotations	Results, page 15-19
31	Clarity of major themes	Were major themes clearly presented in the findings?	Themes identified and presented under sub headings for both HCPs and programme participants	Results, page 15-19
32	Clarity of minor themes	Is there a description of diverse cases or discussion of minor themes?	For HCPs minor themes highlighted under additional barriers to engagement	Results, page 17

For peer review only

BMJ Open

Prototyping for public health in a local context: a streamlined evaluation of a community-based weight management programme (Momenta), Northumberland, UK

Journal:	*BMJ Open*
Manuscript ID	bmjopen-2019-029718.R3
Article Type:	Original research
Date Submitted by the Author:	05-Sep-2019
Complete List of Authors:	Dodd-Reynolds, Caroline; Durham University, Department of Sport and Exercise Sciences; Durham University, Wolfson Research Institute for Health and Wellbeing Physical Activity Special Interest Group<br>Nevens, Lisa; Northumbria Healthcare NHS Foundation Trust Community Services Business Unit<br>Oliver, Emily; Durham University, Wolfson Research Institute for Health and Wellbeing Physical Activity Special Interest Group; Durham University, Deaprtment of Sport and Exercise Sciences,<br>Finch, Tracy; Northumbria University, Nursing, Midwifery and Health<br>Lake, Amelia; Department of Science, School of Science, Engineering and Design, Teesside University<br>Hanson, Coral; Edinburgh Napier University, School of Health and Social Care; Durham University, Wolfson Research Institute for Health and Wellbeing Physical Activity Special Interest Group
**Primary Subject Heading**:	Public health
Secondary Subject Heading:	Nutrition and metabolism
Keywords:	public health evaluation, prototyping, implementation, community weight management, NUTRITION & DIETETICS, exercise referral

SCHOLARONE™
Manuscripts

Prototyping for public health in a local context: a streamlined evaluation of a community-based weight management programme (Momenta), Northumberland, UK

Caroline J Dodd-Reynolds[1,2] (corresponding author*), Lisa Nevens[3], Emily J Oliver[1,2], Tracy Finch[4], Amelia A Lake[56] Coral L Hanson[7]

[1]Department of Sport and Exercise Sciences, Durham University, Durham, DH1 3HN, UK*.
Email: caroline.dodd-reynolds@durham.ac.uk Tel: +44(0)191 3342000
[2]Wolfson Research Institute for Health and Wellbeing Physical Activity Special Interest Group, Durham University, Durham, UK.
[3]Northumbria Healthcare NHS Foundation Trust, Northumberland, UK.
[4]Department of Nursing, Midwifery and Health, Northumbria University, Newcastle upon Tyne, UK
[5]Department of Science, School of Science, Engineering and Design, Teesside University, Teesside, UK
[6]Fuse – UKCRC Centre for Translational Research in Public Health, UK
[7]School of Health and Social Care, Edinburgh Napier University, Edinburgh, UK

Word count: 4214

Keywords: Public health evaluation, protoyping, implementation, community weight management, diet, exercise referral

For peer review only - http://bmjopen.bmj.com/site/about/guidelines.xhtml

ABSTRACT

Objectives Stakeholder co-production in design of public health programmes may reduce the 'implementation gap' but can be time-consuming and costly. Prototyping, iterative refining relevant to delivery context, offers a potential solution. This evaluation explored implementation and lessons learned for a 12-week referral-based weight-management programme, 'Momenta', along with feasibility of an iterative prototyping evaluation framework.

Design Mixed methods evaluation: qualitative implementation exploration with referrers and service users; preliminary analysis of anonymised quantitative service data (12 and 52 weeks).

Setting Two leisure centres in Northumberland, northeast England.

Participants Individual interviews with referring professionals (n=5) and focus groups with service users (n=13). Individuals (n=182) referred by healthcare professionals (quantitative data).

Interventions Three 12-week programme iterations: Momenta (n=59), Momenta-Fitness membership (Momenta-Fitness (n=58), and Fitness membership only (n=65).

Primary and secondary outcome measures Primary outcome: Qualitative themes developed through stakeholder-engagement. Secondary outcomes included preliminary exploration of recruitment, uptake, retention, and changes in weight, BMI waist circumference and psychological wellbeing.

Results Service users reported positive experiences of Momenta. Implementation gaps were revealed around the referral process and practitioner knowledge. Prototyping enabled

For peer review only - http://bmjopen.bmj.com/site/about/guidelines.xhtml

iterative refinements such as broadening inclusion criteria. Uptake and 12-week retention were higher for Momenta (84.7%, 45.8%) and Momenta-Fitness (93.1%, 60.3%) versus Fitness-only (75.4%, 24.6%). Exploration of other preliminary outcomes (completers only) suggested potential for within-group weight loss and increased psychological wellbeing for Momenta and Momenta-Fitness at 12 weeks. 52-week follow-up data were limited (32%, 33%, and 6% retention for those who started Momenta, Momenta-Fitness and Fitness respectively) but suggested potential weight loss maintenance in Momenta-Fitness.

Conclusions Identification of issues within the referral process enabled real-time iterative refinement, whilst lessons learned may be of value for local implementation of 'off-the-shelf' weight management packages more generally. Our preliminary data for completers suggest Momenta may have potential for weight loss, particularly when offered with a fitness membership.

For peer review only - http://bmjopen.bmj.com/site/about/guidelines.xhtml

ARTICLE SUMMARY

Strengths and limitations of this study

- This study advances understanding about whether prototyping is a time-efficient and cost-effective approach to design and implementation of public health programmes.
- This mixed methods evaluation provides insight into the implementation of an 'off-the-shelf' weight management programme, in a local context.
- Embedding stakeholders' views throughout the entire evaluation process allowed for ongoing, iterative refinement.
- A limitation to the quantitative component is the small sample size and rate of missing data at 1 year; findings should thus be interpreted with caution.
- Qualitative interviews and focus groups can only provide information about what participants recall about their experiences, meaning that there is a potential for recall bias.

For peer review only

For peer review only - http://bmjopen.bmj.com/site/about/guidelines.xhtml

INTRODUCTION

Failure to implement effective public health interventions when programmes are scaled up or transferred across contexts is widely reported.[1] Proposed approaches attempting to address this implementation gap include; effectiveness-implementation hybrid designs,[2] linking action to theory and models based on theory,[3] and application of the replicating effective programmes framework.[4] Common to all is advocacy of a developmental process reflecting on existing knowledge about the target population and planned programme prior to service delivery. Furthermore, engagement of service users is encouraged at all stages of intervention and evaluation design in MRC guidance.[5] Although this increases the likelihood of services meeting all stakeholder needs, concerns about the practical, personal, and professional costs of co-production have been raised.[6] Resulting well-designed services will be tailored to a problem that may have changed during the time spent developing the intervention. Additionally, public access may be delayed. Resource-pressured public health services must therefore consider pragmatic alternatives to service design and implementation. In this paper, we explore a novel evaluation approach to these implementation challenges, focusing on a problem high on the public health agenda: obesity and overweight.

Targeting elevated weight status is a public health priority, obesity being a recognised risk factor for many negative physical and psychological health outcomes.[7-11] In England for example, obesity and overweight are associated with 30,000 deaths and an estimated National Health Service cost of £6.1 billion per annum.[12] Globally, countries with higher income inequalities tend to have higher rates of obesity.[13] Excess weight is also associated with widening social and economic deprivation,[14] with calls to improve the effectiveness of behaviour change interventions for low-income groups.[15] There is a clear need for effective

For peer review only - http://bmjopen.bmj.com/site/about/guidelines.xhtml

public health programmes that can be refined according to local need, especially in areas with substantial deprivation. This evaluation focuses on Northumberland, in northeast England. Northumberland is one of the lowest ranked counties in England by Gross Value Added per capita (£16,140).[16] Unemployment is higher (5.5% versus 4.8%) than the England average[17] and Northumberland public health spend per person is £53, compared to a £59 national average.[18] 63.8% of adults are classified as having excess weight, higher than the national average of 61.3%.[19]

The need for innovation within public health has been postulated, shifting away from the traditional linear pre-conceived and evidence-based model.[20] For example, Parry and colleagues[21] call for research to explore not only how a programme works, but also the context and requirements for any adaptations. One such approach is prototyping[22] where projects test innovations iteratively, with ongoing refinement considering the interplay between a programme and its delivery context. Evaluation and public health teams are able to communicate at all stages of the programme, with evaluation recommendations incorporated via a rapid-cycle basis.[21] A small number of studies to date, for example in drug prevention[22] and web-based support of long-term weight loss[23] have demonstrated efficiencies when including elements of prototyping within programme development (including time, adaptation to context and cost). Such an approach seems particularly well-suited to weight management, where there are many examples of 'good' practice, or effectiveness, but no clear consensus on 'best' practice at service-delivery level. There is also limited understanding of how 'scaling up' and adapting of programmes or interventions to local contexts may impact on effectiveness. This evaluation has particular value therefore in testing a prototyping approach for a weight management programme, delivered and adapted 'in real-time', at local authority level.

For peer review only - http://bmjopen.bmj.com/site/about/guidelines.xhtml

The aim was to explore implementation of an 'off-the-shelf' weight management programme, Momenta[24], in a challenging context. Specific objectives were to explore local implementation, consider feasibility of the iterative prototyping evaluation framework and explore preliminary outcome domains including recruitment, retention, weight change and wellbeing.

METHODS

The prototyping process: local context and evaluation design

A local authority health needs assessment identified a gap in provision for a lifestyle-based weight management referral programme within Northumberland. Adults with overweight or obesity were at the time eligible for referral to the Northumberland exercise referral scheme (ERS), however previous evaluation demonstrated modest weight loss[25] and body mass index (BMI) >30 kg/m^2 was negatively associated with adherence.[26] Thus 'Momenta' was commissioned for local adaptation and delivery. The Momenta programme incorporates evidence-based behaviour change techniques and is designed to be delivered by fitness professionals in a leisure environment.[24] Developed by the MEND childhood weight management programme[27] designers, this 12-week programme aims to facilitate weight loss by engaging participants in 12 key behaviours broadly encompassing psychology, diet and physical activity (supplementary file 1). Briefly, Momenta sessions explored topics using interactive and experiential learning techniques including brainstorming, group activities and discussion, quizzes and games. At the end of each session, participants set goals focusing on one of the 12 key behaviours. At the beginning of each session, the group discussed the

For peer review only - http://bmjopen.bmj.com/site/about/guidelines.xhtml

previous weeks' goals by exchanging stories and brainstorming challenges. All interventions were free to service users.

The local Leisure Trust was commissioned to deliver a pilot Momenta programme. Commissioners and providers had ideas about alternative delivery options and due to an established academic relationship, asked the study team for advice about robust evaluation that would allow for feedback in real time and at the end of the pilot. Stakeholder meetings were held with Public Health staff (n=2), Leisure Trust managers (n=3), delivery staff (n=2) and Momenta programme developers (n=2). As part of the prototyping process, members of the evaluation team (CDR, EO) provided guidance on evaluation design and light touch advice about tools to explore preliminary effectiveness. The evaluation was thus co-produced to ensure a robust framework, whilst meeting strategic local needs. For example, commissioners were concerned about meeting recruitment targets for an existing specialist weight management service used mainly for pre-bariatric patients and Momenta was initially commissioned for patients with BMI 25.0-29.9 kg/m^2, although this was later amended. Furthermore, commissioners were keen to consider accessibility of provision and wished to explore offering free gym, swimming and fitness class membership. The evaluation was designed to accommodate this.

The programme was ultimately delivered at two leisure sites situated within the 20% and 50% most deprived neighbourhoods in the country. Six General Practice (GP) surgeries, identified as the best referrers to the existing ERS, were asked to refer suitable patients to Momenta. The programme manager and the public health improvement manager (LN) attended practice meetings to articulate referral criteria and disseminate advertising materials.

For peer review only

Attendance varied from two to all practice staff, meaning that in some surgeries knowledge of the programme was reliant on dissemination by those who attended.

A mixed methods evaluation was agreed between the evaluation team and commissioners. Qualitative and quantitative components were conducted concurrently and had equal status.[28] Prototyping allowed for iterative changes to be made to the implementation and delivery of the programme in real time and we reflect upon these in the results and discussion.

Referrals by healthcare professionals (HCPs) were via a standardised form to the appropriate leisure site. Prior to programme commencement the Leisure Trust, in conjunction with the Momenta programme designer and members of the evaluation team, held a training day for delivery staff. Although staff were qualified to deliver Momenta, extra bespoke training (including role-play scenarios and problem-solving discussions) was delivered by the clinical psychologist who designed Momenta. The evaluation team (CDR, CH) trained delivery staff in international standard anthropometric techniques[29] and familiarised them with other evaluation measures.

Programme providers allocated service users into one of three comparison groups:
a) Combined Momenta plus fitness membership (Momenta-Fitness);
b) Momenta;
c) Fitness membership (Fitness only).
Participants were allocated into groups in order of receipt (the first referral form received was allocated to Momenta-Fitness, the second form to Momenta, the third form to fitness only etc.). The provider then contacted participants by telephone to arrange attendance. If a participant was unable to attend the allocated group, (e.g. due to inconvenient session times)

For peer review only

For peer review only - http://bmjopen.bmj.com/site/about/guidelines.xhtml

provider allocated them to a different group after discussion. Due to maximum recommended Momenta group size, referrals were split into delivery cohorts of 15, with groups rolling through March 2015 to April 2016.

Implementation effectiveness for the referral process was explored through semi-structured interviews with referring HCPs (undertaken at referring surgeries) and focus groups with service users (in leisure centres). All were conducted by LN during March-July 2015, as part of her Public Health Master's degree (which contained qualitative methods training), mentored by TF, an experienced qualitative researcher. LN was employed as a member of the Northumberland public health team at the time of the evaluation. Questions are included in supplementary file 2. Data were audio-recorded. Results are reported using the Consolidated criteria for Reporting Qualitative research guidelines.[30]

Practice managers from all six referring surgeries were sent an email invitation for staff to take part (n = 84), (General Practitioner = 53, Practice Nurse = 18, Health Care Assistant = 13). Individual correspondence was sent to those agreeing. LN informed participants about her employment status and that the study aimed to understand implementation issues. Interviews aimed to explore HCPs' referral experiences; raising weight issues; assessing readiness to change; marketing and referral materials; and the referral process. Interview questions were pilot tested with public health colleagues to assess timing and ensure validity. One question (*Thinking about after you referred the patient, what happened next?*) was omitted after piloting as it was realised HCPs would not have had patient feedback at that point. Interviews lasted on average 26 minutes and were transcribed verbatim. Data were analysed following each interview, with developing themes considered to determine whether

For peer review only - http://bmjopen.bmj.com/site/about/guidelines.xhtml

questions required refinement. Initial themes generated from the first two interviews did not change and thus questions remained constant, although prompts were added.

During the initial assessment session for the first wave of referrals, all (n = 39) were given a written invitation to participate in a series of focus groups at programme-end to explore experiences. Emphasis was placed on the referral process, initial expectations and experiences of participation; how weight issues were raised by HCPs; time from referral to initial assessment; and facilitators and barriers to taking part. Focus groups lasted between 26 and 44 minutes.

Preliminary outcome data were collected to provide an initial indication of programme success. These included anthropometric measurements to determine weight change. Well-being measures were of specific interest to commissioners. Sociodemographic information was also available as indicated.

Age, gender and postcode (for index of multiple deprivation, IMD) were recorded by referring HCPs on the referral form. Employment status, level of education, cohort wave and programme group were recorded by leisure staff, who also measured weight and stature (without shoes or bulky clothing) and waist circumference at baseline and programme end. Measures were taken in at least duplicate, using standardised tools in accordance with international standards[29] using SECA 761 scales, a Leicester portable stadiometer and anthropometry tape. Body mass index was calculated and classified according to WHO guidelines.[31] The Warwick-Edinburgh Mental Well-being Scale,[32] and the Hospital Anxiety and Depression Scale (HADS)[33] were administered at each time-point. Attendance at Momenta and leisure centre usage was monitored via swipe-card tracking. 52 weeks after

For peer review only - http://bmjopen.bmj.com/site/about/guidelines.xhtml

commencing the programme, service users were invited to attend a follow-up session, where leisure staff repeated physiological and psychological measures. Programme providers collected and collated quantitative data and provided an anonymised dataset to the evaluation team for analysis.

Patient and public involvement

Patients and the public were not involved in the choice of evaluation topic, assisting in the study design, advising on the project or in carrying out the evaluation.

Data analyses

Qualitative data were audio-recorded and transcribed by LN using a thematic process.[34] Data were organised according to concepts, key themes and developing categories. Data coding was discussed with TF, allowing comparison of data interpretation and subsequent coding refinement. Evolving key themes were refined through the analysis process and subsequent cross-sectional thematic labelling of data, thus generating deeper understanding. Where possible, key phrases or expressions identified from interviews and focus groups were retained within coding and thematic labelling. A public health colleague helped to verify interpretations of the data and appropriateness of codes applied. Once initial interviews were coded this framework was applied to remaining data. Notes taken during focus groups helped to contextualise when developing themes and included information about dynamics within groups, such as influence, disagreement, humour and peer exposure.

The anonymised quantitative dataset was analysed using PSAW Statistics V.22. Descriptive statistics were calculated for age, gender, IMD, employment status, initial BMI, leisure site,

For peer review only - http://bmjopen.bmj.com/site/about/guidelines.xhtml

level of education, and uptake and adherence. Distribution and normality of measures (weight, BMI, waist circumference, psychological wellbeing and attendance) were assessed using Shapiro Wilk tests and median and interquartile range (IQR) scores calculated for each group at baseline and 12 weeks (attendance, 12 weeks only). Using complete cases, Kruskal-Wallis H tests were used to explore preliminary between-group differences at baseline and at 12 weeks and Wilcoxon-signed rank tests explored preliminary repeated measures differences between baseline and 12-week scores. Complete cases available at 52 weeks (n = 37) were considered similarly, but via separate analyses due to limited available data across comparison groups.

RESULTS

Between December 2014 and March 2016, the programme received 182 referrals and was delivered in four cohorts across leisure sites. Due to initial low levels of recruitment, the first cohort did not start until March 2015. Referrals were mainly female (83%) and 30.6% lived in the 20% most deprived areas (table 1).

For peer review only - http://bmjopen.bmj.com/site/about/guidelines.xhtml

Table 1. Demographic characteristics of referrals who started the programme (n=153)

	Median	IQR
**Age (years)**	53	24
**Gender**	**n**	**%**
Male	25	16.3%
Female	120	78.4%
Not stated	8	5.2%
**Initial BMI category (kg/m^2)**		
25.0-29.9	40	26.1%
30.0-34.9	73	-47.7%
35.0-39.9	27	17.6%
≥40.0	10	6.5%
Not stated	3	2.0%
**Leisure site**		
Leisure site 1 (IMD quintile 2)	69	45.1%
Leisure site 2 (IMD quintile 3)	83	54.2%
Not stated	1	0.7%
**Index of multiple deprivation**		
20% most deprived	42	27.5%
21-40%	33	21.6%
41-60%	17	11.1%
61-80%	20	13.1%
20% least deprived	35	22.9%
Not stated	6	3.9%
**Employment status**		
Employed full time	36	23.5%
Employed part time	24	15.7%
Retired	51	33.3%
Claiming incapacity benefit	5	3.3%
Claiming job seekers allowance	6	3.9%
Not stated	14	9.2%
**Level of education**		
Primary	15	9.8%
Secondary (O level/GCSE)	35	22.9%
Secondary (A level)	26	17.0%
Further education (HND)	24	15.7%
Bachelors or equivalent	21	13.7%
Masters or equivalent	5	3.3%
Not stated	27	17.6%

Age, gender and postcode (IMD calculated by the programme provider) recorded from the referral form.

Employment and level of education self-reported by participants during the first session. The provider did not follow up missing data.

BMI and leisure site recorded by the provider. Missing data not available for analysis and presumed to be data entry errors.

For peer review only

Implementation effectiveness: reflections from referring healthcare professionals

Five face-to-face semi-structured interviews took place with HCPs across five referring surgeries: two GPs, two Practice Nurses and one Health Care Assistant. HCPs perceived that four key themes influenced the effectiveness of programme implementation: (i) difficulties raising weight with patients, (ii) how gender affected patient engagement, (iii) availability of information and resources, and (iv) additional barriers constraining programme promotion.

Raising the issue of weight with patients:

Concerns about raising weight may have contributed to slow recruitment, with nurses and healthcare assistants expressing unease, *'not really up to me… well I talk about it if they want to…. Better if they [patients] bring it up.'* (Interview 2, Healthcare Assistant). GPs seemed more comfortable raising weight with patients, but with the caveat that this is easier in the context of a longer-term GP/patient relationship.

> *'the people I see I've known for a very long time… it's the rapport you have…if I'd never met anyone before and they came in for a sore throat I'm not going to say you're fat…If there was someone I'd known for a long time and it seemed relevant…I'd mention it.'* (Interview 5, GP).

Gender and engagement in the referral process:

For peer review only - http://bmjopen.bmj.com/site/about/guidelines.xhtml

Gender was highlighted as influencing the referral process, women being more likely than men to seek support for weight. This may help explain the low rate of referral for males (17%):

'More women talk about it…men don't really talk about weight…I do mention weight to men if I'm doing a well man [sic] but it doesn't come up really…it's a woman thing…' (Interview 3, Practice Nurse).

Availability of information and resources:

Several interviewees highlighted training needs around programme information and resources, (e.g., additional programme information would help to engage patients). For example, the GPs both discussed the longstanding ERS and stated they needed to become more familiar with Momenta, as they had with the ERS:

'when we get opportunities to do things in the practice we normally discuss it, let everyone know where appropriate forms and information is and it's in your head…that didn't happen with this and I don't know why that was.' (Interview 5, GP).

All HCPs interviewed felt the referral leaflet (provided by programme providers) was important in the process, either as a tool to promote the intervention or to convey information to patients:

'The leaflet was good, bright…explained the programme and patients like taking a leaflet away.' (Interview 3, Practice Nurse)

For peer review only - http://bmjopen.bmj.com/site/about/guidelines.xhtml

For peer review only

Additional Barriers to Engagement:

Several sub-themes highlighted additional barriers to the referral process. The most prominent sub-themes were around initial BMI referral criteria (25.0-29.9 kg/m^2) and delayed programme start. Both implementation factors were beyond the control of the referrers, but consequently amended through iterative refinement during the prototyping process following early data analysis. Both were reported by practice nurses as exacerbating each other:

> '*we were referring but then it didn't start so people were not sure what was happening [pause]…Think it was more people were needed to start…but you know if the BMI was higher then there would have been more.*' (Interview 3, Practice Nurse).

In one case, a decision was taken to relax the referral criteria, *'…31.5 [kg/m^2]…was just outside so I just referred him.*' (Interview 4, GP).

Programme location was perceived by HCPs to overcome an existing barrier to the tier three weight management programme, as Momenta was '*round the corner for people,*' as opposed to '*a bit far away at the hospital.'* Cost barriers were also discussed, both with reference to the patient, *'in this sort of area…cost…, if you've got to pay it's a barrier.'* (Interview 4, GP), and to expected targets from Clinical Commissioning Groups (CCG),

> *'we are constantly told by the CCG that we must keep down on numbers and that if there are costs attached to this referral that would definitely impact… and that would be for all practices.'* (Interview 5, GP)

For peer review only - http://bmjopen.bmj.com/site/about/guidelines.xhtml

Implementation effectiveness: reflections from participants

Three focus groups in the leisure centres allowed programme participant voices to be heard: three females and one male from Momenta (focus group 1), three males and three females from Momenta-Fitness (focus group 2) and three females (one of whom emailed her views separately) from Fitness-only. Across the groups, 12 participants reported having lost weight and one reported weight gain. Three themes developed: (i) outcomes of the programme, (ii) facilitators and barriers to engagement, and (iii) raising the issues of weight with HCPs.

Outcomes of the programme:

Focus group findings aligned closely with quantitative outcomes in terms of the physical and psychological benefits of participation: *'[I've] lost a good bit of weight. It's been very positive for me… I'm feeling a lot more active…'* (Momenta-Fitness, Participant 5). Participants reported a sense of weight loss achievement, increased physical activity levels, and positive mood states. In addition, elements of the Momenta programme were perceived as facilitating engagement, including the *'group feeling… I looked forward to it,'* (Momenta-Fitness, Participant 4), the *'information that we got every week… so very well planned.'* (Momenta-Fitness, Participant 3) and the ongoing support e.g., *'she 'phoned me the other day to see if I was coming,'* (Momenta-Fitness, Participant 4). Momenta participants reflected back on, and identified and discussed lifestyle factors that related to their initial weight gain (e.g., *'I did the usual thing… I started eating toffees,'* Momenta-Fitness, Participant 5*),* demonstrating both self-awareness and an openness to discussing the topic.

Facilitators and barriers to engagement:

For peer review only - http://bmjopen.bmj.com/site/about/guidelines.xhtml

One participant reported being initially excluded but later allowed to take part, and others raised concerns that the initial BMI threshold for referral (25-29.9 kg/m^2) was too low, '*was a little bit high, BMI…managed to get it down... [and then] the doctor put us forward,'* (Momenta, Participant 2). Data also indicated the importance of subsidised access, particularly important in the context of a deprived region such as this, e.g., '*I also joined Weight Watchers for short period of time but found the classes too expensive,*' (Fitness-only, Participant 3, emailed response).

Raising the issue of weight with HCPs:

Some data did suggest implementation was problematic, however, this focused exclusively on the referral process. Participants overwhelmingly felt that they had opened the conversation about weight, as opposed to discussions being initiated by HCPs (e.g., '*my glucose levels were quite high but nobody ever said that I was overweight*,' Momenta-Fitness, Participant 4*).* In addition, participants perceived limitations in HCPs' knowledge of intervention components ('*she [nurse] didn't know anything about it*,' Fitness-only, Participant 1), something with potential to impact on likelihood of referral, and participants' expectations of programme success.

Preliminary outcome domains

Of all referrals, 153 (84%) attended the baseline measurement session and 78 (51% of those who started) attended the 12-week measurement session. Uptake and adherence varied by programme group (table 2).

Table 2. Programme uptake, adherence and attendance.

Uptake (week 1), adherence and retention (both week 12)	Momenta-Fitness		Momenta only		Fitness only	
Number referred		58		59		65
Uptake* (n, %)		54 (93.1%)		50 (84.7%)		49 (75.4%)
Uptake retention** (n, %)		35 (64.8%)		27 (54.0%)		16 (32.7%)
Uptake adherence^ (n, %)		34 (63.0%)		26 (52.0%)		8 (50.0%)
Overall retention*** (n, %)		35 (60.3%)		27 (45.8%)		16 (24.6%)
Overall adherence^^ (n, %)		34 (58.6%)		26 (44.1%)		8 (12.3%)
**Momenta session attendance**	**Momenta-Fitness**		**Momenta only**		**Fitness only**	
	**n**	**Median (IQR)**	**n**	**Median (IQR)**		
Uptake	54	9.0 (7.3)	50	9.0 (8.0)		
Dropouts	19	3.0 (3.0)	23	3.0 (5.0)		N/A
Completers^^^	35	10.0 (2.0)	27	11.0 (1.3)		
**Exercise session attendance**	**Momenta-Fitness**		**Momenta only**		**Fitness only**	
	**n**	**Median (IQR)**	**n**	**Median (IQR)**	**n**	**Median (IQR)**
Uptake	54	7.0 (16.3)	50	0.0 (4.5)	49	0.0 (1.5)
Dropouts	19	0.0 (1.0)	23	0.0 (0.0)	33	0.0 (0.0)
Completers^^^	35	10.0 (14.0)	26	0.0 (5.0)	16	4.5 (18.0)

Uptake* *participant attended baseline assessment;* ***Uptake retention**** *% of participants who attended the 12-week assessment out of those who attended the baseline assessment;* ***Uptake adherence^*** *% of participants who attended the baseline assessment who also attended ≥ 8 Momenta sessions (Momenta-Fitness and Momenta only) or gym sessions (fitness only);* ***Overall retention***** *% of all those referred who attended both baseline and 12-week assessment;* ***Overall adherence^^*** *% of all those referred who attended ≥ eight Momenta sessions (Momenta-Fitness and Momenta only) or exercise sessions (fitness only); Completers^^^ those who completed the 12-week assessment*

Physiological and psychological data were not normally distributed. Quantitative data are presented as exploratory, due to the small sample size and are presented here for information and general description. No differences were found between programme groups either at baseline or at 12 weeks, for any measures. Despite the small sample size, within-group changes between baseline and 12 weeks were evident for weight, BMI and waist circumference for Momenta-Fitness, and Momenta (Table 3). Follow-up analysis at 52-weeks (available sub-sample) suggested changes were maintained for Momenta-Fitness (n =18) only.

For peer review only - http://bmjopen.bmj.com/site/about/guidelines.xhtml

1 **Table 3. Weight, BMI and waist circumference change.**

2

End of programme results	Median (IQR) Baseline	Median (IQR) 12 weeks	z	Median (IQR) Change
**Weight (kg)**				
Momenta-Fitness (n=35)	88.9 (80.5 - 100.0)	88.0 (77.2 - 95.8)	-4.531	-2.9 (-5.1 - -1.6)
Momenta only (n=26)	87.8 (74.5 - 77.0)	83.3 (74.5 - 92.5)	-4.344	-2.9 (-5.0 - -2.0)
Fitness only (n=15)	76.2 (71.6 - 86.9)	76.6 (70.4 - 84.6)	-0.879	0.0 (-3.2 - 1.0)
**BMI (kg/m²)**				
Momenta-Fitness (n=35)	32.0 (30.3 - 35.7)	31.3 (29.2 – 35.3)	-4-494	-1.1 (-1.9 - -0.6)
Momenta only (n=26)	32.0 (30.0 - 34.5)	31.3 (28.6 – 33.6)	-4.356	-1.2 (-1.6 - -0.8)
Fitness only (n=14)	29.2 (27.3 - 33.0)	29.7 (27.0 – 33.3)	-0.454	0.1 (-1.2 - +0.4)
**Waist circumference (cm)**				
Momenta-fitness (n=35)	106.0 (98.0 - 115.0)	99.0 (93.0 - 110.0)	-4.996	-7.0 (-9.5 - -5.0)
Momenta only (n=25)	108.0 (99.5 - 114.5)	101.0 (93.8 - 111.5)	-4.166	-5.0 (-7.3 - -2.5)
Fitness only (n=11)	90.0 (87.0 – 95.0)	91.0 (90.0 - 96.0)	0.358	1.0 (-3.0 - 3.0)
**52-week follow-up**	**Median (IQR) Baseline**	**Median (IQR) 52 weeks**	**z**	**Median (IQR) Change**
**Weight (kg)**				
Momenta-Fitness (n=18)	95.2 (87.1 - 101.4)	91.4 (82.7 - 95.9)	-3.006	-4.8 (-6.2 - -1.5)
Momenta only (n=16)	84.7 (72.3 - 95.2)	82.7 (73.2 - 94.6)	-1.533	-0.7 (-7.6 - 0.8)
*Fitness only (n=3)	73.4 (69.5 - 80.2)	70.3 (66.0 - 87.0)		0.9 (-7.4 – 6.9)
**BMI (kg/m²)**				
Momenta-Fitness (n=18)	32.0 (30.49 - 35.1)	30.8 (28.7 - 34.0)	-3.157	-1.7 (-2.0 - -0.6)
Momenta only (n=16)	31.7 (29.3 – 33.9)	31.1 (26.7 – 33.6)	-1.603	-0.3 (-2.3 - 0.3)
*Fitness only (n=3)	27.6 (27.5 - 30.5)	27.8 (24.8 – 33.2)		0.3 (24.8 - 33.2)
**Waist circumference (cm)**				
Momenta-Fitness (n=18)	109.0 (101.0 - 114.8)	100.5 (94.8 - 107.3)	-3.221	-6.0 (-13.3 - -1.75)
Momenta only (n=16)	106.0 (94.5 - 115.8)	103.5 (98.5 - 113.3)	-0.780	-2.5 (-9.0 - -10.0)
*Fitness only (n=3)	89.0 (87.0 – 95.0)	90.0 (90.0 – 101.0)		3.0 (90.0 – 101.0)

** Fitness only n=3 therefore median and range reported and no statistical test completed.*

3

4 Differences in mental wellbeing, depression and anxiety were not apparent between groups, 5 however improvements in mental wellbeing, and reductions in depression and anxiety were 6 suggested between baseline and 12 weeks for Momenta-Fitness, and Momenta groups only 7 (Table 4), although the magnitude of change was similar for all groups. Sub-sample analysis 8 at 52-weeks demonstrated potential for improvements for wellbeing and depression to be 9 maintained for Momenta-Fitness (n=18), and wellbeing and anxiety for Momenta (n=16).

10

For peer review only - http://bmjopen.bmj.com/site/about/guidelines.xhtml

For peer review only

Table 4. Wellbeing, anxiety and depression measures change.

End of programme results	Median (IQR) Baseline	Median (IQR) 12 weeks	z	Median (IQR) Change
**Mental wellbeing scale**				
Momenta-Fitness (n=29)	46.0 (40.0 - 51.5)	53.0 (40.0 - 51.5)	3.810	5.0 (1.5 - 12.0)
Momenta only (n=23)	49.0 (39.0 - 58.0)	55.0 (51.0 - 63.0)	2.818	6.0 (-1.0 - 10.0)
Fitness only (n=13)	47.0 (40.5 - 59.5)	46.0 (42.0 - 63.5)	0.157	0.0 (-4.0 - 5.0)
**Anxiety scale**				
Momenta-Fitness (n=28)	5.5 (4.0 - 9.8)	4.5 (2.0 - 7.0)	-3.027	-1.0 (-3.0 - 1.0)
Momenta only (n=23)	8.0 (6.0 - 10.0)	4.0 (2.5 - 9.0)	-2.329	-1.0 (-3.0 - 0.0)
Fitness only (n=13)	8.0 (3.5 - 10.0)	6.0 (4.0 - 9.0)	-0.499	-1.0 (-2.0 - 2.0)
**Depression scale**				
Momenta-Fitness (n=28)	5.5 (3.3 - 8.0)	2.0 (1.0 - 6.0)	-3.214	-2.5 (-4.8 - -0.3)
Momenta only (n=23)	5.0 (3.0 - 7.5)	3.0 (1.0 - 5.0)	-3.049	-1.0 (-4.5 - 1.0)
Fitness only (n=13)	4.0 (2.0 - 8.5)	2.0 (2.0 - 7.0)	-1.226	-2.0 (-4.5 - 0.0)

52-week follow-up	Median (IQR) Baseline	Median (IQR) 52 weeks	z	Median (IQR) Change
**Mental wellbeing scale**				
Momenta-Fitness (n=15)	44.0 (39.0 - 52.0)	55.0 (48.0 - 59.0)	2.984	5.0 (3.0 - 15.0)
Momenta only (n=13)	58.0 (47.5 - 59.0)	56.0 (54.0 - 63.5)	2.282	4.0 (0.5 - 6.5)
*Fitness only (n=3)	47.0 (34.0 – 64.0)	58.0 (45.0 – 60.0)		-2.0 (-6.0 – 26.0)
**Anxiety scale**				
Momenta-Fitness (n=15)	6.0 (2.0 - 10.0)	2.0 (1.0 - 7.0)	-1.785	-3.0 (-6.0 - 0.0)
Momenta only (n=15)	7.0 (4.0 - 9.0)	5.0 (1.0 - 8.0)	-1.990	-3.0 (-4.0 - 0.0)
*Fitness only (n=3)	9.0 (5.0 – 10.0)	2.0 (1.0 – 8.0)		-3.0 (-8.00 - -2.0)
**Depression scale**				
Momenta-Fitness (n=15)	7.0 (3.3 - 11.3)	3.5 (1.0 - 6.0)	-2.908	-3.5 (-6.3 - -0.8)
Momenta only (n=15)	4.0 (1.0 - 6.0)	3.0 (1.0 - 4.0)	-0.762	0.0 (-2.0 - 1.0)
*Fitness only (n=3)	3.0 (0.0 – 8.0)	2.0 (1.0 – 8.0)		1.0 (-8.0 – 5.0)

** Fitness only n=3 therefore median and range reported and no statistical test completed.*

Overall, the results suggested those who participated in the two groups incorporating Momenta, had enhanced physical and psychological health indicators from baseline, whereas those who had only free fitness membership did not. From the small follow-up sample, there is scope to suggest that the combination of Momenta and fitness membership may produce favourable outcomes at 52 weeks.

For peer review only - http://bmjopen.bmj.com/site/about/guidelines.xhtml

Iterative refinements throughout the evaluation process

Here we list a number of implementation adjustments which were made throughout the evaluation process, facilitated via the prototyping framework. Real-time advice from Commissioners was considered during early stages of implementation, regarding the nature of comparison offers (e.g. fitness access) and thus initial design and outcome measurements were adapted prior to referrals being made. To better-target recruitment and change the process of engagement at referral point, entry criteria were altered (BMI $\geq$30 kg/m^2) mid-way through programme delivery. On-site implementation of the service offer was adapted in response to delivery staff feedback: increased resource was made available, for example additional staffing to support delivery for the first wave of referrals. Furthermore, staff were given additional time for Momenta session preparation and session delivery times were extended. Follow-up activities (i.e., text or telephone contact) were implemented by staff during the process, to encourage adherence.

DISCUSSION

We explored 'prototyping', as a cost-effective and time-efficient approach to public health evaluation, via an 'off-the-shelf' weight management programme implemented in a local context of mixed and high deprivation.

Experiences of both referrers and referrals highlighted that HCPs needed to be better-informed and more confident raising weight-related conversations. Whilst patient-led action is desirable, staff reluctance to raise weight issues may mean that opportunities for engagement of less knowledgeable or motivated patients will be missed. The problematic

For peer review only - http://bmjopen.bmj.com/site/about/guidelines.xhtml

positioning of GPs within obesity care has been highlighted previously,[35] with a range of strategies to change HCPs' behaviour resulting in little or no change to patients' weight. A practical training need is highlighted for those working at the patient-practitioner interface, however communication with patients about weight may well be hindered by the 'stigma' attached to obesity.[36] This has wider implications for patient outcomes and requires further exploration through the implementation process. Additionally, HPCs need better understanding of referral-based public health programmes offered. Despite efforts of programme and public health managers, awareness was reportedly low for some referring professionals. We suggest consideration of resource-efficient ways to signpost both HPCs and patients themselves as part of the implementation process.

This programme was delivered across a social gradient in a region with low health indices and areas of high deprivation. Some issues in relation to inequalities and service access for future community-based weight management programmes were highlighted. Only 17% of referrals to Momenta were males. Gender bias in weight management referral has been reported elsewhere,[37-38] and interviews showed that practitioners struggled to raise the topic of weight with male patients. Alternative referral strategies have been employed in other settings in an attempt to overcome this.[39] Marketing in other community spaces, or targeted postal referrals could be explored in future implementation. The initial decision to restrict referral to overweight-only substantially impacted on referral rates, with HCPs and referrals indicating they felt limited until this restriction was reversed. Had this continued, worsening health inequalities may have been an unintended consequence, something to be actively avoided within public health programmes [40.] The roles of, and interactions between, those operating in the 'system' (i.e. the context within which the intervention operates) must be considered at the point of implementation to minimise any impact from unintended

For peer review only - http://bmjopen.bmj.com/site/about/guidelines.xhtml

consequences.[5] In practical terms, this may be through continued dialogue with commissioners, referring professionals and referrals themselves, something which prototyping evaluation allows.

Quantitative data should be interpreted as exploratory, due to the relatively small number of complete cases, however lessons can be learned from these data both in terms of preliminary outcomes and engagement/dropout. Participation in Momenta and Momenta-Fitness resulted in 12-week weight loss for those who completed the programme. Free fitness membership without the weight-management programme was poorly engaged with and did not lead to weight change. A small sub-sample who attended follow-up demonstrated that after one year, weight reductions equivalent to ~4% could be maintained for Momenta-Fitness. We caution that while this might be best interpreted as hypothesis-generating for future evaluations, given these effects emerged despite an underpowered sample it is worth briefly considering potential mechanisms here. Providing free access to fitness facilities alongside the behaviour change programme may allow for continuous and self-driven behaviour change[41] and sustaining optimal changes in adiposity over 12 months in those who remained engaged.[42] Swipe card monitoring during the initial 12-week period indicated that fitness sessions were accessed an average 10 occasions for this group, whereas no access was apparent for Momenta, despite Momenta sessions being held in leisure centres. This could be important for community providers making decisions about delivery location. Both Momenta groups reported improved wellbeing, and reduced anxiety and depression at 12-weeks. The changes observed, though small, could be argued to approach being functionally and clinically meaningful, with a minimal important difference of 1.5 points previously identified for the HADS, for example[43]. The behavioural intervention may drive this effect. This is consistent with previous work reporting co-varying changes in weight loss, depression, and quality of

For peer review only - http://bmjopen.bmj.com/site/about/guidelines.xhtml

life in weight management services.[44] It is unclear whether the primary mechanism was weight loss, or the wider social benefits of participation. Both were valued in the qualitative data. Our preliminary evidence of maintained improvements in wellbeing for these groups at 52 weeks is particularly relevant given previously evidenced associations between poor mental health, and obesity and overweight status.[45] Long-term follow-up rates will need to be considered in future similar programmes and we suggest year-long follow-up (at least) is included as a key programme component from the outset. Consideration should be given to how providers can maintain contact with participants after programme end to increase likelihood of successful follow-up. Potential 'light touch' support after 12 weeks may be helpful and other means of obtaining follow-up data should be explored where service users disengage. Reasons for disengagement might also be usefully explored in future work.

Given that no systematic problems emerged with service-user's experiences of the programme itself, our findings lend support to a streamlined approach to involvement of all stakeholders in programme implementation. We suggest that prototyping demonstrates opportunities for off-the-shelf programmes to be pragmatically moulded to local context, in real-time. Many of the iterative changes made were staff-driven. This demonstrates that real-time consideration of feedback from on-site delivery teams can be important to the implementation process. Some of the adjustments required commissioning action, as they had resource implications; others needed advice from the evaluation team. Interestingly changes made throughout the process generally focused on both staff and participant experience.

Emergence of some negative experiences of referral suggests, however, that prototyping can be problematic without networks or channels for ensuring key outcomes are widely communicated to relevant stakeholders. Overall, the evaluation demonstrated that a balance is

For peer review only - http://bmjopen.bmj.com/site/about/guidelines.xhtml

needed to allow quick and efficient adaptation of off-the-shelf programmes, but with focused professional user engagement in the early stages of development. The prototyping approach had particular utility given that project resources were limited and meant that issues were identified and acted upon rapidly. While the programme may have progressed similarly without this, prototyping provided a greater structure for, and confidence in, on-going refinements. This was achieved via the support provided by academics, public health practitioners and providers. Fundamentally, adopting a prototyping approach enabled the delivery of a new service to an in-need population, alongside the generation of initial evidence of local effectiveness. A minimum of 1 kg weight-loss at 3 months, and 0.7 kg at 12-months have been suggested as thresholds to influence decisions over commissioning of weight-loss services.[46] Our preliminary data suggests that Momenta may have potential to meet or even exceed these thresholds, showing particular promise when implemented in conjunction with free fitness provision.

Demonstrating preliminary effectiveness is of limited use, however, unless a successful programme in one area may be adapted and implemented to suit a different context, for example through sharing local-level knowledge, interactions and behaviours of individuals within different parts of that system.[47] The process for scaling-up of effective health interventions to broader policy and practice takes years[48] and certainly within the obesity literature, has been dominated by initiatives that consider effectiveness but not implementation across specific settings.[49-50] We recommend prototyping might be built into larger public health evaluations providing that the original programme has a sound theoretical basis, and iterative refinement is engaged with by all stakeholders from the outset.

For peer review only - http://bmjopen.bmj.com/site/about/guidelines.xhtml

CONCLUSION

The Momenta programme was experienced positively by those who attended. Issues with the referral process need to be explored further, however other refinements were feasible during delivery. Promising preliminary outcome data for completers of 'Momenta', particularly in conjunction with a free fitness offer, implies potential for the scheme within future commissioning. This evaluation extends the literature by exploring prototyping for a complex problem, community weight-management, in a challenging setting, demonstrating streamlined implementation of an 'off-the-shelf' weight management programme. This resource-effective approach is highly relevant in the context of health inequalities and public health sector funding constraints.

For peer review only - http://bmjopen.bmj.com/site/about/guidelines.xhtml

Acknowledgements: Many thanks to Jordan Bell, Nicole Rowley and Ross Davison, who collected and collated raw quantitative data.

Author Contributions: CDR, CH and EO contributed to design of the quantitative evaluation, data analysis and preparation the final document. LN contributed to design and analyses of the qualitative evaluation component. TF contributed to qualitative design and analysis and preparation of the final document. AL contributed to design of the quantitative evaluation and preparation of the final document.

Funding: The quantitative evaluation component was supported by funding from the Wolfson Research Institute for Health and Wellbeing, Durham University. Amelia Lake is a member of Fuse, the Centre for Translational Research in Publicasters Health (www.fuse.ac.uk). Fuse is a UK Clinical Research Collaboration (UKCRC) Public Health Research Centre of Excellence. Funding for Fuse from the British Heart Foundation, Cancer Research UK, Economic and Social Research Council, Medical Research Council, the National Institute for Health Research, under the auspices of the UKCRC, is gratefully acknowledged. The views expressed in this paper do not necessarily represent those of the funders or UKCRC. The funders had no role in study design, data collection and analysis, decision to publish, or preparation of the manuscript.

Competing interests: CH is a former employee of the Leisure Trust and was instrumental in initiating the evaluation. She was subsequently employed as a research assistant at Durham University, however was not involved in any data collection or entry, only accessing an anonymised database submitted to the University. LN was a Public Health Improvement

For peer review only - http://bmjopen.bmj.com/site/about/guidelines.xhtml

Manager within the Public Health Team and had responsibility for commissioning the Momenta programme. The qualitative evaluation component was submitted in partial fulfilment of her Master's in Public Health at Newcastle University.

Participant consent: Consent was obtained for face-to-face interviews and focus groups. Service users were informed in writing of the nature of the quantitative service evaluation and how to withdraw from it. The presented data are anonymised with risk of identification low.

Ethics approval: Ethical advice was sought from the local Research Manager of North of England Commissioning Support, and this project was classed as a service evaluation in line with National Research Ethics Service guidance.

Data availability statement: No additional data are available because this was a service evaluation.

For peer review only

REFERENCES

1. Haines A, Kuruvilla S, Borchert M. Bridging the implementation gap between knowledge and action for health. (Theme Papers). *Bull World Health Organ* 2004;82:724.

2. Curran GM, Bauer M, Mittman B, *et al*. Effectiveness-implementation Hybrid Designs: Combining Elements of Clinical Effectiveness and Implementation Research to Enhance Public Health Impact. *Med Care* 2012;50:217-26.

3. Sales A, Smith J, Curran G, *et al*. Models, Strategies, and Tools: Theory in Implementing Evidence-Based Findings into Health Care Practice. *J Gen Int Med* 2006;21:S43-S9.

4. Kilbourne AM, Neumann MS, Pincus HA, *et al*. Implementing evidence-based interventions in health care: application of the replicating effective programs framework. *Implement Sci* 2007;2:42.

5. Medical Research Council. Developing and evaluating complex interventions: new guidance. UK: Medical Research Council, 2006. https://mrc.ukri.org/documents/pdf/developing-and-evaluating-complex-interventions/. (Accessed 10 Aug 2018)

6. Oliver K, Kothari A, Mays N. The dark side of coproduction: do the costs outweight the benefits for health research? *Health Research Policy and Systems* 2019;17:33.

For peer review only - http://bmjopen.bmj.com/site/about/guidelines.xhtml

7. Meisinger C, Döring A, Thorand B, *et al*. Body fat distribution and risk of type 2 diabetes in the general population: are there differences between men and women? The MONICA/KORA Augsburg Cohort Study. *Am J Clin Nutr* 2006;84:483-9.

8. Bhaskaran K, Douglas I, Forbes H, *et al*. Body-mass index and risk of 22 specific cancers: a population-based cohort study of 5·24 million UK adults. *Lancet* 2014;384:755-65.

9. Zhang C, Rexrode KM, Van Dam RM, *et al*. Abdominal obesity and the risk of all-cause, cardiovascular, and cancer mortality: sixteen years of follow-up in US women. *Circ* 2008;117:1658-67.

10. Kolotkin RL, Andersen JR. A systematic review of reviews: exploring the relationship between obesity, weight loss and health-related quality of life. *Clin Obes* 2017;7:273-89.

11. Simon GE, Von Korff M, Saunders K, *et al*. Association between obesity and psychiatric disorders in the us adult population. *Arch Gen Psychiatry* 2006;63(7):824-30.

12. Public Health England. *Health matters: obesity and the food environment environment.* UK: Public Health England, 2017. https://www.gov.uk/government/publications/health-matters-obesity-and-the-food-environment/health-matters-obesity-and-the-food-environment--2. (Accessed 10 Aug 2018)

13. World Health Organisation. *Global status report on noncommunicable diseases 2014.* Geneva: World Health Organisation, 2014.

For peer review only

For peer review only - http://bmjopen.bmj.com/site/about/guidelines.xhtml

https://apps.who.int/iris/bitstream/handle/10665/148114/9789241564854_eng.pdf;jsessionid=A043D0E63AACA67752CC3294A1EBD7A3?sequence=1. (Accessed 04 Feb 2019)

14. Bambra C, Hillier F, Cairns J, *et al*. How effective are interventions at reducing socioeconomic inequalities in obesity among children and adults? Two systematic reviews. *Public Health Res* 2015;3.

15. Bull ER, Dombrowski SU, McCleary N, *et al*. Are interventions for low-income groups effective in changing healthy eating, physical activity and smoking behaviours? A systematic review and meta-analysis. *BMJ Open*. 2014;4:e006046.

16. Office for National Statistics. *Dataset: Regional gross value added (balanced) by combined authority in the UK.* UK: Office for National Statistics, 2017. https://www.ons.gov.uk/economy/grossvalueaddedgva/datasets/regionalgrossvalueaddedbalancedbycombinedauthorityintheuk. (Accessed 10 Aug 2018)

17. Public Health England. *Work, worklessness and health: local infographic tool*. UK: Public Health England, 2018. https://www.gov.uk/government/publications/work-worklessness-and-health-local-infographic-tool. (Accessed 10 Aug 2018)

18. Northumberland County Council. *Public Health Service Statement 2017/18.* Northumberland: Northumberland County Council, 2017. https://northumberland.gov.uk/NorthumberlandCountyCouncil/media/About-the-Council/Performance/Service%20Statements%202016/2017-Public-Health-Service-Statement-2017-18.pdf. (Accessed 10 Aug 2018)

For peer review only

19. Public Health England. *Local Authority Health Profiles*. UK: Public Health England, 2019. https://fingertips.phe.org.uk/profile/health-profiles/data#page/0/gid/1938132701/pat/6/par/E12000001/ati/102/are/E06000057/iid/10101/age/169/sex/4. (Accessed 01 Feb 2019)

20. Lister C, Payne H, Hanson CL, *et al*. The Public Health Innovation Model: Merging Private Sector Processes with Public Health Strengths. *Front Public Health* 2017;5:192.

21. Parry GJ, Carson-Stevens A, Luff DF, *et al*. Recommendations for evaluation of health care improvement initiatives. *Acad Pediatr* 2013 Nov-Dec;13(6 Suppl):S23-30.

22. Hawkins J, Madden K, Fletcher A, *et al*. Development of a framework for the co-production and prototyping of public health interventions. *BMC Public Health* 2017;17:689.

23. Stevens VJ, Funk KL, Brantley PJ, *et al*. Design and implementation of an interactive website to support long-term maintenance of weight loss. *J Med Internet Res* 2008;10(1):e1.

24. Discover Momenta Ltd. *Momenta.* UK: Discover Momenta Ltd, 2013: http://discovermomenta.com/. (Accessed 10 Aug 2018)

25. Hanson CL. *Advancing understanding of effective exercise on referral: a mixed methods evaluation of the Northumberland scheme*. PhD Thesis, Durham University, 2017. http://etheses.dur.ac.uk/12162/. (Accessed 10 Aug 2018)

For peer review only - http://bmjopen.bmj.com/site/about/guidelines.xhtml

26. Hanson CL, Allin LJ, Ellis JG, *et al*. An evaluation of the efficacy of the exercise on referral scheme in Northumberland, UK: association with physical activity and predictors of engagement. A naturalistic observation study. *BMJ Open* 2013;3:e002849

27. Sacher PM, Kolotourou M, Chadwick PM, *et al*. Randomized Controlled Trial of the MEND Program: A Family-based Community Intervention for Childhood Obesity. *Obes* 2012;18:S62-S8.

28. Leech N, Onwuegbuzie A. A typology of mixed methods research designs. *Qual Quant* 2009;43:265-75.

29. Marfell-Jones M, Olds T, Stewart A, *et al*. *International Standards for Anthropometric Assessment (2007)*. Potchefstroom, South Africa: International Society for the Advancement of Kinathropometry, 2007.

30. Tong A, Sainsbury P, Craig J. Consolidated criteria for reporting qualitative research (COREQ): a 32-item checklist for interviews and focus groups. Int J Qual. Health Care 2007;19:349–57

31. World Health Organisation. *BMI Classification 2006.* Geneva: World Health Organisation, 2006. http://apps.who.int/bmi/index.jsp?introPage=intro_3.html. (Accessed 10 Aug 2018)

32. Clarke A, Friede T, Putz R, *et al*. Warwick-Edinburgh Mental Well-being Scale (WEMWBS): Validated for teenage school students in England and Scotland. A mixed methods assessment. *BMC Public Health* 2011;11:487.

For peer review only

For peer review only - http://bmjopen.bmj.com/site/about/guidelines.xhtml

33. Zigmond AS, Snaith RP. The Hospital Anxiety and Depression Scale. *Acta Psychiatr Scand* 1983;67:361-70.

34. Braun V, Clarke V. Using thematic analysis in psychology. *Qual Res Psychol* 2006;3:77-101.

35. Flodgren G, Goncalves BDC, Summerbell CD. Interventions to change the behaviour of health professionals and the organisation of care to promote weight reduction in children and adults with overweight or obesity. *Cochrane Database of Syst Rev* 2017;11:CD000984.

36. Phelan SM, Burgess DJ, Yeazel MW, *et al*. Impact of weight bias and stigma on quality of care and outcomes for patients with obesity. *Obes Rev* 2015;16:319-26.

37. Logue J, Allardice G, Gillies M, *et al*. Outcomes of a specialist weight management programme in the UK National Health Service: prospective study of 1838 patients. *BMJ Open* 2014;4:e003747.

38. Lebrun LA, Chowdhury J, Sripipatana A, *et al*. Overweight/obesity and weight-related treatment among patients in U.S. federally supported health centers. *Obes Res Clin Pract* 2013;7:e377-e90.

39. Ahern AL, Aveyard P, Boyland EJ, *et al*. Inequalities in the uptake of weight management interventions in a pragmatic trial: an observational study in primary care. *Br J Gen Pract* 2016;66(645):e258-63.

For peer review only

For peer review only - http://bmjopen.bmj.com/site/about/guidelines.xhtml

40. Adams J, Mytton O, White M, *et al*. Why Are Some Population Interventions for Diet and Obesity More Equitable and Effective Than Others? The Role of Individual Agency. *PLoS Med* 2016;13(4).

41. Mastellos N, Gunn LH, Felix LM, *et al*. Transtheoretical model stages of change for dietary and physical exercise modification in weight loss management for overweight and obese adults. *Cochrane Database Syst Rev* 2014;2:CD008066

42. Garrow JS, Summerbell CD. Meta-analysis: effect of exercise, with or without dieting, on the body composition of overweight subjects. *Eur J Clin Nutr* 1995;49:1-10.

43. Puhan MA, Frey M, Buchi S et al. The minimal important difference of the hospital anxiety and depression scale in patients with chronic obstructive pulmonary disease. Health and Quality of Life Outcomes 2008;6:46:10.1186/1477-7525-6-46

44. Wright F, Boyle S, Baxter K, *et al*. Understanding the relationship between weight loss, emotional well-being and health-related quality of life in patients attending a specialist obesity weight management service. *J Health Psychol* 2013;18:574-86.

45. Somerville R, McKenzie K, Eslami S, *et al*. Poor mental health in severely obese patients is not explained by the presence of comorbidities. *Clin Obes* 2014;5:12-21.

46. Madigan CD, Daley AJ, Lewis AL, et al. Which weight-loss programmes are as effective as Weight Watchers®? Brit J Gen Pract 2014;64(620):e128-e136.

For peer review only - http://bmjopen.bmj.com/site/about/guidelines.xhtml

47. Curtis S, Oven K, Wistow J, *et al*. Adaptation to extreme weather events in complex health and social care systems: The example of older people's services in England. *Environ Plann C: Politics and Space* 2018;36:67-91.

48. Milat AJ, King L, Newson R, *et al*. Increasing the scale and adoption of population health interventions: experiences and perspectives of policy makers, practitioners, and researchers. *Health Res Policy and Systems* 2014;12:18.

49. Jebb S. *Dusting off Foresight's obesity report.* Foresight Blog UK: Government Office for Science, 2017. https://foresightprojects.blog.gov.uk/2017/10/04/dusting-off-foresights-obesity-report/. (Accessed 01 Feb 2019)

50. Wolfenden L, Goldman S, Stacey FG *et al*. Strategies to improve the implementation of workplace-based policies or practices targeting tobacco, alcohol, diet, physical activity and obesity. *Cochrane Database of Syst Rev* 2018;11:CD012439.

For peer review only

For peer review only - http://bmjopen.bmj.com/site/about/guidelines.xhtml

SUPPLEMENTARY FILE 1. Momenta session content

Week	Key topic		Key topic
**Getting started session**	Motivation for weight loss<br>Weight loss goals<br>Differences from other weight management programmes<br>Monitoring		
**Week 1**	**Snacking**<br>Fatty, sugary snacks<br>Calories from snacking<br>Healthy snack choices<br>Healthy eating patterns	**Week 7**	**Fats**<br>Reducing fat<br>Different types of fat<br>Lower-fat cooking techniques<br>Hidden fats
**Week 2**	**Heart rate**<br>Cardiovascular exercise and health<br>Cardiovascular exercise and weight management<br>Recommended amounts of CV activity<br>Heart rate and exercise	**Week 8**	**Internal triggers**<br>Introduction to internal triggers<br>Identifying internal triggers<br>Managing internal triggers
**Week 3**	**Food as fuel**<br>A balanced diet and health<br>Planning meals<br>Fibre	**Week 9**	**Active lifestyles**<br>Physical activity and health<br>Physical activity and weight management<br>Different types of physical activity
**Week 4**	**External triggers**<br>Introduction to external triggers<br>External triggers and over-eating<br>Managing triggers	**Week 10**	**Meals**<br>Positive meal environment<br>Shopping<br>Food labels
**Week 5**	**Strength**<br>Resistance activity and health<br>Resistance activity and weight management	**Week 11**	**Sugars**<br>Sugary foods and drinks and weight loss<br>Alcohol<br>Added sugars<br>Sugary drinks and appetite regulation
**Week 6**	**Breakfast**<br>Eating breakfast<br>Retraining appetite<br>Barriers to eating breakfast<br>Developing new breakfast habits<br>Beyond marketing	**Week 12**	**Eating out**<br>Challenges when eating away from home<br>Management and coping strategies<br>Moving forwards

For peer review only

SUPPLEMENTARY FILE 2.

Healthcare professionals' semi-structured interview guide

Semi Structured Interview Set the interviewee at ease; explain purpose of the interview; offer a better understanding of what the referral process requires to aid tier 2 weight management to be delivered in Northumberland; explanation about how the interview will be recorded; reaffirmation of consent; and how the information will be analysed and stored; rules of confidentiality / anonymity etc.

Questions:

1. Thinking about raising the weight issue, tell me about your experience of discussing weight with patients.

   Prompts

   - *How does it feel to raise weight as an issue?*
   - *Are patients open to discussing weight problems?*
   - *Do you find a difference between genders when discussing weight?*
   - *What helps you, such as the NHS Health Check Programme, to raise the issue of weight?*
   - *What else would help to raise the issue or weight in appointments?*

2. Greater retention is often achieved when patients are ready to change, tell me how you work with / assess patient's readiness to change.

   Prompts

   - *Have you had training around the cycle of change?*
   - *Do you use any specific tools or resources to assess the patient?*
   - *What would help you to assess the patient's readiness to change?*

For peer review only

3. Thinking the information and resources available to you during the referral, do you feel you had enough information and resources to encourage patient take up of the programme?

Prompts

- *Did you have enough background information?*
- *Were the referral forms suitable / capture all the information required?*
- *Were the patient leaflets / resources suitable?*
- *Were there questions or issues raised that couldn't be answered?*
- *Was the process easy to use?*
- *What else could help you to make referrals to weight management programmes?*

4. Thinking about after you referred the patient, what happened next? (excluded after pilot)

Prompts

- *Did you get feedback from the weight management programme on the progress of your patient?*
- *Did your patients achieve weight loss?*
- *Did your patient come back and talk about their experience?*

5. What things are most likely to prevent you from making the referral a weight management programme, either commercial or Public Health funded?

Prompts

- *Are there barriers that you perceive, such as cost to the patient?*
- *Are you concerned with raising the weight issue?*
- *Is it a time factor if the patient has an appointment for anything other than a weight issue?*
- *What would help you to overcome the barriers that prevent you from making the referral?*

For peer review only

6. Is there anything else that you would like to tell me about your expectations and experiences of the weight management programme?

Focus Group Topic Guide

Set group at ease; explain purpose of the focus group; offer a better understanding of what works for people in terms of tier 2 weight management and what doesn't, aiding development of an effective programme for Northumberland residents; explanation about how the focus group will be recorded; reaffirmation of consent; and how the information will be analysed and stored; rules of confidentiality / anonymity etc.

1. Tell me a bit about what sort of weight management activity you have taken part in, in the past.

Prompts

- *What influence have others had on your weight management?*
- *Do you have any particular likes/dislikes of physical activity/managing weight/nutrition*
- *Has there been anything else that has influenced your management of weight?*

2. So thinking about the weight management programme you have undertaken, how did you find out about it?

Prompts

- *Who / what motivated you to attend?*
- *What made you decide that this is the right time to look at managing your weight?*
- *Did the time of year make a difference?*

For peer review only

3. Thinking about your experience of when you were referred to the weight management programme, how did you find the process?

Prompts

- *What type of health professional referred you? (GP / Practice Nurse)*
- *Did you specifically attend Primary Care to discuss your weight?*
- *How was weight raised?*
- *What did the referrer explain to you about the programme? Did you get enough information?*
- *How long was it from your referral from Primary Care to the first assessment in the weight management programme; was this what you expected? Were you still motivated?*

4. How did you feel about being referred?

- *Prompts*
- *How confident did you feel about taking part in the programme?*
- *Was there anything that you were particularly looking forward to?*
- *Was there anything that you were worried about?*

5. What did you hope to achieve by taking part in the weight management programme?

Prompts

- *What were your expectations when you start attending the scheme?*
- *Have there been changes to your health that you expected happen as a result of participation?*
- *How quickly did you expect to see these changes? And did this happen?*

6. Thinking about after you were referred, what happened next?

Prompts

For peer review only

- *How long after referral did it take to be contacted by the Active Northumberland?*
- *What information did you receive prior to the initial consultation?*
- *How comfortable did you feel coming to the initial consultation?*

7. What influenced you most to attend the weight management programme?

Prompts

- *What did you expect from the staff?*
- *How important to you were changes in health or weight?*
- *Why were the influences raised important?*

8. What things were most likely to prevent you from attending the programme?

Prompts

- *Tell me about any worries you might have had about health issues.*
- *Tell me about any other things, such as other commitments, that might have stopped you from attending*
- *Did any of these issues arise? How did you overcome these issues?*

9. Now that you have completed the programme, tell me how did you felt about undertaking the weight management programme?

Prompts

- *Did you achieve the health / weight outcomes you expected?*
- *Why do you think it worked or not for you?*
- *Do you feel you now have the tools to continue to make positive lifestyle choices?*
- *Is there something that will prevent you to continue to make positive lifestyle choices?*

For peer review only

10. Is there anything else that you would like to tell me about your expectations and experiences of the weight management programme?

For peer review only

COREQ GUIDELINES REPORTING CHECKLIST: Prototyping for public health in a local context: a streamlined evaluation of a community-based weight management programme (Momenta), Northumberland, UK

No	Item	Guide questions/description	Information	Reported in manuscript (Section, page no.)
	**Domain 1: Research team and reflexivity**			
	Personal Characteristics			
1	Interviewer/ facilitator:	Which author/s conducted the interview or focus group?	LN	Qualitative evaluation component, page 10
2	Credentials	What were the researcher's credentials?	LN: part of her Public Health Masters degree	Qualitative evaluation component, page 10
3	Occupation	What was their occupation at the time of the study?	LN: Masters student and employed as a member of the Northumberland public health team at the time of the evaluation.	Qualitative evaluation component, page 10
4	Gender	Was the researcher male or female?	Female (referred to as her)	Qualitative evaluation component, page 10
5	Experience and training	What experience or training did the researcher have?	LN: Masters in Public Health (which contained qualitative methods training), mentored by TF, an experienced qualitative researcher.	Qualitative evaluation component, page 10
	Relationship with participants			
6	Relationship established	Was a relationship established prior to study commencement?	No. Practice managers from all six referring surgeries were sent an invitation for staff to take part.	Qualitative evaluation component, page 10
7	Participant knowledge of the interviewer	What did the participants know about the researcher? *e.g. personal goals, reasons for doing the research*	LN informed HCP participants about her employment status and that the study aimed to understand implementation issues.<br><br>Programme participants were invited to participate in a series of focus groups at programme-end to explore experiences	Qualitative evaluation component, page 10-11
8	Interviewer characteristics	What characteristics were reported about the interviewer/facilitator? *e.g. Bias, assumptions, reasons and interests in the research topic*	Not discussed	N/A

	**Domain 1: Study design**			
	Theoretical framework			
9	Methodological orientation and Theory	What methodological orientation was stated to underpin the study? *e.g. grounded theory, discourse analysis, ethnography, phenomenology, content analysis*	Qualitative data were audio-recorded and transcribed by LN using a thematic process	Data analysis, page 12
	Participant selection			
10	Sampling	How were participants selected? *e.g. purposive, convenience, consecutive, snowball*	All HCPs involved were invited: Practice managers from all six referring surgeries were sent an invitation for staff to take part<br><br>Programme participants: for the first wave of referrals, all (n = 39) were invited to participate in a series of focus groups.	Qualitative evaluation component, page 10-11
11	Method of approach	How were participants approached? *e.g. face-to-face, telephone, mail, email*	HCPs: by email<br><br>Programme participants: written information handed out during the first session	Qualitative evaluation component, page 10-11
12	Sample size	How many participants were in the study?	5 HCPs<br>13 Intervention participants	Results, page 18<br>Results, page 18
13	Non-participation	How many people refused to participate or dropped out? Reasons?	HCPs: 84 invited, 5 participated.<br><br>Programme participants: 39 invited, 13 participated<br>Reasons for refusal not documented	Qualitative evaluation component, page 10-11<br>and<br>Results, page 15<br>Results, page 18
	Setting			
14	Setting of data collection	Where was the data collected? *e.g. home, clinic, workplace*	HCPs: in referring surgeries<br><br>Programme participants: in the leisure centres	Results, page 15<br><br><br>Results, page 18
15	Presence of non-participants	Was anyone else present besides the participants and researchers?	Not stated	N/A
16	Description of sample	What are the important characteristics of the	HCPs: role reported	Results, page 15<br><br>Results, page 18

For peer review only

		sample? *e.g. demographic data, date*	Programme participants: programme group and weight loss status reported<br><br>Date range for interviews reported	Qualitative evaluation component, page 10
	Data collection			
17	Interview guide	Were questions, prompts, guides provided by the authors? Was it pilot tested?	Semi-structured interview guide used. Pilot tested.<br><br>Guides provided as supplementary file	Qualitative evaluation component, page 10-11<br><br>Supplementary file 2
18	Repeat interviews	Were repeat interviews carried out? If yes, how many?	No not applicable to study design	N/A
19	Audio/visual recording	Did the research use audio or visual recording to collect the data?	Yes, the interviews were audio recorded	Data analysis, page 12
20	Field notes	Were field notes made during and/or after the interview or focus group?	Notes taken from focus groups helped to contextualise developing themes	Data analysis, page 12
21	Duration	What was the duration of the interviews or focus groups?	HCPs average length reported: 26 minutes<br><br>Programme participants: range reported: 26-44 minutes	Qualitative evaluation component, page 10<br><br>Qualitative evaluation component, page 11
22	Data saturation	Was data saturation discussed?	No	N/A
23	Transcripts returned	Were transcripts returned to participants for comment and/or correction?	No	N/A
	**Domain 3: analysis and findings**			
	Data analysis			
24	Number of data coders	How many data coders coded the data?	N=2 (LN and TF)	Data analysis, page 12
25	Description of the coding tree	Did authors provide a description of the coding tree?	Yes key themes described at beginning of HCP qualitative results section and at beginning of programme participant qualitative results section	Results, page 15<br><br>Results, page 18
26	Derivation of themes	Were themes identified in advance or derived from the data?	Identified from data	Data analysis, page 12

For peer review only

27	Software	What software, if applicable, was used to manage the data?	Not stated	N/A
28	Participant checking	Did participants provide feedback on the findings?	No	N/A
	Reporting			
29	Quotations presented	Were participant quotations presented to illustrate the themes / findings? Was each quotation identified? e.g. participant number	Yes, participants identified using a participant label	Results, page 15-19
30	Data and findings consistent	Was there consistency between the data presented and the findings?	Themes were illustrated by participant quotations	Results, page 15-19
31	Clarity of major themes	Were major themes clearly presented in the findings?	Themes identified and presented under sub headings for both HCPs and programme participants	Results, page 15-19
32	Clarity of minor themes	Is there a description of diverse cases or discussion of minor themes?	For HCPs minor themes highlighted under additional barriers to engagement	Results, page 17

For peer review only
